# Effectiveness of telehealth versus in-person care during the COVID-19 pandemic: a systematic review

**DOI:** 10.1038/s41746-024-01152-2

**Published:** 2024-06-15

**Authors:** Elham Hatef, Renee F. Wilson, Allen Zhang, Susan M. Hannum, Hadi Kharrazi, Stacey A. Davis, Iman Foroughmand, Jonathan P. Weiner, Karen A. Robinson

**Affiliations:** 1 Division of General Internal Medicine, Department of Medicine, Johns Hopkins School of Medicine, Baltimore, MD USA; 2 Center for Population Health Information Technology, Department of Health Policy and Management, Johns Hopkins Bloomberg School of Public Health, Baltimore, MD USA; 3 Johns Hopkins Evidence-based Practice Center, Department of Health Policy and Management, Johns Hopkins Bloomberg School of Public Health, University, Baltimore, MD USA; 4 Department of Health, Behavior, and Society, Johns Hopkins Bloomberg School of Public Health, Baltimore, MD USA; 5 Department of Health Policy and Management, Johns Hopkins Bloomberg School of Public Health, Baltimore, MD USA

**Keywords:** Health care, Health services

## Abstract

In this systematic review, we compared the effectiveness of telehealth with in-person care during the pandemic using PubMed, CINAHL, PsycINFO, and the Cochrane Central Register of Controlled Trials from March 2020 to April 2023. We included English-language, U.S.-healthcare relevant studies comparing telehealth with in-person care conducted after the onset of the pandemic. Two reviewers independently screened search results, serially extracted data, and independently assessed the risk of bias and strength of evidence. We identified 77 studies, the majority of which (47, 61%) were judged to have a serious or high risk of bias. Differences, if any, in healthcare utilization and clinical outcomes between in-person and telehealth care were generally small and/or not clinically meaningful and varied across the type of outcome and clinical area. For process outcomes, there was a mostly lower rate of missed visits and changes in therapy/medication and higher rates of therapy/medication adherence among patients receiving an initial telehealth visit compared with those receiving in-person care. However, the rates of up-to-date labs/paraclinical assessment were also lower among patients receiving an initial telehealth visit compared with those receiving in-person care. Most studies lacked a standardized approach to assessing outcomes. While we refrain from making an overall conclusion about the performance of telehealth versus in-person visits the use of telehealth is comparable to in-person care across a variety of outcomes and clinical areas. As we transition through the COVID-19 era, models for integrating telehealth with traditional care become increasingly important, and ongoing evaluations of telehealth will be particularly valuable.

## Introduction

The shift to telehealth during the COVID-19 pandemic since the spring of 2020 impacted not only those with COVID-19^1^ but all other persons in contact with the healthcare system during the pandemic. This shift resulted in a precipitous drop in the rates of patients seeking in-person care accompanied by a marked increase in telehealth encounters^2,3^ and presented an opportunity for a natural experiment of telehealth services compared to in-person care during the pandemic and beyond. While the patterns of telehealth use have changed beyond the initial months of telehealth implementation, the adoption trajectory of these technologies has been forever affected^4–7^. Assessing the effectiveness of telehealth is needed to help guide future strategies and actions by policymakers, payers, and professional societies. The assessment of telehealth use during the COVID-19 pandemic is particularly valuable since the pandemic provided the opportunity to assess the use of telehealth services in comparison and as a replacement for in-person care. This information can help to develop best practices for the use of telehealth to maximize the value to patients served by the U.S. healthcare system.

Several narrative reviews have synthesized evidence on the use of telehealth during the pandemic^8–10^. While these reviews offer initial evidence about the characteristics of telehealth expansion during the pandemic, they are all limited in scope and methodology. These reviews do not systematically assess the outcomes of telehealth in comparison with in-person care and each review focuses on one aspect of service expansion (e.g., characteristics of the clinical providers or patients, patient/provider satisfaction, and implementation challenges). Thus, there remains a need to perform a comprehensive synthesis of available evidence on the effectiveness of telehealth, including its potential benefits and harms during the COVID-19 pandemic. Such a review needs to focus on both the early months of the COVID-19 era when telehealth services were being implemented and the later months when those services were established and maintained.

In this review, we assess the effectiveness of telehealth compared with in-person care among patients who received care during the COVID-19 pandemic. We sought to compare outcomes across different patient populations, clinical areas, and healthcare settings.

## Results

### Results of the search

**Fig. 1:**
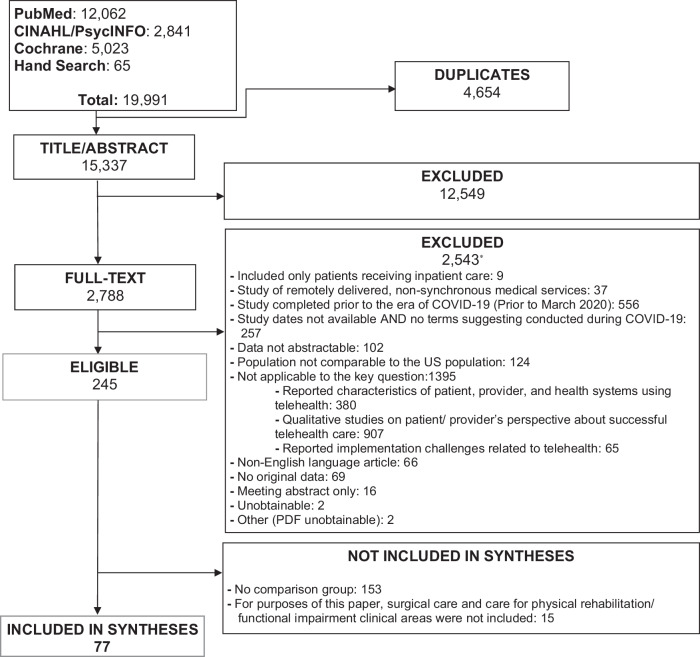
Systematic review flow diagram. The diagram depicts the evidence search and selection including number of included and excluded studies and the reason for exclusion. *The sum of excluded articles exceeds 2543 because reviewers were not required to agree on reasons for exclusion.

**Fig. 2:**
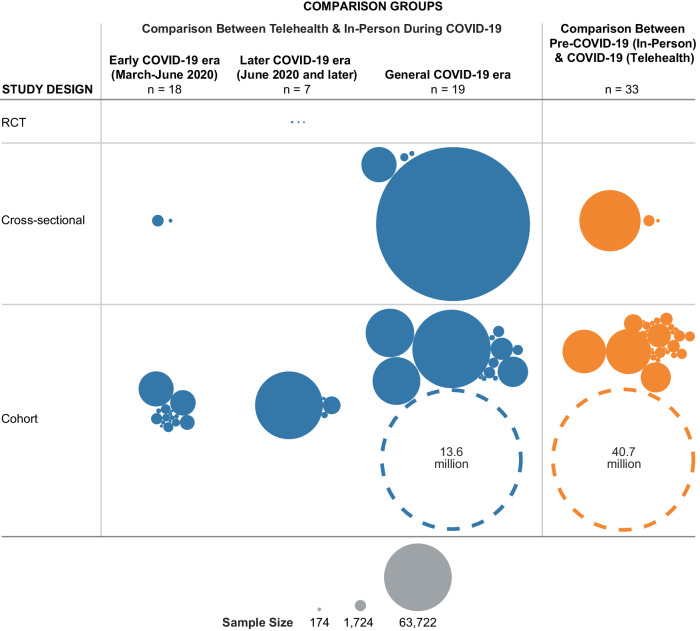
Characteristics of included studies. Characteristics are presented by comparison groups, study timeline, study design, and sample size. The dotted circles present two studies with very large sample sizes. The size of the circle is not proportional to the study sample size. COVID-19 coronavirus disease 2019, RCT randomized clinical trial.

We identified 15,337 unique citations of which 77 studies were eligible (Fig. 1). The majority of the studies were observational studies (96%) and about one-third had fewer than 300 participants (32%). Almost half of the studies (43%) compared telehealth care during the COVID-19 era to in-person care before the pandemic (Fig. 2). Among studies that reported the healthcare setting (74%) about one-third were performed in a small single facility (16 studies: 28%) and only 4 studies (7%) had a nationally representative study sample. Only one-third of the studies (26: 34%) adjusted their results for factors such as the demographic, socioeconomic, or clinical characteristics of the study population (Supplementary Table 2A–C).

**Fig. 3:**
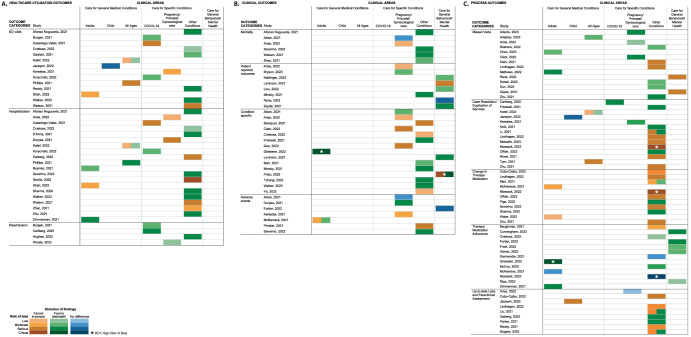
Direction of findings and risk of bias assessment for included studies. The assessment is reported by outcome categories and clinical areas including healthcare utilization outcomes (**A**), clinical outcomes (**B**), and process outcomes (**C**). We assessed the risk of bias using the Cochrane Risk of Bias Tool, Version 2, for randomized controlled trials. The tool categorized the risk of bias into low, some concerns, and high. All three randomized clinical trials included in this review were assessed to have a high risk of bias. COVID-19 coronavirus disease 2019, ED emergency department, RCT randomized clinical trial.

We identified no eligible studies for almost half of the comparisons across the 12 outcomes and 7 clinical areas (39 out of 84 (46%) comparisons, see Supplementary Table 3). Healthcare utilization and process outcomes were the most commonly reported outcome categories. Care for specific conditions, other than pregnancy or COVID-19, was the most studied clinical area, with data for all 12 outcomes assessed. We identified studies assessing only 3 outcomes for general medical care of children, 4 outcomes were assessed for general care for all ages, and studies assessed only 5 outcomes for general behavioral/mental health (refer to Supplementary Tables 4A–C, 5A–D, and 6A–E for the details of results). The majority of studies (44 out of 74: 59% of observational studies and all 3 RCTs) were judged to have a serious or high risk of bias (Fig. 3 and Supplementary Table 7A, B). The following sections present findings by categories of outcomes and clinical areas.

### Healthcare utilization

We classified 3 outcomes as healthcare utilization: ED visits, hospitalization, and readmission. We found no studies addressing these outcomes for one of the seven clinical areas (mental health).

We identified 14 observational studies that compared ED visit rates for telehealth versus in-person care across 6 of the 7 clinical areas (Fig. 3A, Supplementary Table 8 – Top Tier, and Supplementary Fig. 1A)^11–24^. Only one study (n = 1769) addressed general medical care for children reporting no difference in ED visit rates between in-person and telehealth care. For three of the clinical areas, the use of telehealth was associated with an increase in ED visits: for general medical care among adults one study and care for specific conditions, three studies on COVID-19, and one study on pregnancy/prenatal/gynecological care reported lower ED visits among those in the in-person groups versus those in the telehealth groups. For two clinical areas, evidence favored telehealth versus in-person care: for care for other specific conditions, six studies reported higher ED visits among those in the in-person groups versus those in the telehealth groups. For general medical care, of all ages, the largest study (n = 607,573) reported higher rates of ED visits for those with acute conditions receiving telehealth but similar to lower rates for telehealth visits for those with chronic ambulatory care-sensitive conditions.

We identified 20 observational studies that compared hospitalization rates for telehealth versus in-person care across 5 of the clinical areas (Fig. 3A, Supplementary Table 8 – Top Tier, and Supplementary Fig. 1A)^11–13,15,18,20–22,24–35^. For adult patients who received care for general medical conditions, evidence from three studies suggested that those who received an initial telehealth visit had similar hospitalization rates compared with those who received in-person care. For three of the clinical areas, those receiving telehealth had a higher hospitalization rate than those receiving in-person care. For instance, for care for specific conditions (i.e., COVID-19 care and women who receive specialized pregnancy/prenatal/gynecological care) four studies reported a lower hospitalization rate among those in the in-person groups versus those in the telehealth groups. For people of all ages receiving general medical care, the largest study reported similar to higher rates of hospitalization for those with acute conditions receiving telehealth but lower rates for telehealth visits for those with chronic ambulatory care-sensitive conditions. For care for other specific conditions evidence from 11 studies favored telehealth care.

We identified 4 small observational studies that compared readmission rates for telehealth versus in-person care across 3 of the clinical areas (Fig. 3A, Supplementary Table 8 – Top Tier, and Supplementary Fig. 1A)^14,36–38^. Differences, if any, in readmission rates between telehealth and in-person care were small and/or not clinically meaningful.

### Clinical outcomes

Four outcomes were considered clinical outcomes. We identified no studies addressing general medical conditions in children or all age populations, as well as no studies addressing care for COVID-19.

We identified 6 mostly small observational studies that evaluated mortality rates for telehealth versus in-person care across 2 of the clinical areas (Fig. 3B, Supplementary Table 8 – Middle Tier, and Supplementary Figure 1B)^18,21,28,29,35,39^. For women who received specialized pregnancy/prenatal/gynecological care differences, if any, in mortality rates between telehealth and in-person care were small and/or not clinically meaningful (N = 2). For care for specific conditions (e.g., patients with cardiac conditions and cancer), evidence from 4 studies favored telehealth care compared with in-person care.

We identified 8 mostly small observational studies that evaluated patient-reported outcomes for telehealth versus in-person care across 3 of the clinical areas (Fig. 3B, Supplementary Table 8 – Middle Tier, and Supplementary Fig. 1B)^28,40–46^. Studies varied in the type of patient-reported outcomes and instruments used. For women who received specialized pregnancy/prenatal/gynecological care evidence from two studies favored in-person care. One study on care for other specific conditions reported better outcomes for telehealth care. For general behavioral/mental healthcare, evidence from 5 studies favored telehealth care. The studies used different questionnaires with varying degrees of accuracy to assess the mental health of their patients, which may have impacted the differences detected between the two groups.

We identified 13 mostly small observational studies^20,24,28,39–41,47–53^ and 2 RCTs^54,55^ that evaluated a variety of general medical and condition-specific clinical outcomes across 4 of the clinical areas (Fig. 3B, Supplementary Table 8 – Middle Tier, and Supplementary Fig. 1B). Studies varied in the type of clinical outcomes they assessed. For adult patients who received care for general medical conditions, evidence from one RCT supported worse clinical outcomes for those receiving an initial telehealth visit compared with those who received an initial in-person visit. For two of the clinical areas: for women who received specialized pregnancy/prenatal/gynecological care (N = 4 studies) and for care for other specific conditions (N = 8 studies), evidence regarding the effectiveness of telehealth vs. in-person care varied due to different patient populations and clinical areas assessed across included studies. However, the difference between in-person and telehealth groups was larger and clinically meaningful in studies favoring in-person visits. For one clinical area: those receiving general behavioral/mental healthcare (N = 2 studies including one RCT) telehealth use resulted in no clinically meaningful difference or more improvement in clinical outcomes.

We identified 7 mostly small observational studies^16,27,35,39,56–59^. Studies reported a variety of adverse events across 4 of the clinical areas (Fig. 3B, Supplementary Table 8 – Middle Tier, and Supplementary Figure 1B). For three of the clinical areas: in care for adults with general medical conditions (N = 1 study), for women who received specialized pregnancy/prenatal/gynecological care (N = 3 studies), and in care for other specific conditions (N = 2 studies) telehealth use resulted in fewer adverse events. However, the differences in adverse event rates between telehealth and in-person care were small and/or not clinically meaningful. There were no differences noted in adverse event rates between telehealth and in-person care for behavioral/mental healthcare (N = 1 study).

### Process outcomes

Five outcomes were considered process outcomes, identifying studies addressing all clinical areas.

We identified 14 mostly small observational studies that evaluated missed visit rates across 5 of the clinical areas (Fig. 3C, Supplementary Table 8 – Bottom Tier, and Supplementary Fig. 1C)^28,31,43,45,52,59–68^. For general medical care among adults (N = 2 studies) telehealth resulted in a higher rate of missed visits. For three other clinical areas: in general medical care among patients of all ages (N = 1 study), for women who received specialized pregnancy/prenatal/gynecological care (N = 3 studies), and in care for other specific conditions (N = 6 studies) telehealth use resulted in a lower rate of missed visits. For general behavioral/mental health (N = 2 studies), telehealth resulted in higher rates of cancellations and no-shows but the differences were small.

We defined case resolution as a patient’s chief complaint being addressed in an initial visit and duplication of service as the need for a follow-up visit (e.g., telehealth followed immediately by an in-person visit). We identified 13 observational studies^12,16,23,31,36,47,61,69–74^ and 1 RCT^75^ that evaluated case resolution and duplication of services across 5 of the clinical areas (Fig. 3C, Supplementary Table 8 – Bottom Tier, and Supplementary Fig. 1C). Only one study (n = 1769) addressed general medical care for children reporting no difference in follow-up visits between in-person and telehealth care. For general medical care, all ages (N = 2 studies), the larger study (n = 607,573) reported higher follow-up rates for those with acute conditions receiving telehealth but lower rates for telehealth visits for those with chronic ambulatory care-sensitive conditions. For two of the clinical areas: COVID-19 care (N = 1 study) and specialized pregnancy/prenatal/gynecological care (N = 1 study) the evidence was insufficient to conclude. For care for other specific conditions (N = 9 studies including one RCT with a small sample size of 48 patients), the evidence regarding the effectiveness of telehealth vs. in-person care varied due to different patient populations and clinical areas. The difference between in-person and telehealth groups was larger and clinically meaningful in studies favoring telehealth care.

We identified 10 observational studies and 1 RCT that reported change in therapy/medication for only 2 of the clinical areas (Fig. 3C, Supplementary Table 8 – Bottom Tier, and Supplementary Figure 1C)^30,31,35,48,56,61,73,75–78^. Adult patients who received telehealth care for general medical conditions (N = 2 studies) and adults receiving care for specific conditions (N = 9 studies including one RCT study with a small sample size of 48 patients) experienced lower rates of change in therapy/medication than those receiving in-person care. However, the differences in the rates of change in therapy/medication between telehealth and in-person care were mostly small and clinically not meaningful.

We identified 11 mostly small observational studies and 2 RCTs that evaluated treatment/medication adherence for telehealth versus in-person care across 3 of the clinical areas (Fig. 3C, Supplementary Table 8 – Bottom Tier, and Supplementary Fig. 1C)^20,25,54,56,58,75,79–85^. The studies used different definitions of treatment/medication adherence. For two of the clinical areas: general medical care among adults (N = 3 studies) and general behavioral/mental healthcare (N = 3 studies), those receiving an initial telehealth visit had higher rates of therapy/medication adherence compared with those who received in-person care. For care for specific conditions (N = 5 studies including one RCT with a small sample size of 48 patients), evidence regarding the effectiveness of telehealth vs. in-person care varied due to different patient populations and clinical areas assessed across included studies. However, the difference between in-person and telehealth groups was larger and clinically meaningful in studies favoring in-person visits.

We identified 9 mostly small observational studies reporting rates of up-to-date labs and paraclinical assessment including imagining and pathology assessment for telehealth versus in-person care for only 3 of the clinical areas (Fig. 3C, Supplementary Table 8 – Bottom Tier, and Supplementary Fig. 1C)^19,23,28,34,61,77,86–88^. Only one study (n = 1769) addressed general medical care for children reporting lower rates of up-to-date labs and paraclinical assessment among those who received initial telehealth care. Only one study (n = 104) addressed specialized pregnancy/prenatal/gynecological care reporting similar rates of up-to-date labs and paraclinical assessment for those with an initial telehealth visit compared with those who received in-person care. For care for specific conditions (N = 7 studies), evidence regarding the effectiveness of telehealth vs. in-person care varied due to different patient populations and clinical areas. However, the difference between in-person and telehealth groups was larger and clinically meaningful in studies favoring in-person care.

## Discussion

We performed a systematic review to compare telehealth to in-person care during the COVID-19 era. Overall, we found the available evidence on the effectiveness of telehealth versus in-person care during the pandemic weak and heterogeneous: the included studies were mostly observational studies with small sample sizes performed in a small single facility. The majority of the studies had a serious or high risk of bias and did not adjust their results for factors such as the demographic, socioeconomic, or clinical characteristics of the study population. Studies reported a wide range of outcomes, measured in multiple ways, among patients being treated for a variety of conditions. Outcomes such as those related to healthcare utilization measures and clinical areas such as care for specific conditions, other than COVID-19 or pregnancy, were more commonly reported. However, fewer studies were addressing clinical areas such as care for general behavioral/ mental health despite the more common use of telehealth services in this area during the pandemic^12,89^.

While the broad scope of studies bolstered the representativeness of the full range of care delivered during this unique period, the breadth of the evidence limited our ability to make any general statements. Further, in the face of small observational studies across heterogeneous study populations and to take into account differences in the clinical areas, patient/provider characteristics, comparison groups, study timelines, and type of assessment performed during the visits across the small number of studies addressing each outcome-condition comparison we refrained to make an overall conclusion about the performance of telehealth versus in-person visits. Thus, we categorized the studies based on their reported outcomes (12 outcome categories), and for each category we broke down the evidence into several clinical areas (7 clinical areas), resulting in 84 unique outcome-condition comparisons, for 45 of which we identified eligible studies. We drew conclusions for each outcome category and clinical area separately relying on the available evidence but almost uniformly had low confidence for outcomes, and clinical areas where we were able to draw conclusions. Moreover, we were unable to draw conclusions due to insufficient evidence for two comparisons for process outcome of *Case Resolution/Duplication of Services* among those receiving care for COVID-19 and pregnancy.

We found conflicting results about the effect of telehealth on healthcare utilization outcomes in comparison with in-person care. Overall, the differences between telehealth and in-person care were small and not clinically meaningful. The short follow-up periods across different studies may have resulted in these small differences. We are unable to determine the reasons for any differences in healthcare utilization. It could be that patients receiving telehealth care were more likely to have severe conditions that led to necessary utilization such as ED visits and hospitalization, that those patients were more likely to seek unnecessary care, or that telehealth was an inadequate mode of care delivery for some patients such as those requiring specialized care for women’s health and COVID-19.

Clinical outcomes were generally similar between telehealth and in-person care. Any differences in mortality rates and reported adverse events in different clinical areas were mostly small and/or not clinically meaningful. The limited sample sizes and short study follow-up periods may have resulted in the detection of small or no differences between the two groups. For patient-reported outcomes, telehealth may be a convenient mode of care delivery for specific clinical conditions, which require fewer interventions by the provider. However, it may be less suitable and less desirable for therapies requiring the development of rapport and ongoing communication between the patient and the care team. For condition-specific clinical outcomes, evidence regarding the effectiveness of telehealth vs. in-person care varied due to different patient populations, clinical areas, and outcomes assessed across included studies as well as different follow-up periods. Telehealth may not be a desirable mode of care delivery for adult patients who received care for general medical conditions, women who received specialized pregnancy/prenatal/gynecological care, and for care for other specific conditions. However, for general behavioral/mental healthcare evidence favored telehealth care.

For process outcomes, evidence supported a mostly lower rate of missed visits, lower rate of change in therapy/medication, and higher rates of therapy/medication adherence, but also a lower rate of up-to-date labs and paraclinical assessment among patients receiving an initial telehealth visit. Among patients who received general medical care for an acute condition, those who received telehealth care may have lower rates of care resolution in their initial visit and, thus higher rates of follow-up visits. However, among patients who received general medical care for a chronic condition, those who received telehealth care may have higher rates of care resolution in their initial visit and, thus lower rates of follow-up visits. Lower rates of up-to-date labs and paraclinical assessment for patients who receive care for specific conditions in an initial telehealth visit suggest that telehealth care may not be an adequate mode of care delivery when care beyond the initial assessment of the clinical condition is required or when the provider needs to decide on the treatment plan or medications. Further study is needed to ensure that telehealth is appropriate for patients with complex conditions or those requiring a greater variety of health services, including hands-on physical exams.

We compared our findings with the evidence from other narrative reviews on the characteristics of telehealth expansion during the pandemic^8–10,90–93^. Our findings were in some instances in contrast with other reviews. For example, a report by the NCQA Taskforce on Telehealth Policy analyzed evidence from several large health systems and payors in late 2020 and found that the use of telehealth, before and during the COVID-19 pandemic, reduced urgent and ED care, as well as the use of expensive or often overused services, such as imaging^92^. Another systematic review before the pandemic also provided evidence supporting the use of telehealth as a way to reduce acute care utilization (e.g., readmissions, length of stay, ED visits)^91^. We found mixed results on healthcare utilization outcomes, which varied by the clinical condition of the patient. A scoping review including studies from pre-pandemic and during the COVID-19 pandemic on maternal health (only 9 out of 42 included studies were published during the pandemic) concluded that telehealth maternal care as a replacement or supplement to in-person care may result in similar, and sometimes better, clinical outcomes and patient satisfaction compared with in-person care^90^. We found mixed results related to the use of telehealth vs. in-person care for women who received specialized pregnancy/prenatal/gynecological care. This review included studies from pre-pandemic, which may explain the difference in their findings with those from our review. A review during the pandemic assessed different applications, challenges, motivations, and recommended solutions for the use of telehealth during the first year of the pandemic (up to July 2021). The review did not synthesize evidence on the effectiveness of telehealth. However, they reported similar findings to our review in terms of the use of telehealth for telemonitoring and telemanagement of mostly chronic conditions such as diabetes and cancer^93^.

Our ability to synthesize the available evidence was limited by a lack of a unified approach to defining and assessing outcomes. The heterogeneity of the outcomes and outcome measures reported, as well as the variety of clinical areas and patient/provider characteristics, further limited our ability to synthesize the evidence. The current standard telehealth quality measures were developed before the COVID-19 era^94,95^, and thus may not fully apply at this time when telehealth is now one of the dominant care modalities. Reviewing these pre-COVID-19 era telehealth performance measures to identify a set of process and outcome measures that are appropriate for the circumstances of the COVID-19 era may help to conduct studies with generalizable results across different populations.

Studies varied in their comparison groups; some compared telehealth with in-person visits in the COVID-19 era and others compared the use of telehealth in the COVID-19 era to services provided in the pre-COVID-19 era, assuming those services were predominantly in-person. There were significant differences between patients seen before the pandemic and those who were seen in person or via telehealth during the pandemic. Moreover, the variations in the timeline of different studies further limited our ability to synthesize the available evidence. At the peak of the COVID-19 pandemic, a telehealth visit may have been a necessary precondition for subsequently accessing an in-person visit or the only available service. Thus, telehealth vs. in-person care was not an either/or decision for patients to make. This consideration may have impacted the patterns of telehealth use during each surge of COVID-19. The heterogeneity of comparison groups limited our ability to make a general conclusion about the impact of telehealth vs. in-person care. The evidence would benefit from well-designed studies with concurrent comparison groups.

Our review had several limitations. Given the intent to assess the effectiveness of telehealth in comparison with in-person care during the pandemic, we focused on studies conducted during the COVID-19 era. This was a very unstable time to implement telehealth programs and to evaluate these, then, novel programs. It is possible, if not likely, that a repeated study in the same setting would have different results. We limited our review to telehealth which was provided synchronously (supporting two-way communication between a patient and a provider) in the outpatient/ambulatory or ED setting. Telehealth has also seen increased use in other settings, such as in-patient care and provider-provider communications. In addition, the growing body of evidence on the use of asynchronous virtual care, such as through wearable devices, was beyond the scope of this review. Focusing on evidence relevant to the U.S. and countries with a population similar to that of the U.S. may have impacted the generalizability of our findings.

In terms of future research, evidence about telehealth would be more useful for practice and policy decisions if the quality of data and studies were better. There is a need for a clear definition of telehealth and other modes of virtual care delivery, the context in which those services are implemented, and the usual or alternative models of care used for comparison. The current evidence relies on pre-post data from single-site studies; more informative research would include multisite studies and studies conducted across different private and public health systems. Finally, research is particularly needed on the effectiveness of telehealth for clinical areas with limited prior evidence but rapid expansion during a pandemic (e.g., primary care visits).

For telehealth to be effective as a stand-alone substitute or in combination with in-person care, it is necessary to develop best practice guidelines, including recommendations for optimal approaches for different clinical settings, clinical conditions, and patient populations^93^. Because telehealth has become an essential source for healthcare delivery over the last few years, even as COVID-19 care-induced changes have subsided, models that integrate telehealth and other types of virtual care with traditional in-person care processes will be essential, especially in settings where in-person access is limited due to distance or socioeconomic challenges. The successful integration of telehealth care has critical implications for the provision of care for patients with different acute and chronic conditions, in both ambulatory and in-patient care settings.

We found that the use of telehealth during COVID-19 in many, though not all, instances is comparable to in-person care across different clinical areas and different healthcare utilization, clinical, and process outcomes. Even as we transition through the COVID-19 era, telehealth continues to be one of the main modes of care delivery. Thus, models for integrating telehealth with traditional care processes become increasingly important, and ongoing evaluations of telehealth will be particularly valuable. Our findings suggest a direction for future work and can help inform policymakers, payors, and practitioners as they manage the use of telehealth during the remainder of the pandemic and beyond.

## Methods

This manuscript reports a subset of questions from a broader systematic review of telehealth during the COVID-19 era supported by the Agency for Healthcare Research and Quality (AHRQ)^96^.

### Data sources and searches

We searched PubMed, CINAHL, PsycINFO, and the Cochrane Central Register of Controlled Trials in April 2023, limiting the search to studies assessing telehealth care provided during the era of COVID-19 (March 2020–April 2023) (Search Terminology available in Supplementary Tables 1A–C). We removed the duplicate references and identified additional studies from reference lists and experts. An information specialist reviewed search strategies using the Peer Review of Electronic Search Strategies (PRESS) (PRESS) guidelines^97^. A Supplemental Evidence and Data for Systematic Review (SEADS) portal was posted in November 2021 and a Federal Register Notice was posted in October 2021 for the broader review.

### Study selection

Two team members independently screened citations against prespecified eligibility criteria at the abstract and full-text levels. At both levels, exclusion required that both screeners agree. Differences between reviewers regarding abstract or full-text eligibility were resolved through consensus. We included studies according to a “population, intervention, comparators, outcomes, timing, setting” (PICOT) framework. We included studies published in the English language of patients of any age (and their caregivers), all centers/locations of patient care, and healthcare providers of any type. We included only remotely delivered, synchronous medical services (e.g., telephone/audio, video visits) between a patient and a healthcare provider in an ambulatory setting or emergency department (ED) providing acute/urgent care, routine/chronic care, mental health services, wellness visits, post-hospital discharge care, and patient and specialist communications facilitated by an ED physician in an ED. We included studies comparing telehealth with in-person visits during the COVID-19 pandemic or comparing the use of telehealth in the COVID-19 era to services provided in the pre-COVID-19 era (assuming those services were predominantly in-person). We assessed the outcomes of telehealth versus in-person care including healthcare utilization, process, and clinical outcomes.

Because this topic was nominated by the AHRQ Learning Health System Panel, we focused on evidence relevant to the U.S. In addition to U.S.-based studies, we included all outpatient populations in countries with a population similar to that of the U.S., using the Organization for Economic Cooperation and Development (OECD) nations and excluding those with a World Health Organization classification below “upper income”^98^.

### Data extraction and risk of bias assessment

Two team members serially extracted data on study characteristics, population, intervention, and outcomes using data abstraction forms, after piloting and revising the forms on limited studies. They also independently assessed the risk of bias for the included studies using the Cochrane Risk of Bias Tool, Version 2, for randomized controlled trials (RCTs)^99^ and the Risk of Bias Assessment Tool for Non-Randomized Studies of Interventions (ROBINS-I) tool^100^ for non-randomized studies. Differences between the team members regarding the risk of bias were resolved through consensus. Study authors did not screen or conduct risk of bias assessments of their studies.

### Data synthesis and analysis

**Fig. 4:**
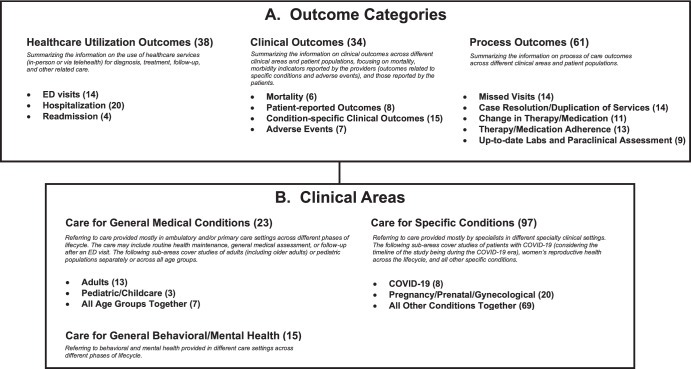
Organization of included studies in the review. The results of telehealth versus in-person comparison in the included studies are depicted by different outcome categories (**A**) and clinical areas (**B**). Numbers in parentheses present the number of studies for each outcome category and clinical area. The sum of studies across outcome categories and clinical areas exceeds 77 included studies. Some studies reported multiple outcome categories and/or clinical areas. COVID-19 coronavirus disease 2019, ED emergency department.

We identified 12 study outcomes and grouped these into three categories: healthcare utilization (3 subcategories), clinical (4 subcategories), and process (5 subcategories) outcomes (Fig. 4A). Because the outcomes of interest were reported across a very wide range of clinical conditions and areas, we also categorized the clinical areas into three groups (and seven sub-groups) and examined the 12 distinct outcomes across the clinical areas (Fig. 4B). Figure 4 provides a brief description of each outcome category and clinical area. We considered an effect or difference as clinically meaningful if it would result in changing clinical practice or care plan for the patient. We were unable to conduct a meta-analysis owing to limited and heterogeneous data for each clinical area/outcome comparison, missing information, and variation in the outcomes measured.

We rated the strength of evidence (SOE) for each outcome and clinical area by evaluating the study limitations, consistency of results, directness, and precision, using the grading scheme recommended in the AHRQ Methods Guide for Effectiveness and Comparative Effectiveness Reviews (Methods Guide)^101^. Two reviewers independently conducted the grading with input from other team members as needed to reach a consensus.

### Reporting summary

Further information on research design is available in the Nature Research Reporting Summary linked to this article.

### Supplementary information

Supplementary Information

Reporting Summary

## Data Availability

The datasets used and/or analyzed during the current study are available from the corresponding author upon reasonable request.

## Code availability

The codes developed and/or used during the current study are available from the corresponding author upon reasonable request.
