# Supplementary material for: Effectiveness of telehealth versus in-person care during the COVID-19 pandemic: a systematic review
Source: NPJ Digit Med. 2024 Jun 15;7:157. doi: 10.1038/s41746-024-01152-2 (PMC11180098; doi:10.1038/s41746-024-01152-2)
Supplement: Supplementary file 1 — Supplementary Information [file 41746_2024_1152_MOESM1_ESM.pdf]

Supplementary Information

Supplementary Table 1A-C. Search Strategy.

Supplementary Table 2A-C. Study, Participant, and Provider/ Health System Characteristics of Studies Investigating the Effectiveness of Telehealth vs. In-person Care During COVID-19.

Supplementary Table 3. Summary of Evidence for the Effects of Telehealth Versus In-person Care by Outcome Categories and Clinical Areas.

Supplementary Table 4A-C. Study Results Investigating the Effectiveness of Telehealth vs. In-person Care by Outcome Category and Clinical Area – Healthcare Utilization.

Supplementary Table 5A-D. Study Results Investigating the Effectiveness of Telehealth vs. In-person Care by Outcome Category and Clinical Area – Clinical Outcomes.

Supplementary Table 6A-E. Study Results Investigating the Effectiveness of Telehealth vs. In-person Care by Outcome Category and Clinical Area – Process Outcomes.

Supplementary Table 7A-B. Risk of Bias Assessment for Non-Randomized Studies and Randomized Clinical Trials.

Supplementary Table 8. Summary of Findings for Outcomes of Care Among Patients Receiving Telehealth Versus In-person Care During COVID-19.

Supplementary Figure 1A-C. The Difference in Health Outcomes for Patients with an Initial Telehealth versus In-person Visit by Outcome Category and Clinical Area.

Supplementary Table 1-A. PubMed search strategy.

#	String
1	“Virtual health”[tiab]
2	Telehealth[tiab]
3	Telemedicine[mh]
4	telemedicine[tiab]
5	“mobile health”[tiab]
6	mHealth[tiab]
7	“m-health”[tiab]
8	eHealth[tiab]
9	“e-health”[tiab]
9	“virtual care”[tiab]
10	1 OR 2OR 3 OR 4 OR 5 OR 6 OR 7 OR 8 OR 9 OR 9
11	“clinical study”[pt]
12	“clinical studies as topic”[mh]
13	“clinical study”[tiab]
14	“observational study”[pt]
15	“observational studies as topic”[mh]
16	“observational study”[tiab]
17	“clinical trial”[pt]
18	“clinical trials as topic”[mh]
19	“clinical trial”[tiab]
20	“comparative study”[pt]
21	“comparative study”[tiab]
22	“controlled clinical trial”[pt]
23	“controlled clinical trials as topic”[mh]
24	“controlled clinical trial”[tiab]
25	“randomized controlled trial”[pt]
26	“randomized controlled trials as topic”[mh]
27	“randomized controlled trial”[tiab] OR RCT[tiab] OR “randomised controlled trial”[tiab]
28	“cohort studies”[mh]
29	“cohort study”[tiab]
30	“retrospective studies”[mh]
31	“retrospective study”[tiab]
32	“cross-sectional studies”[mh]
33	“cross-sectional study”[tiab]
34	“qualitative research”[mh]
35	“evaluation study”[pt]
36	“evaluation studies as topic”[mh]
37	“focus groups”[mh]
38	interview[pt]
39	“interviews as topic”[mh]
40	“qualitative”[tiab]
41	“evaluation study”[tiab]
42	“focus group”[tiab]
43	Interview[tiab]
44	Interviews[tiab]

#	String
45	11 OR 12 OR 13 OR 14 OR 15 OR 16 OR 17 OR 18 OR 19 OR 20 OR 21 OR 22 OR 23 OR 24 OR 25 OR 26 OR 27 OR 28 OR 29 OR 30 OR 31 OR 32 OR 33 OR 34 OR 35 OR 36 OR 37 OR 38 OR 39 OR 40 OR 41 OR 42 OR 43 OR 44
46	Review[pt]
47	“systematic review”[pt]
48	"Meta-Analysis as Topic"[Mesh]
49	46 OR 47 OR 48
50	(10 AND 45) NOT 49

Supplementary Table 1-B. CINAHL and PsycINFO.

S15	(S11 AND S12) NOT S13	Limiters - Published Date: 20200301-20210731
S14	S11 AND S12	
S13	reviews OR "systematic review." OR metaanalysis" OR "meta analysis" OR "meta-analysis"	
S12	"clinical study" OR "observational study" OR "clinical trial" OR "comparative study" OR "controlled clinical Trial" OR "randomized controlled trial" OR "cohort study" OR "retrospective study" OR "cross-sectional study" OR "cross sectional study" OR "qualitative research" OR "evaluation study" OR "focus group" OR "focus groups" OR interview OR “randomised controlled trial”	
S11	S1 OR S2 OR S3 OR S4 OR S5 OR S6 OR S7 OR S8 OR S9 OR S10	
S10	(MM "Telehealth+")	
S9	MM "Telemedicine" OR MM "Online Therapy" OR MM "Teleconferencing" OR MM "Teleconsultation" OR MM "Telepsychiatry" OR MM "Telepsychology" OR MM "Telerehabilitation"	
S8	TI "e-Health" OR AB "e-Health"	
S7	TI eHealth OR AB eHealth	
S6	TI "m-Health" OR AB "m-Health"	
S5	TI mHealth OR AB mHealth	
S4	TI "mobile health" OR AB "mobile health"	
S3	TI telemedicine OR AB telemedicine	
S2	TI telehealth OR AB telehealth	
S1	TI "Virtual health" OR AB "Virtual health"	

Supplementary Table 1-C. Cochrane Central Register of Controlled Trials.

ID	Search
#1	("virtual health"):ti,ab,kw (Word variations have been searched)
#2	(telehealth):ti,ab,kw (Word variations have been searched)
#3	MeSH descriptor: [Telemedicine] explode all trees
#4	(telemedicine):ti,ab,kw (Word variations have been searched)
#5	("mobile health"):ti,ab,kw
#6	("m-health"):ti,ab,kw
#7	(mhealth):ti,ab,kw
#8	(ehealth):ti,ab,kw
#9	("e-health"):ti,ab,kw

ID	Search
#10	#1 or #2 or #3 or #4 or #5 or #6 or #7 or #8 or #9
* Date limited, no	

Supplementary Table 2-A. Characteristics of Studies Investigating the Effectiveness of Telehealth vs. In-person Care During COVID-19.

Author, year	Intervention	Study Design	Study Period	Country	Single or Multiple Sites	Geographic Location
Adams, 2023(1)	Not stated	Retrospective cohort	General COVID era	United States	Single Site	Not reported
Adepoju, 2022(2)	Not stated	Retrospective cohort	General COVID era	United States	Single Site	Not reported
Afonso Nogueira, 2021(3)	Telephone only	Prospective Cohort	Compares pre-COVID to COVID era	Portugal	Single Site	Not reported
Aiken, 2021(4)	Telephone plus video	Cross-sectional	Compares pre-COVID to COVID era	United Kingdom	Multiple site	Not reported
Arias, 2022(5)	Telephone plus video	Retrospective cohort	Compares pre-COVID to COVID era	United States	Single site	Urban
Barequet, 2021(6)	Telephone plus video	Retrospective cohort	General COVID era	Israel	Single site	Urban
Baughman, 2021(7)	Not stated	Retrospective cohort	Later COVID era (June 2020 and later)	United States	Not reported	Not reported
Borgen, 2021(8)	Telephone plus video	Prospective Cohort	Early COVID era (March-June 2020)	United States	Multiple site	Not reported
Boshara, 2022(9)	Not stated	Cross-sectional	General COVID era	United States	Multiple site	Urban
Bryson, 2023(10)	Not stated	Longitudinal cohort	General COVID era	United States	Multiple site	Urban
Carlberg, 2020(11)	Telephone plus video	Retrospective cohort	Early COVID era (March-June 2020)	United States	Multiple site	Not reported
Casariego-Vales, 2021(12)	Telephone plus video	Retrospective cohort	Later COVID era (June 2020 and later)	Spain	Multiple site	Not reported
Chen, 2023(13)	Telephone plus video	Cross-sectional	General COVID era	United States	Single site	Urban
Clark, 2022(14)	Telephone plus video	Retrospective cohort	Compares pre-COVID to post-COVID	Canada	Single site	Not reported
Cobo-Calvo, 2022(15)	Not stated	Retrospective cohort	Compares pre-COVID to COVID era	Spain	Single site	Urban
Cunningham, 2022(16)	Telephone plus video	Retrospective cohort	Compares pre-COVID to COVID era	United States	Multiple site	Urban
Cvietusa, 2022(17)	Telephone plus video	Retrospective cohort	Compares pre-COVID to COVID era	United States	Not reported	Not reported
D’Anna, 2021(18)	Telephone only	Retrospective cohort	Compares pre-COVID to COVID era	United Kingdom	Single site	Urban
Duryea, 2021(19)	Telephone only	Retrospective cohort	Compares pre-COVID to COVID era	United States	Single site	Not reported
Fortier, 2022(20)	Telephone plus video	Prospective cohort	Compares pre-COVID to COVID era	United States	Multiple site	Urban
Fredwall, 2021(21)	Telephone plus video	Prospective cohort	Early COVID era (March-June 2020)	United States	Single site	Not reported
Frost, 2022(22)	Telephone plus video	Cross-sectional	General COVID era	United States	Not reported	Not reported
Gaetani, 2021(23)	Telephone plus video	Prospective Cohort	Compares pre-COVID to COVID era	Italy	Single site	Not reported
Gainer, 2023(24)	Telephone plus video	Retrospective cohort	Later COVID era (June 2020 and later)	United States	Single site	Not reported

Author, year	Intervention	Study Design	Study Period	Country	Single or Multiple Sites	Geographic Location
Gao, 2022(25)	Not stated	Cross-sectional	Early COVID era (March-June 2020)	United States	Single site	Urban Suburban, Rural
Garmendia, 2021(26)	Telephone plus video	Prospective cohort	Compares pre-COVID to COVID era	Spain	Multiple site	Not reported
Griebeler, 2022(27)	Not stated	Randomized Clinical Trial	Later COVID era (June 2020 and later)	United States	Single site	Not reported
Hatef, 2022(28)	Telephone plus video	Prospective cohort	Compares pre-COVID to COVID era	United States	Claims data	Urban and rural
Hughes, 2022(29)	Not stated	Retrospective cohort	Early COVID era (March-June 2020)	United States	Single site	Not reported
Jazayeri, 2022(30)	Telephone plus video	Prospective cohort	General COVID era	United States	Single site	Not reported
Kablinger, 2022(31)	Telephone plus video	Retrospective cohort	Compares pre-COVID to COVID era	United States	Single site	Not reported
Kerestes, 2021(32)	Telephone plus video	Retrospective cohort	General COVID era	United States	Single site	Not reported
Khosla, 2022(33)	Telephone only	Retrospective cohort	Compares pre-COVID to COVID era	United States	Single site	Urban
Klain, 2021(34)	Telephone only	Retrospective cohort	Compares pre-COVID to COVID era	Italy	Single site	Urban
Kolb, 2021(35)	Telephone plus video	Retrospective cohort	Early COVID era (March-June 2020)	United States	Single site	Not reported
Korycinski, 2022(36)	Telephone only	Retrospective cohort	Early COVID era (March-June 2020)	United States	Single site	Not reported
Levinson, 2021(37)	Telephone plus video	Retrospective cohort	Compares pre-COVID to COVID era	United States	Not reported	Not reported
Li, 2021(38)	Telephone plus video	Retrospective cohort	Early COVID era (March-June 2020)	United Kingdom	Single site	Not reported
Lindhagen, 2022(39)	Not stated	Retrospective cohort	Compares pre-COVID to COVID era	Sweden	Single site	Urban
Liou, 2022(40)	Not stated	Retrospective cohort	Compares pre-COVID to COVID era	United States	Single site	Not reported
Liu, 2021(41)	Not stated	Prospective Cohort	Early COVID era (March-June 2020)	Australia	Single site	Urban
Mair, 2021(42)	Not stated	Retrospective cohort	Compares pre-COVID to COVID era	New Zealand	Single site	Urban
Mathews, 2022(43)	Not stated	Retrospective cohort	Compares pre-COVID to COVID era	United States	Single site	Not reported
McCoy, 2022(44)	Not stated	Cross-sectional	Compares pre-COVID to COVID era	United States	Single site	Urban
McNamara, 2021(45)	Not stated	Retrospective cohort	Compares pre-COVID to COVID era	United States	Multiple site	Urban
Metcalfe, 2023(46)	Telephone only	Retrospective cohort	Later COVID era (June 2020 and later)	United Kingdom	Single	Not reported
Minsky, 2021(47)	Telephone plus video	Prospective cohort	Compares pre-COVID to COVID era	Israel	Single site	Not reported
Mossack, 2022(48)	Not stated	Randomized Clinical Trial	Later COVID era (June 2020 and later)	United States	Single site	Not reported

Author, year	Intervention	Study Design	Study Period	Country	Single or Multiple Sites	Geographic Location
Offiah, 2022(49)	Telephone plus video	Retrospective cohort	Compares pre-COVID to COVID era	Ireland	Multiple site	Not reported
Ostberg, 2022(50)	Telephone plus video	Cross-sectional	General COVID era	United States	Multiple site	Suburban
Parise, 2021(51)	Telephone plus video	Prospective cohort	Early COVID era (March-June 2020)	Italy	Multiple site	Not reported
Phillips, 2021(52)	Not stated	Retrospective cohort	Early COVID era (March-June 2020)	United States	Single site	Not reported
Piga, 2022(53)	Telephone plus video	Prospective cohort	Early COVID era (March-June 2020)	Italy	Single site	Not reported
Pinsker, 2021(54)	Telephone plus video	Retrospective cohort	Early COVID era (March-June 2020)	United States	Single site	Not reported
Prato, 2022(55)	Telephone plus video	Randomized Clinical Trial	Later COVID era (June 2020 and later)	Italy	Single site	Not reported
Reddy, 2021(56)	Telephone plus video	Retrospective cohort	Early COVID era (March-June 2020)	United States	Single site	Not reported
Rene, 2022(57)	Not stated	Retrospective cohort	Early COVID era (March-June 2020)	United States	Single site	Not reported
Ripp, 2022(58)	Not stated	Retrospective cohort	Compares pre-COVID to COVID era	United States	Single site	Not reported
Rowe, 2021(59)	Telephone plus video	Retrospective cohort	General COVID era	Australia	Single site	Not reported
Ryskina, 2021(60)	Telephone plus video	Retrospective cohort	Early COVID era (March-June 2020)	United States	Multiple site	Urban and Suburban
Severino, 2022(61)	Telephone only	Prospective cohort	General COVID era	Italy	Single site	Not reported
Sevilis, 2022(62)	Telephone plus video	Retrospective cohort	Compares pre-COVID to COVID era	United States	Multiple site	Not reported
Shah, 2022(63)	Not stated	Retrospective cohort	General COVID era	United States	Single site	Urban
Sharma, 2020(64)	Telephone only	Retrospective cohort	Compares pre-COVID to COVID era	United Kingdom	Not reported	Not reported
Sohail, 2023(65)	Telephone plus video	Retrospective cohort	General COVID era	United States	Single site	Not reported
Sun, 2022(66)	Not stated	Retrospective cohort	General COVID era	United States	Single site	Urban
Szigety, 2022(67)	Not stated	Retrospective cohort	Compares pre-COVID to COVID era	United States	Single site	Not reported
Tarn, 2021(68)	Telephone plus video	Cross-sectional	Early COVID era (March-June 2020)	United States	Single site	Not reported
Tchang, 2022(69)	Telephone plus video	Retrospective cohort	Compares pre-COVID to COVID era	United States	Single site	Urban
Wabe, 2022(70)	Telephone plus video	Retrospective cohort	General COVID era	Australia	Multiple site	Urban, rural
Walker, 2023(71)	Not stated	Retrospective cohort	General COVID era	United States	Single site	Not reported
Watson, 2021(72)	Telephone only	Cross-sectional	Compares pre-COVID to COVID era	Australia	Single site	Not reported
Ye, 2022(73)	Telephone plus video	Retrospective cohort	General COVID era	United States	Single site	Urban

Author, year	Intervention	Study Design	Study Period	Country	Single or Multiple Sites	Geographic Location
Zayde, 2021(74)	Telephone plus video	Prospective cohort	Compares pre-COVID to COVID era	United States	Single site	Urban
Zhao, 2021(75)	Telephone plus video	Retrospective cohort	Compares pre-COVID to COVID era	United States	Single site	Not reported
Zhu, 2021(76)	Not stated	Retrospective cohort	Early COVID era (March-June 2020)	Australia	Multiple site	Urban
Zimmerman, 2021(77)	Telephone plus video	Prospective cohort	General COVID era	United States	Single site	Not reported

Supplementary Table 2-B. Participant Characteristics of Studies Investigating the Effectiveness of Telehealth vs. In-person Care During COVID-19.

Author, Year	Comparison Groups			N Patients	Patient Health Concern/Clinical Condition	Target Population	Age	Sex, n (%)
	Study Period	Arm/ Group*	Arm Label*					
Adams, 2023(1)	General COVID era	Full group	Overall	NR	Federally Qualified Health Centers	Socially/racially disadvantaged (all ages)	Mean: 27.1	Female: 51,906 (60.8)
Adepoju, 2022(2)	General COVID era	Full group	Overall	278,171	Federally Qualified Health Centers	Socially/racially disadvantaged (all ages)	Mean: 27.1	Female: 51,906 (60.8)
Afonso Nogueira, 2021(3)	Compares pre-COVID to COVID era	Arm 1	Individuals with heart failure attending outpatient cardiology appointments (Pre-COVID)	160	Cardiology	NR	Mean: NR	NR
		Full group	Individuals with heart failure attending outpatient cardiology appointments	196	Cardiology	NR	Mean: 71.4	Male: (68)
Aiken, 2021(4)	Compares pre-COVID to COVID era	Arm 1	Individuals receiving medical abortion following in-person visit	22,158	Pregnancy	Pregnant Women	Mean: 27.8	Female: (100)
		Arm 2	Individuals receiving medical abortion following telemedicine/hybrid visit	29,984	Pregnancy	Pregnant Women	Mean: 28.5	Female: (100)
Arias, 2022(5)	Compares pre-COVID to COVID era	Arm 1	Pre-telehealth implementation	780	Postpartum visits	Adults (18-65)	Median: 30.07	Female: 780 (100)
		Arm 2	Post-telehealth implementation	799	Postpartum visits	Adults (18-65)	Median: 30.35	Female: 799 (100)
Barequet, 2021(6)	General COVID era	NR	NR	NR	NR	NR	NR	NR
Baughman, 2021(7)	Later COVID era (June 2020 and later)	Full group	Overall	63,722	Diabetes	Adult and Elderly	Mean: 62	Female: 27667 (51.5)

Author, Year	Comparison Groups			N Patients	Patient Health Concern/Clinical Condition	Target Population	Age	Sex, n (%)
	Study Period	Arm/ Group*	Arm Label*					
Borgen, 2021(8)	Early COVID era (March-June 2020)	Arm 1	Individuals with new COVID-19 not receiving telehealth care management	593	COVID-19	Adult and Elderly (19-93)	Mean: 57.74	Male: (54)
		Arm 2	Individuals with new COVID-19 receiving telehealth care management	193	COVID-19	Adult and Elderly (19-93)	Mean: 57.44	Male: (61)
Boshara, 2022(9)	General COVID era	Full group	Overall	347	HIV	Adults (18-65)	Mean: 44.2	Male: 217 (62.5)
Bryson, 2023(10)	General COVID era	Full group	Patients receiving care in an adolescent medicine clinic	426	Adolescent medicine	Between 13-26 years old	Mean: 18.7	NR
Carlberg, 2020(11)	Early COVID era (March-June 2020)	Arm 1	Patients receiving telehealth and in-person evaluation in the ED	149	COVID-19	NR	Mean: 39.3	NR
		Arm 2	Patients receiving telehealth only evaluation in the ED	153	COVID-19	NR	Mean: 34.4	NR
Casariego-Vales, 2021(12)	Later COVID era (June 2020 and later)	Full group	Diagnosed with COVID in Galicia/ASLAM	4,384	COVID-19 Infection	Adults 18+	Mean: NR<br>Median: NR<br>Range: NR	NR
		Arm 1	Primary Care Monitoring	3,197	COVID-19 Infection	Adults 18+	Mean: NR<br>Median: NR<br>Range: NR	Female: (53.6)
		Arm 2	TELEA Telehealth Monitoring	1,187	COVID-19 Infection	Adults 18+	Mean: 65.6<br>Range: 15-99	Female: (53.4)
Chen, 2023(13)	General COVID era	Full group	Overall	NR	Primary care appointment	Adults 18+	NR	Female: (57.6)
Clark, 2022(14)	Compares pre-COVID to post-COVID	Full group	Overall	503	Gestational diabetes mellitus	NR	NR	NR
Cobo-Calvo, 2022(15)	Compares pre-COVID to COVID era	Full group	Full group	28,230 visits	Multiple sclerosis	Not reported	Mean: NR	NR
Cunningham, 2022(16)	Compares pre-COVID to COVID era	Arm 1	Pre-pandemic (in-person)	72	Opioid use disorder	Adult and Elderly	Mean: 45.4	Female: 23 (31.9)
		Arm 2	Pandemic (telemedicine)	35	Opioid use disorder	Adult and Elderly	Mean: 46.9	Female: 12 (34.3)
Cvietusa, 2022(17)	Compares pre-COVID to COVID era	Arm 1	No asthma care	2,977	Asthma	Adult and Elderly	Mean: NR	Female: 1730 (35.2)
		Arm 2	In-person only	1,792	Asthma	Adult and Elderly	Mean: NR	Female: 1139 (23.2)
		Arm 3	Mixed (in-person and virtual)	1,084	Asthma	Adult and Elderly	Mean: NR	Female: 758 (15.4)

Author, Year	Comparison Groups			N Patients	Patient Health Concern/Clinical Condition	Target Population	Age	Sex, n (%)
	Study Period	Arm/ Group*	Arm Label*					
		Arm 4	Virtual care only	1,952	Asthma	Adult and Elderly	Mean: NR	Female: 1293 (26.3)
D'Anna, 2021(18)	Compares pre-COVID to COVID era	Arm 1	In-person (2019)	180	Stroke patients	Adult and Elderly	Median: 68.5	Male: 121 (67.22)
		Arm 2	Telephone (2020)	136	Stroke patients	Adult and Elderly	Median: 65	Male: 99 (72.79)
Duryea, 2021(19)	Compares pre-COVID to COVID era	Arm 1	Pregnant individuals who received in-person pre-natal care	6,559	Pregnancy	Pregnant Women	Mean: 27.8	Female: (100)
		Arm 2	Pregnant individuals who received audio telehealth and in-person prenatal care	6,048	Pregnancy	Pregnant Women	Mean: 27.7	Female: (100)
Fortier, 2022(20)	Compares pre-COVID to COVID era	Arm 1	in-person (usual care)	29	U.S. Veterans mental health treatment	Adults (18-65)	Mean: 39.9	Male: 25 (86.2)
		Arm 2	Telehealth (virtual care)	45	U.S. Veterans mental health treatment	Adults (18-65)	Mean: 41.8	Male: 36 (80.0)
Fredwall, 2021(21)	Early COVID era (March-June 2020)	Arm 1	Patients seen in-person at Epilepsy Clinic	101	Epilepsy	Children (<18)	Mean: NR<br>Median: NR<br>Range: NR	NR
		Arm 2	Patients seen by telemedicine at Epilepsy Clinic	23	Epilepsy	Children (<18)	Mean: 14	Female: (65)
Frost, 2022(22)	General COVID era	Full group	Overall	17,175	Behavioral Health – Opioid use disorder	18 and above	Mean: NR	Male: 15,835 (92.2%)
Gaetani, 2021(23)	Compares pre-COVID to COVID era	Arm 1	Pre-COVID: Individuals with Hereditary Hemorrhagic Telangiectasia	45	HHT	NR	Mean: 56.7	Female: (53)
		Arm 2	COVID era: Individuals with Hereditary Hemorrhagic Telangiectasia (receiving telehealth)	45	HHT	NR	Mean: 56.7	Female: (53)
Gainer, 2023(24)	Later COVID era (June 2020 and later)	Full group	Overall	544	Substance use disorder – Behavioral health	18 and above	Mean: NR	Female: 247 (45.4%)
Gao, 2022(25)	Early COVID era (March-June 2020)	Full group	Pregnant women receiving care	1876	Pregnancy	All age groups	Mean: NR	NR
Garmendia, 2021(26)	Compares pre-COVID to COVID era	Arm 1	Individuals with Sleep Apnea attending outpatient clinic, pre-covid/in-person	193	Sleep Apnea	NR	Mean: NR	NR

Author, Year	Comparison Groups			N Patients	Patient Health Concern/Clinical Condition	Target Population	Age	Sex, n (%)
	Study Period	Arm/ Group*	Arm Label*					
		Arm 2	Individuals with Sleep Apnea attending outpatient clinic, COVID era, telehealth	77	Sleep Apnea	NR	Mean: 56	Male: 75
Griebeler, 2022(27)	Later COVID era (June 2020 and later)	Full group	Overall	70	Overweight/obesity	Adults (18-65)	Mean: 42.2	Female: 63 (90%)
Hatef, 2022(28)	Compares pre-COVID to COVID era	Full group	Overall	40,739,915	Blue Health Blue Shield Claims data	All age groups	Range: 0-50+	Female: 20480768 (50.3)
Hughes, 2022(29)	Early COVID era (March-June 2020)	Full group	Overall	NR	Acute decompensated heart failure (follow up after hospitalization)	18 and above	Mean: NR	Male: 87
Jazayeri, 2022(30)	General COVID era	Full group	Overall	1,769	Patients with abdominal pain in gastroenterology clinic	Children (0-22)	Mean: 11.6	Male: 671 (59.3%)
Kablinger, 2022(31)	Compares pre-COVID to COVID era	Full group	Full group	2145	Psychiatric patients	Adult and Elderly	Range: 18-87	Female: 1485 (69.23)
Kerestes, 2021(32)	General COVID era	Arm 1	Patients who received abortion services in-person	110	Family Planning	Pregnancy	Mean: 28.1	Female: (100)
		Arm 2	Patients who received abortion services via telehealth	224	Family Planning	Pregnancy	Mean: 27.3	Female: (100)
Khosla, 2022(33)	Compares pre-COVID to COVID era	Arm 1	Pre-pandemic	215	Hypertensive disorder of pregnancy	Adults (18-65)	Mean: 29	Female: 215 (100)
		Arm 2	Post-pandemic	258	Hypertensive disorder of pregnancy	Adults (18-65)	Mean: 30	Female: 258 (100)
Klain, 2021(34)	Compares pre-COVID to COVID era	Arm 1	2019 visit (corresponding period to first visit during COVID-19)	75	Differentiated thyroid cancer	Adults (18-65)	Mean: 46	Male: 21 (28)
		Arm 2	2020 first telemedicine visit during COVID-19	54	Differentiated thyroid cancer	Adults (18-65)	Mean: 45	Male: 13 (24)
		Arm 3	2019 visit (corresponding period to follow-up visit during COVID-19)	450	Differentiated thyroid cancer	Adults (18-65)	Mean: 50	Male: 83 (18)
		Arm 4	2020 telemedicine follow-up visit during COVID-19	391	Differentiated thyroid cancer	Adults (18-65)	Mean: 51	Male: 91 (23)

Author, Year	Comparison Groups			N Patients	Patient Health Concern/Clinical Condition	Target Population	Age	Sex, n (%)
	Study Period	Arm/ Group*	Arm Label*					
Kolb, 2021(35)	Early COVID era (March-June 2020)	Arm 1	Patients receiving in-person outpatient care at ENT clinic (Recurrent Acute Otitis Media Cohort)	50	Otolaryngology	Children (<18)	Mean: 24.4 (months)	Male: (62)
		Arm 2	Patients receiving telehealth outpatient care at ENT clinic, (Recurrent Acute Otitis Media Cohort)	50	Otolaryngology	Children (<18)	Mean: 17 (months)	Male: (60)
		Arm 1	Patients receiving in-person outpatient care at ENT clinic, (Sleep-Disorder Breathing Cohort)	64	Otolaryngology	Children (<18)	Mean: 5.29 (years)	Male: (55)
		Arm 2	Patients receiving telehealth outpatient care at ENT clinic, (Sleep-Disorder Breathing Cohort)	64	Otolaryngology	Children (<18)	Mean: 5.47 (years)	Male: (50)
Korycinski, 2022(36)	Early COVID era (March-June 2020)	Full group	Full group	293	COVID-19	Adult and Elderly	Mean: 46.03	Female: 163 (55.6)
Levinson, 2021(37)	Compares pre-COVID to COVID era	Arm 1	In-person	60	Eating disorder	Adults (18-65)	Mean: 25.07	Cisgender women: 51 (85)
		Arm 2	Telehealth (Zoom)	33	Eating disorder	Adults (18-65)	Mean: 24.52	Cisgender women: 29 (87.88)
Li, 2021(38)	Early COVID era (March-June 2020)	Arm 1	Patients triaged in-person for ophthalmologic issue	451	Ophthalmology	NR	Median: 49	Male: (55)
		Arm 2	Patients receiving virtual triage for ophthalmologic issue	404	Ophthalmology	NR	Median: 43	Female: (54)
Lindhagen, 2022(39)	Compares pre-COVID to COVID era	Full group	Full group	894	Inflammatory bowel disease	Adults (18-65)	Mean: 47.6	Female: 422 (47.2)
Liou, 2022(40)	Compares pre-COVID to COVID era	Full group	Patients undergoing eye movement desensitization and reprocessing	Baseline: 267 Follow-up: 232	Mental health	18 and above	Mean: 51.4	Female: 203 (70.5)
Liu, 2021(41)	Early COVID era (March-June 2020)	Full group	Full Group clinic patients at various outpatient facilities	1,376	Gastroenterology/Rheumatology	NR	Mean: 55.06	Female: (52.3)
		Arm 1	Pre-COVID clinic patients at various outpatient facilities	692	Gastroenterology/Rheumatology	NR	Mean: 55.63	Female: (53.6)

Author, Year	Comparison Groups			N Patients	Patient Health Concern/Clinical Condition	Target Population	Age	Sex, n (%)
	Study Period	Arm/ Group*	Arm Label*					
		Arm 2	COVID era clinic patients at various outpatient facilities	684	Gastroenterology/Rheumatology	NR	Mean: 54.47	Female: (51)
Mair, 2021(42)	Compares pre-COVID to COVID era	Arm 1	Pre-COVID (2019)	210	Rheumatology patients	NR	Mean: 54.6	Female: 142 (67.6)
		Arm 2	Telehealth	340	Rheumatology patients	NR	Mean: 55.6	Female: 244 (71.8)
Mathews, 2022(43)	Compares pre-COVID to COVID era	Full group	NR	NR	Pain management by nurse practitioners	Adults (22-90)	NR	NR
McCoy, 2022(44)	Compares pre-COVID to COVID era	Arm 1	In-person before telemedicine	113	Pediatric otolaryngology patients	Children (<18)	Mean: 4.99	Female: 43 (38.1)
		Arm 2	Telemedicine	59	Pediatric otolaryngology patients	Children (<18)	Mean: 6.15	Female: 30 (50.8)
		Arm 3	In-person during telemedicine	4	Pediatric otolaryngology patients	Children (<18)	Mean: 8.78	Female: 3 (75)
McNamara, 2021(45)	Compares pre-COVID to COVID era	Full group	Full group	537	Primary care, pharmacy visit	Adult and Elderly	Mean: 62.52	Female: 273 (50.8)
Metcalfe, 2023(46)	Later COVID era (June 2020 and later)	Full group	Overall	150	Otology referral	18 and above	Mean: 55	Female: 71 (47)
Minsky, 2021(47)	Compares pre-COVID to COVID era	Full group	Overall	279	Overweight	NR	Mean: 53	Female: (69)
Mossack, 2022(48)	Later COVID era (June 2020 and later)	Full group	Overall	48	Overactive bladder	18 and above	Mean: 51	Female: (100)
Offiah, 2022(49)	Compares pre-COVID to COVID era	Arm 1	Traditional clinic	1,220	Cardiology patients	Adult and Elderly	Mean: 61	Female: 548 (44.9)
		Arm 2	Virtual clinic	496	Cardiology patients	Adult and Elderly	Mean: 60	Female: 208 (41.9)
Ostberg, 2022(50)	General COVID era	Arm 2	Telehealth (Zoom)	455	Patients with chest pain	Adults (18-65)	Median: 44	Male: 228 (50.1)
Parise, 2021(51)	Early COVID era (March-June 2020)	Arm 1	Not included in telemedicine study	43	Type-1 diabetes	Adults (18-65)	Mean: 37	Male: 21 (49)
		Arm 2	Included in telemedicine study	166	Type-1 diabetes	Adults (18-65)	Mean: 40	Male: 80 (48.2)

Author, Year	Comparison Groups			N Patients	Patient Health Concern/Clinical Condition	Target Population	Age	Sex, n (%)
	Study Period	Arm/ Group*	Arm Label*					
Phillips, 2021(52)	Early COVID era (March-June 2020)	Arm 1	Individuals presenting to initial in-person visit at respiratory assessment center	741	Pulmonary	NR	Median: 43.5	Female: (63.6)
		Arm 2	Individuals presenting to initial telehealth visit at respiratory assessment center	564	Pulmonary	NR	Median: 42.05	Female: (66.8)
Piga, 2022(53)	Early COVID era (March-June 2020)	Full group	Patients with inflammatory rheumatologic diseases	106	Rheumatology	NR	Median: 45.4	Male: 47 (44.3)
Pinsker, 2021(54)	Early COVID era (March-June 2020)	Arm 1	Individuals with diabetes receiving in-person insulin pump training	NR	Diabetes	Other (define)	Mean: NR<br>Median: NR<br>Range: NR	NR
		Arm 2	Individuals with diabetes receiving virtual insulin pump training	8,984	Diabetes	Other (define)	Mean: NR<br>Median: NR<br>Range: NR	NR
Prato, 2022(55)	Later COVID era (June 2020 and later)	Full group	Overall	40	Tourette syndrome	Children (<18)	Mean: 13.5	Male: 36 (90)
Reddy, 2021(56)	Early COVID era (March-June 2020)	Full group	Overall	1,744	Cancer patients	Adult and Elderly	Median: 60	Female: 924 (53)
Rene, 2022(57)	Early COVID era (March-June 2020)	Full group	Overall	338	Depression/Anxiety	18 and above	Mean: 50.6	Male: 45 (28.7)
Ripp, 2022(58)	Compares pre-COVID to COVID era	Arm 1	In-person (2019)	1,077	Developmental-behavioral pediatrics	Children (<18)	Mean: 9.4	Female: 270 (25.1)
		Arm 2	Telehealth (2020)	354	Developmental-behavioral pediatrics	Children (<18)	Mean: 9.3	Female: 91 (25.7)
Rowe, 2021(59)	Early COVID era (March-June 2020)	Arm 1	Telephone Cardiology Outpatient Visits	1,118	Cardiology	NR	Median: 67<br>Range: 54-76	Male: (56.4)
		Arm 2	Video Cardiology Outpatient Visits	327	Cardiology	NR	Median: 61<br>Range: 46-71	Male: (65.1)
Ryskina, 2021(60)	Compares pre-COVID to COVID era<br>General COVID era	Arm 1	Patients having in-person primary care appointment	6,792	Primary Care	Elders (65+)	Mean: 74.8	Female: (60.8)
		Arm 2	Patients having telehealth primary care appointment	10,311	Primary Care	Elders (65+)	Mean: 75.1	Female: (60.5)
Severino, 2022(61)	General COVID era	Full group	Overall	184	Heart failure patients	18 and above	Mean:70.5	Female: 31 (34)
Sevilis, 2022(62)	Early COVID era (March-June 2020)	Arm 1	Pre-COVID	15,226	Stroke patients	Adult and Elderly	Mean: 67	Female: 8082 (53.1)
		Arm 2	COVID	11,105	Stroke patients	Adult and Elderly	Mean: 66.7	Female: 5802 (52.2)

Author, Year	Comparison Groups			N Patients	Patient Health Concern/Clinical Condition	Target Population	Age	Sex, n (%)
	Study Period	Arm/ Group*	Arm Label*					
Shah, 2022(63)	General COVID era	Full group	Overall	16,987	Patients with ED visits	Adult (18-65)	Mean: 53	NR
Sharma, 2020(64)	Compares pre-COVID to early COVID era (March-June 2020)	Arm 1	IBD Services before COVID (2019)	1,036	Irritable Bowel Disease (IBD)	NR	Median: 36 Range: 22-76	Female: (46)
		Arm 2	IBD Services after COVID (2020)	334	Irritable Bowel Disease (IBD)	NR	Median: 29 Range: 17-91	Female: (36)
Sohail, 2023(65)	General COVID era	Full group	Overall	NR	HIV primary care appointment	NR	Mean: NR	Male: (69.7)
Sun, 2022(66)	General COVID era	Full group	Overall	NR	Adults with diabetes	18 and above	Mean: NR	Female: 703 (49.37)
Szigety, 2022(67)	Compares pre-COVID to early COVID era (March-June 2020)	Full group	Full group	4,327	Genetic conditions	Children (<18)	Mean: 87.9	Female: 2258 (46.2)
Tarn, 2021(68)	Compares pre-COVID to early COVID era (March-June 2020)	Arm 1	Patients receiving in-person outpatient care at primary care clinic	52	Primary Care	NR	Mean: 26.4 Range: 1-72	Female: (42.3)
		Arm 2	Patients receiving telephone outpatient care at primary care clinic	55	Primary Care	NR	Mean: 40.7 Range: 1-89	Female: (70.9)
		Arm 3	Patients receiving telehealth(video) outpatient care at primary care clinic	89	Primary Care	NR	Mean: 40.3 Range: 4-73	Female: (64)
Tchang, 2022(69)	Compares pre-COVID to COVID era	Arm 1	In-person	69	Overweight/Obese patients	Adults (18-65)	Median: 56	Female: 50 (72)
		Arm 2	Hybrid	85	Overweight/Obese patients	Adults (18-65)	Median: 49	Female: 64 (75)
		Arm 3	Video	91	Overweight/Obese patients	Adults (18-65)	Median: 49	Female: 71 (78)
Wabe, 2022(70)	Compares pre-COVID to COVID era	Arm 1	Face to face	8,303,233	General practice	Adult and Elderly	Mean: NR	Female: 4684918 (56.4)
		Arm 2	Telehealth	5,304,983	General practice	Adult and Elderly	Mean: NR	Female: 3324348 (62.7)
Walker, 2023(71)	General COVID era	Full group	Overall	31,654	NR	NR	Mean: NR	NR
Watson, 2021(72)	General COVID era	Arm 1	Pre-telephone clinic (face to face)	814	Cancer patients	Adult and Elderly	Mean: 62.52	NR

Author, Year	Comparison Groups			N Patients	Patient Health Concern/Clinical Condition	Target Population	Age	Sex, n (%)
	Study Period	Arm/ Group*	Arm Label*					
		Arm 2	Post-introduction (telephone)	910	Cancer patients	Adult and Elderly	Mean: 62.77	NR
Ye, 2022(73)	General COVID era	Arm 1	In-person	20,745	High blood pressure	Adult and Elderly	Mean: 66.7	Female: 10256 (49.4)
		Arm 2	1 telemedicine visit	6,878	High blood pressure	Adult and Elderly	Mean: 65.7	Female: 3553 (51.7)
		Arm 3	2 or more telemedicine visits	5,104	High blood pressure	Adult and Elderly	Mean: 65.4	Female: 3236 (63.4)
Zayde, 2021(74)	Compares pre-COVID to COVID era	Full group	Full group	12 dyads	Caregivers and children (general mental health)	Children and adult caregivers	Mean:<br>Caregiver age: 44.17<br>Child age: 11.08	Female (for children): 3 (25)
Zhao, 2021(75)	Compares pre-COVID to COVID era	Arm 1	Individuals with heart failure attending in-person outpatient appointments	39	Heart Failure	NR	Mean: 71	Female: (25.6)
		Arm 2	Individuals with heart failure attending telehealth appointments	43	Heart Failure	NR	Mean: 70.4	Female: (27.9)
Zhu, 2021(76)	Early COVID era (March-June 2020)	Arm 1	2019 cohort (face to face)	1,443	Rheumatology patients	Adults (18-65)	Median: 55	Female: 902 (70.1)
		Arm 2	2020 cohort (telehealth)	1,597	Rheumatology patients	Adults (18-65)	Median: 54	Female: 1042 (69.8)
Zimmerman, 2021(77)	Early COVID era (March-June 2020)	Arm 1	In-person	207	General	Adults (18-65)	Mean: 38.16	Male: 62 (30)
		Arm 2	Telehealth	207	General	Adults (18-65)	Mean: 35.88	Male: 55 (26.6)

ED=emergency department; ENT= ear, nose, and throat; F2F=face to face; N=sample size; NR=not reported; RIC=referred to clinic

* Study Arm/Group is defined in the column called Arm Label. Comparisons include in-person (arm 1) vs. telehealth (arm 2) visits and services provided in pre-COVID-19 era (assuming those services were predominantly in-person, arm 1) to telehealth in the COVID-19 era (arm 2). Some studies reported participants characteristics only for full population, others reported them for comparison groups.

Supplementary Table 2-C. Provider/ Health System Characteristics of Studies Investigating the Effectiveness of Telehealth vs. In-person Care During COVID-19.

Author, year	Healthcare System	Specialty/Clinical Focus/Health System Setting
Adams, 2023(1)	Large/Regionally representative	Federally Qualified Health Centers
Adepoju, 2022(2)	Large/Regionally representative	Federally Qualified Health Centers
Afonso Nogueira, 2021(3)	Representative of a single large facility or organization	Heart failure clinic in hospital
Aiken, 2021(4)	NR	Abortion providers
Arias, 2022(5)	Representative of a single large facility or organization	Postpartum care
Barequet, 2021(6)	NR	NR
Baughman, 2021(7)	Large/Regionally representative	Primary care (wide, full-range care)
Borgen, 2021(8)	NR	Home-care services
Boshara, 2022(9)	Large/Regionally representative	HIV

Author, year	Healthcare System	Specialty/Clinical Focus/Health System Setting
Bryson, 2023(10)	Limited study, small facility	Adolescent medicine
Carlberg, 2020(11)	Limited study, small facility	Tertiary care, Level I trauma center
Casariego-Vales, 2021(12)	Regional healthcare network	At home monitoring
Chen, 2023(13)	Large/Regionally representative	Primary care (wide, full-range care)
Clark, 2022(14)	Limited study, small facility	Pregnancy clinic
Cobo-Calvo, 2022(15)	Limited study, small facility	MS/Autoimmune disorder center
Cunningham, 2022(16)	Representative of a single large facility or organization	Rehab/PT/OT/etc.
Cvietusa, 2022(17)	Large/Regionally representative	Primary care (wide, full-range care)
D'Anna, 2021(18)	Representative of a single large facility or organization	Primary care (wide, full-range care)
Duryea, 2021(19)	NR	Antenatal and postpartum care clinic
Fortier, 2022(20)	Large/Regionally representative	Mental health
Fredwall, 2021(21)	NR	Epilepsy clinic
Frost, 2022(22)	Nationally representative	Behavioral health
Gaetani, 2021(23)	NR	Hereditary hemorrhagic telangiectasia center
Gainer, 2023(24)	Limited study, small facility	Behavioral health
Gao, 2022(25)	Large/Regionally representative	OBGYN
Garmendia, 2021(26)	NR	Sleep unit
Griebeler, 2022(27)	Large/Regionally representative	Endocrinology and Metabolism Institute
Hatef, 2022(28)	Nationally representative	Not reported
Hughes, 2022(29)	Limited study, small facility	Acute decompensated heart failure
Jazayeri, 2022(30)	Limited study, small facility	Gastroenterology clinic
Kablinger, 2022(31)	Limited study, small facility	Mental health
Kerestes, 2021(32)	NR	Family planning clinic
Khosla, 2022(33)	Limited study, small facility	Primary care (wide, full-range care)
Klain, 2021(34)	NR	Radiometabolic Unit
Kolb, 2021(35)	NR	NR
Korycinski, 2022(36)	Limited study, small facility	Primary care (wide, full-range care)
Levinson, 2021(37)	Limited study, small facility	Eating disorder
Li, 2021(38)	NR	Eye hospital
Lindhagen, 2022(39)	Representative of a single large facility or organization	Gastroenterology department
Liou, 2022(40)	Limited study, small facility	Mental health
Liu, 2021(41)	NR	NR
Mair, 2021(42)	NR	Rheumatology clinic

Author, year	Healthcare System	Specialty/Clinical Focus/Health System Setting
Mathews, 2022(43)	Representative of a single large facility or organization	Pain management in the context of general medical care
McCoy, 2022(44)	Representative of a single large facility or organization	Otolaryngology
McNamara, 2021(45)	Representative of a single large facility or organization	Pharmacy visit
Metcalfe, 2023(46)	Limited study, small facility	Otorhinolaryngology
Minsky, 2021(47)	NR	Weight management clinic
Mossack, 2022(48)	Limited study, small facility	Urology
Offiah, 2022(49)	Large/Regionally representative	General cardiology clinics in hospital
Ostberg, 2022(50)	NR	Primary care (wide, full-range care)
Parise, 2021(51)	Representative of a single large facility or organization	Diabetes care center
Phillips, 2021(52)	NR	Primary care run respiratory assessment center
Piga, 2022(53)	Representative of a single large facility or organization	Rheumatology clinic
Pinsker, 2021(54)	NR	Diabetic center
Prato, 2022(55)	Limited study, small facility	Pediatric neurology clinic
Reddy, 2021(56)	Limited study, small facility	Cancer care
Rene, 2022(57)	Large/Regionally representative	Primary care (wide, full-range care)
Ripp, 2022(58)	Representative of a single large facility or organization	Mental health
Rowe, 2021(59)	NR	Cardiology clinic
Ryskina, 2021(60)	Regional healthcare network	Primary care clinic
Severino, 2022(61)	Limited study, small facility	NR
Sevilis, 2022(62)	Nationally representative	Stroke care
Shah, 2022(63)	Large/Regionally representative	ED Visits
Sharma, 2020(64)	NR	NR
Sohail, 2023(65)	Large/Regionally representative	Primary care (wide, full-range care)
Sun, 2022(66)	Representative of a single large facility or organization	Primary care (wide, full-range care)
Szigety, 2022(67)	Limited study, small facility	Clinical genetics
Tarn, 2021(68)	NR	NR
Tchang, 2022(69)	Limited study, small facility	Wellness/Health education
Wabe, 2022(70)	Large/Regionally representative	Primary care (wide, full-range care)
Walker, 2023(71)	Nationally representative	NR
Watson, 2021(72)	Representative of a single large facility or organization	Cancer care
Ye, 2022(73)	Representative of a single large facility or organization	Primary care (wide, full-range care)
Zayde, 2021(74)	Limited study, small facility	Mental health
Zhao, 2021(75)	Representative of a single large facility or organization	Multidisciplinary clinic in hospital

Author, year	Healthcare System	Specialty/Clinical Focus/Health System Setting
Zhu, 2021(76)	Representative of a single large facility or organization	Rheumatology
Zimmerman, 2021(77)	Representative of a single large facility or organization	Hospital
ED= Emergency Department; N=sample size; NR=not reported		

Supplementary Table 3. Summary of Evidence for the Effects of Telehealth Versus In-person Care by Outcome Categories and Clinical Areas.*

Outcome Categories		Clinical Areas						
		**Care for General Medical Conditions, Adults**	**Care for General Medical Conditions, Child**	**Care for General Medical Conditions, All Ages**	**Care for Specific Conditions, COVID-19**	**Care for Specific Conditions, Pregnancy/ Prenatal/ Gynecological care**	**Care for Specific Conditions, Other Conditions**	**Care for General Behavioral/ Mental Health**
**Healthcare utilization (n=38 studies)[b]**	Emergency Department visits	Low P (1)	Low = (1)	Moderate P/T (2)	Low P (3)	Low P (1)	Low T (6)	No evidence
	Hospitalization	Moderate = (3)	No evidence	Moderate P/T (2)	Low P (2)	Low P (2)	Low T (11)	No evidence
	Readmission	No evidence	No evidence	No evidence	Low = (2)	Low = (1)	Low T (1)	No evidence
**Clinical outcomes (n=34 studies)[b]**	Mortality	No evidence	No evidence	No evidence	No evidence	Low = (2)	Low T (4)	No evidence
	Patient-reported outcomes	No evidence	No evidence	No evidence	No evidence	Low P (2)	Low T (1)	Low T (5)
	Condition-specific clinical outcomes	Low = (1)	No evidence	No evidence	No evidence	Low P (4)	Low P (8)	Low T (2)
	Adverse events	Low T (1)	No evidence	No evidence	No evidence	Low T (3)	Low T (2)	Low = (1)
**Process outcomes (n=61 studies)[†]**	Missed visits	Low P (2)	No evidence	Low T (1)	No evidence	Low T (3)	Low T (6)	Low P (2)
	Case resolution/ Duplication of services	No evidence	Low = (1)	Moderate P/T (2)	Insufficient ?\ (1)	Insufficient ?\ (1)	Low T (9)	No evidence
	Change in therapy/ Medication	Moderate P (2)	No evidence	No evidence	No evidence	No evidence	Low P (9)	No evidence
	Therapy/ Medication adherence	Low T (3)	No evidence	No evidence	No evidence	No evidence	Low P (5)	Low T (5)
	Up-to-date labs and paraclinical assessment	No evidence	Low P (1)	No evidence	No evidence	Low = (1)	Low P (7)	No evidence

Insufficient, orange: insufficient evidence; **Moderate, green**: moderate strength of evidence; **Low, blue**: low strength of evidence.

* Intervention T: favors telehealth; P: favors in-person; P/T: acute care patients favor in-person and chronic care patients favor telehealth; = little to no difference between in-person vs. telehealth; ?\: insufficient evidence to make a conclusion. Numbers in parentheses present the number of studies for each cell.

† The total is more than 63 included studies as some studies reported multiple outcomes.

Supplementary Table 4-A. Study Results Investigating the Effectiveness of Telehealth vs. In-person Care by Outcome Category and Clinical Area – Healthcare Utilization, Emergency Department Visits.

Author, Year	Clinical Area	Arm Definition (n)	Outcome	Time of Analysis	Participants With Outcomes, n(%)	Comparison	Adjusted
Shah, 2022(63)	1a	Arm 1 – In-person ED visits (11,818)<br>Arm 2 – Telehealth ED visits (5,169)	ED return visits	30 days	Arm 1 – Events: 1,865 (0.16)<br>Arm 2 – Events: 937 (0.18)	Ref: Arm 1<br>Odds ratio: 1.23 (95% CI: 1.09 to 1.39), p=0.001	Sociodemographic and clinical factors, time from ED discharge to follow-up
Jazayeri, 2022(30)	1b	Arm 1 – In-person (1,131)<br>Arm 2 – Telehealth (638)	ED visits	3 months	NR (NR)	Ref: Arm 1<br>t-test between means: Arm 1: 0.037 (SD 0.246)<br>t-test between means: Arm 2: 0.017 (SD 0.142), p=0.61	No
Hatef, 2022(28)	1c	Arm 1 – First encounter in- person (493,716)<br>Arm 2 – First encounter telemedicine (113,857)	Acute ambulatory care, ED follow-up	14 days	NR (NR)	Ref: Arm 1<br>Odds ratio: 1.11 (95% CI: 1.06 to 1.16), p=NR	Type of acute and chronic ambulatory care sensitive conditions treated during the episode.
		Arm 1 – First encounter in- person (410,743)<br>Arm 2 – First encounter telemedicine (94,481)	Chronic ambulatory care, ED follow-up	14 days	NR (NR)	Ref: Arm 1<br>Odds ratio: 0.96 (95% CI: 0.92 to 1.01), p=NR	Type of acute and chronic ambulatory care sensitive conditions treated during the21pisodee.
Phillips, 2021(52)	1c	Arm 1 – Individuals presenting to initial in-person visit at Respiratory Assessment center (741)<br>Arm 2 – Individuals presenting to initial telehealth visit at Respiratory Assessment Center (564)	ED visit	14 Days	Arm 1 – Events: 29 (3.9)<br>Arm 2 – Events: 28 (5)	Ref: Arm 1<br>Chi-squared: NR, p=0.357	No
Borgen, 2021(8)	2a	Arm 1 – Individuals with new COVID-19 not receiving telehealth care management discharged from the hospital (593)<br>Arm 2 – Individuals with new COVID-19 receiving telehealth care management (192)	Hospital re-encounters (ED or Observation Unit)	30 days	Arm 1 – 167 (28.2)<br>Arm 2 – 24 (12.5)	Ref: Arm 1,<br>Chi-squared test of independence: 19.3, p≤0.001	No
Casariego-Vales, 2021(12)	2a	Arm 1 – Primary Care Monitoring (3197)<br>Arm 2 – TELEA Telehealth Monitoring (1187)	ED follow-up within study period	68 days	Arm 1 – Events: 227 (7.1)<br>Arm 2 – Events: 307 (25.9)	Ref: Arm 1,<br>Assumed t-test/man Whitney: NR, p≤.0001	No
Korycinski, 2022 (36)	2a	Arm 1 – Control (154)<br>Arm 2 – Telephone based intervention (139)	ED visit	30 days	Arm 1 – 24 (15.6)<br>Arm 2 – 13 (10.1)	Ref: Arm 1,<br>p=0.117	No
Kerestes, 2021(32)	2b	Arm 1 – In person (94)<br>Arm 2 – Telemedicine + med pick up (124)<br>Arm 2 – Telemedicine + Mailed Medicine (69)	ED visit	252 days	Arm 1 – 2 (2.1)<br>Arm 2 – 5 (4)<br>Arm 3 – 4 (5.8)	NR	No

Author, Year	Clinical Area	Arm Definition (n)	Outcome	Time of Analysis	Participants With Outcomes, n(%)	Comparison	Adjusted
Afonso Nogueira, 2021(3)	2c	Arm 1 – Individuals with Heart Failure attending outpatient cardiology appointments (Pre-COVID) (160)<br>Arm 2 – Individuals with Heart Failure attending outpatient cardiology appointments (43)	ED visits for heart failure	497 days	Arm 1 -214 (NR)<br>Arm 2 – 52 (NR)	Ref: Arm 1, Chi-squared: NR, p=0.27	No
Cvietusa, 2022(17)	2c	Arm 1 – No asthma care (Baseline: NR, Follow-up: 2,977)<br>Arm 2 – In-person only (Baseline: NR, Follow-up: 1,792)<br>Arm 3 – Mixed (in- person and virtual) (Baseline: NR, Follow-up: 1,084)<br>Arm 4 – Virtual care only (Baseline: NR, Follow-up: 1,952)	ED/urgent care visit	NR	Arm 1 – Continuous data Baseline: NR Follow-up: Mean: 0 (SE 0)<br>Arm 2 – Continuous data Baseline: NR Follow-up: Mean: 0.048 (SE 0.012)<br>Arm 3 – Continuous data Baseline: NR Follow-up: Mean: 0.137 (SE 0.033)<br>Arm 4 – Continuous data Baseline: NR Follow-up: Mean: 0 (SE 0)	Ref: Arm 1, p=<0.001 (for all comparisons)	Age, sex, race, baseline value of outcome, person-year, overdispersion
Gaetani, 2021(23)	2c	Arm 1 – Pre-COVID: Individuals with Hereditary Hemorrhagic Telangiectasia (45)<br>Arm 2 – Post-COVID: Individuals with Hereditary Hemorrhagic Telangiectasia (receiving telehealth) (45)	ED visit or Hospitalization	244 Days	Arm 1 – 11 (24.4)<br>Arm 2 – 9 (20)	Ref: Arm 1<br>% change from baseline: -0.044, p=not significant	No
Reddy, 2021(56)	2c	Arm 1 – Before virtual care (in-person) (763)<br>Arm 2 – Transition to virtual care (168)<br>Arm 3 – After transition to virtual care (813)	ED visit	4 weeks before transition	Arm 1 – 24 (3.1)<br>Arm 2 – 2 (1.2)<br>Arm 3 – 7 (0.9)	Ref: Arm 1, p=0.0031	No
Walker, 2023(71)	2c	Arm 1 – In-person (20,666)<br>Arm 2 – Telehealth (10,988)	ED visits	NR	Arm 1 – NR (0.42)<br>Arm 2 – NR (0.338)	Ref: Arm 1<br>Likelihood of ED visit: -0.003 (95% CI: -0.011 to 0.004), p=0.351	Baseline characteristic, facility fixed effect
Watson, 2021(72)	2c	Arm 1 – Pre- telephone clinic (face to face) (814)<br>Arm 2 – Post- introduction (telephone) (910)	Presentations (excluding hospitalization) within 24- hours	24 hours	Arm 1 – 3 (0.37)<br>Arm 2 – 4 (0.44)	Ref: Arm 1, p=1	No
		Arm 1 – Pre- telephone clinic (face to face) (814)<br>Arm 2 – Post- introduction (telephone) (910)	Presentations (excluding hospitalization) within 7 days	7 days	Arm 1 – 3 (0.37)<br>Arm 2 – 7 (0.77)	Ref: Arm 1, p=0.343	No

1a=general medical care, adults; 1b=general medical care, children; 1c=general medical care, all ages; 2s=specialized care, COVI-19; 2b=specialized care, pregnancy/prenatal/gynecological; 2c=specialized care, other; 3=general behavioral/mental health. CI=confidence internal; ED=emergency department; N=sample size; NR=not reported; OR=odds ratio; p=p-value; Ref=reference.

Supplementary Table 4-B. Study Results Investigating the Effectiveness of Telehealth vs. In-person Care by Outcome Category and Clinical Area – Healthcare Utilization, Hospitalization.

Author, Year	Clinical Area	Arm Definition (n)	Outcome	Time of Analysis	Participants With Outcomes, n (%)	Comparison	Adjusted
Ryskina, 2021(60)	1a	Arm 1 - Patients having in-person primary care appointment (6792)<br>Arm 2 - Patients having telehealth primary care appointment (10311)	Any Hospitalization	NR	NR	Ref: Arm 1, Odds ratio: 0.72 (95% CI: 0.57 to 0.9), p=0.004	Age, sex, race, ethnicity, and the weighted CCI

Author, Year	Clinical Area	Arm Definition (n)	Outcome	Time of Analysis	Participants With Outcomes, n (%)	Comparison	Adjusted
Zimmerman, 2021(77)	1a	Arm 1 - In-person (207)<br>Arm 2 – Telehealth (207)	Transfer to in-patient care	NR	Arm 1 - Events: 7.038 (3.4)<br>Arm 2 - Events: 2.898 (1.4)	p=NS	No
Shah, 2022(63)	1a	Arm 1 - In-person ED visits (11,818)<br>Arm 2 - Telehealth ED visits (5,169)	Hospitalization after ED visit	30 days	Arm 1 - Events: 438 (0.04)<br>Arm 2 - Events: 238 (0.05)	Ref: Arm 1<br>Odds ratio: 1.31 (95% CI: 1.09 to 1.58), p=0.007	Sociodemographic and clinical factors, time from ED discharge to follow-up
Hatef, 2022(28)	1c	Arm 1 - First encounter in- person (493,716)<br>Arm 2 - First encounter telemedicine (113,857)	Acute ambulatory care, Hospitalization on follow-up	14 days	NR (NR)	Ref: Arm 1<br>Odds ratio: 1.03 (95% CI: 0.98 to 1.08), p=NR	Type of acute and chronic ambulatory care sensitive conditions treated during the episode.
		Arm 1 - First encounter in- person (410,743)<br>Arm 2 - First encounter telemedicine (94,481)	Chronic ambulatory care, Hospitalization on follow-up	14 days	NR (NR)	Ref: Arm 1<br>Odds ratio: 0.94 (95% CI: 0.9 to 0.99), p=NR	Type of acute and chronic ambulatory care sensitive conditions treated during the episode.
Phillips, 2021(52)	1c	Arm 1 - Individuals presenting to initial in-person visit at Respiratory Assessment center (741)<br>Arm 2 - Individuals presenting to initial telehealth visit at Respiratory Assessment Center (564)	Hospital admission	14 Days	Arm 1 - 21 (2.8)<br>Arm 2 - 11 (2)	Ref: Arm 1<br>Chi-squared: NR, p=0.307	No
Casariego-Vales, 2021(12)	2a	Arm 1 - Primary Care Monitoring (3197)<br>Arm 2 - TELEA Telehealth Monitoring (1187)	Hospitalization	68 days	Arm 1 - 65 (2)<br>Arm 2 - 184 (NR)	Ref: Arm 1, Assumed t-test/man Whitney: NR, p≤.0001	No
			Hospitalization after ED visit	68 days	Arm 1 - 65 (28.6)<br>Arm 2 - 184 (NR)	Ref: Arm 1, Assumed t-test/man Whitney: NR, p≤.0001	No
Korycinski, 2022 (36)	2a	Arm 1 - Control (154)<br>Arm 2 - Telephone based intervention (139)	Hospital admission	30 days	Arm 1 - 10 (6.5)<br>Arm 2 - 6 (4.3)	Ref: Arm 1, Hazard ratio: 0.578 (95% CI: 0.29 to 1.13), p=0.452	No
Arias, 2022(5)	2b	Arm 1 - Pre-telehealth Implementation (780)<br>Arm 2 - Post-telehealth Implementation (799)	Intensive care nursery	NR	Arm 1 - 102 (13.1)<br>Arm 2 - 115 (14.4)	Ref: Arm 1<br>p=0.45	No
Duryea, 2021(19)	2b	Arm 1 - Pregnant individuals who received in-person pre-natal Care (6,559)<br>Arm 2 - Pregnant individuals who received audio telehealth and in- person prenatal care (6,048)	Full Term NICU Admission	183 Days	Arm 1 - 98 (1.5)<br>Arm 2 - 94 (1.6)	Ref: Arm 1<br>Relative risk: 1.03 (95% CI: 0.78 to 1.36), p=0.78	BMI at delivery, race

Author, Year	Clinical Area	Arm Definition (n)	Outcome	Time of Analysis	Participants With Outcomes, n (%)	Comparison	Adjusted
Afonso Nogueira, 2021(3)	2c	Arm 1 - Individuals with heart failure attending outpatient cardiology appointments (Pre-COVID) (160)<br>Arm 2 - Individuals with heart failure attending outpatient cardiology appointments (43)	Hospitalized for Heart Failure	497 days	Arm 1 - 71 (44.4)<br>Arm 2 - 11 (25.6)	Ref: Arm 1, Chi-squared: NR, p=0.83	No
Cvietusa, 2022(17)	2c	Arm 1 - No asthma care (Baseline: NR, Follow-up: 2,977)<br>Arm 2 - In-person only (Baseline: NR, Follow-up: 1,792)<br>Arm 3 - Mixed (in- person and virtual) (Baseline: NR, Follow-up: 1,084)<br>Arm 4 - Virtual care only (Baseline: NR, Follow-up: 1,952)	Hospitalization	NR	Arm 1 - Continuous data Baseline: NR Follow-up: Mean: 0 (SE 0)<br>Arm 2 - Continuous data Baseline: NR Follow-up: Mean: <0.001 (SE 0.062)<br>Arm 3 - Continuous data Baseline: NR Follow-up: Mean: <0.001 (SE 0.291)<br>Arm 4 - Continuous data Baseline: NR Follow-up: Mean: 0 (SE 0)	Ref: Arm 1, p=0.034 (for all comparisons)	Age, sex, race, baseline value of outcome, person-year, overdispersion
D'Anna, 2021(18)	2c	Arm 1 - In-person (2019) (180)<br>Arm 2 - Telephone (2020) (136)	Admission to hospital for recurrent transient ischemic attack/stroke	3 months	Arm 1 - 3 (1.67)<br>Arm 2 - 2 (1.47)	Ref: Arm1, p=0.445	No
Ostberg, 2022(50)	2c	Arm 1 - In-person (2019) (455)<br>Arm 2 – Telehealth/zoom (2020) (455)	Admit to in- patient	NR	Arm 1 - 27 (5.9)<br>Arm 2 - 29 (6.4)	Ref: Arm1, p=0.054	No
Severino, 2022(61)	2c	Arm 1 – Telehealth (92)<br>Arm 2 - In-person (92)	Cardiovascular hospitalization	12 months	Arm 1 - 14 (0.152)<br>Arm 2 - 19 (0.207)	Ref: Arm 1(telehealth) Hazard ratio: 1.34 (95% CI: 0.67 to 2.68), p=0.4	Age, gender, clinical characteristics, echocardiographic parameters
Sevilis, 2022(62)	2c	Arm 1: Pre- COVID (15,226)<br>Arm 2: COVID (11,105)	Inpatient thrombolytics	24 hours	Arm 1 - 66 (4)<br>Arm 2 - 70 (5.7)	Ref: Arm1, p=0.033	No
Sharma, 2020(64)	2c	Arm 1: IBD Services before COVID (2019) (1,036)<br>Arm 2: IBD Services after COVID (2020) (334)	Inpatient Contact	NR	Arm 1 - Events: 17 (1.6)<br>Arm 2 - Events: 3 (0.9)	Ref: Arm 1, % change from baseline: -0.82, p=NR	No
Walker, 2023(71)	2c	Arm 1 - In-person (20,666)<br>Arm 2 - Telehealth (10,988)	In-patient admission	NR	Arm 1 - NR (0.265)<br>Arm 2 - NR (0.228)	Ref: Arm 1 Likelihood of in-patient admission: 0.024 (95% CI: -0.018 to 0.031), p=<0.001	Baseline characteristic, facility fixed effect
Watson, 2021(72)	2c	Arm 1 - Pre- telephone clinic (face to face) (814)<br>Arm 2 - Post- introduction (telephone) (910)	Hospitalization within 24-hours	24 hours	Arm 1 - 18 (2.21)<br>Arm 2 - 22 (2.42)	Ref: Arm 1, p=0.531	No
Zhao, 2021(75)	2c	Arm 1 - Individuals with heart failure attending in-person outpatient appointment (39)<br>Arm 2 - Individuals with heart failure attending telehealth appointments (43)	Hospitalization for Heart Failure	66-82 days	Arm 1 - 0 (NR)<br>Arm 2 - 2 (NR)	NR	No

Author, Year	Clinical Area	Arm Definition (n)	Outcome	Time of Analysis	Participants With Outcomes, n (%)	Comparison	Adjusted
Zhu, 2021(76)	2c	Arm 1 - Arm 1 - In-person (2019) (1,286)<br>Arm 2 - Telephone (2020) (1,493)	Planned Admission or Procedure	NR	Arm 1 - 33 (2.6)<br>Arm 2 - 15 (1)	Ref: Arm 1<br>Odds ratio: 0.38 (95% CI: 0.208 to 0.71), p=0.002	No
			Unplanned admission	NR	Arm 1 - 53 (4.1)<br>Arm 2 - 39 (2.6)	Ref: Arm 1<br>Odds ratio: 0.62 (95% CI: 0.41 to 0.95), p=0.027	No

1a=general medical care, adults; 1b=general medical care, children; 1c=general medical care, all ages; 2s=specialized care, COVI-19; 2b=specialized care, pregnancy/prenatal/gynecological; 2c=specialized care, other; 3=general behavioral/mental health. ACSC= ambulatory care sensitive conditions; CCI= Charlson Comorbidity Index; CI=confidence interval; ED=emergency department; N=sample size; NICU=neonatal intensive care unit; NR=not reported; NS=non-significant; OR=odds ratio; p=p-value; Ref=reference.

Supplementary Table 4-C. Study Results Investigating the Effectiveness of Telehealth vs. In-person Care by Outcome Category and Clinical Area – Healthcare Utilization, Readmission.

Author, Year	Clinical Area	Arm Definition (n)	Outcome	Time of Analysis	Participants With Outcomes, n (%)	Comparison	Adjusted
Borgen, 2021(8)	2a	Arm 1 - Individuals with new COVID-19 not receiving telehealth care management discharged from the hospital (593)<br>Arm 2 - Individuals with new COVID-19 receiving telehealth care management (114)	Hospital Readmission	30 days	Arm 1 - 26 (4.4)<br>Arm 2 - 4 (3.5)	Ref: Arm 1, Chi-squared test of independence: 0.18, p=0.6	No
Carlberg, 2020(11)	2a	Arm 1 - Patients receiving telehealth and in- person evaluation in the ED (132)<br>Arm 2 - Patients receiving telehealth only evaluation in the ED (153)	Admitted upon return, within 72 hours, COVID-19 related	72 hours	Arm 1 - 1 (8)<br>Arm 2 - 0 (0)	NR	No
			Admitted upon return, Within 72 hours, Non-COVID-19 related	72 hours	Arm 1 - 0 (0)<br>Arm 2 - 0 (0)	NR	No
Khosla, 2022(33)	2b	Arm 1 - Pre- pandemic (215)<br>Arm 2 – Post-pandemic (258)	Readmission	6 weeks	Arm 1 - 38 (17.8)<br>Arm 2 - 45 (17.4)	Ref: Arm 1, p=0.91	No
Hughes, 2022(29)	2c	Arm 1 – In-person follow-up (NR)<br>Arm 2 – Telehealth follow-up (146)	Readmission	30 days	Arm 1 - 6 (0.171)<br>Arm 2 - 20 (0.137)	NR	No

1a=general medical care, adults; 1b=general medical care, children; 1c=general medical care, all ages; 2a=specialized care, COVID-19; 2b=specialized care, pregnancy/prenatal/gynecological; 2c=specialized care, other; 3=general behavioral/mental health. CI=confidence interval; N=sample size; NR=not reported; OR=odds ratio; p=p-value; Ref=reference.

Supplementary Table 5-A. Study Results Investigating the Effectiveness of Telehealth vs. In-person Care by Outcome Category and Clinical Area – Clinical Outcomes, Mortality.

Author, Year	Clinical Area	Arm Definition (n)	Outcome	Time of Analysis	Participants With Outcomes, n (%)	Comparison	Adjusted
Aiken, 2021(4)	2b	Arm 1 - Individuals receiving medical abortion following in-person visit (22,158)<br>Arm 2 - Individuals receiving medical abortion following telemedicine/hybrid visit (29,984)	Adverse Effect: Death	59 days (Arm 1)<br>85 days (Arm 2)	Arm 1 - 0 (0)<br>Arm 2 - 0 (0)	Ref: Arm 1<br>Covariate adjusted test of difference or proportions: NR, p=NR	Patient age, race/ethnicity, gestational age, parity and prior abortions using logistic regression and weighted risk differences.
Arias, 2022(5)	2b	Arm 1 - Pre-telehealth implementation (780)<br>Arm 2 - Post-telehealth implementation (799)	Fetal death	NR	Arm 1 - 11 (1.4)<br>Arm 2 - 13 (1.6)	Ref: Arm 1, p=0.72	No
Afonso Nogueira, 2021(3)	2c	Arm 1 - Individuals with heart failure attending outpatient cardiology appointments (Pre-COVID) (160)<br>Arm 2 - Individuals with heart failure attending outpatient cardiology appointments (43)	Mortality	497 days	Arm 1 - 20 (12.5)<br>Arm 2 - 1 (2.3)	Ref: Arm 1<br>Test of real vs estimated mortality: NR, p=NR	No
Severino, 2022(61)	2c	Arm 1 – Telehealth (92)<br>Arm 2 - In-person (92)	Mortality	12 months	Arm 1 - 10 (0.109)<br>Arm 2 - 11 (0.12)	Ref: Arm 1(telehealth)<br>Hazard ratio: 1.09 (95% CI: 0.46 to 2.56), p=0.4	Age, gender, clinical characteristics, echocardiographic parameters
Watson, 2021(72)	2c	Arm 1 - Pre- telephone clinic (face to face) (814)<br>Arm 2 - Post- introduction (telephone) (910)	30-day mortality post systemic therapy	30 days	Arm 1 - 7 (0.86)<br>Arm 2 - 0 (0)	Ref: Arm 1, p=0.008	No
Zhao, 2021(75)	2c	Arm 1 - Individuals with heart failure attending in-person outpatient appointment (39)<br>Arm 2 - Individuals with heart failure attending telehealth appointments (43)	Mortality and Major Cardiovascular Event	66-82 days	Arm 1 - NR (5.1)<br>Arm 2 - NR (2.33)	Ref: Arm 1<br>Assumed Chi-squared: NR, p=0.6	No

1a=general medical care, adults; 1b=general medical care, children; 1c=general medical care, all ages; 2a=specialized care, COVID-19; 2b=specialized care, pregnancy/prenatal/gynecological; 2c=specialized care, other; 3=general behavioral/mental health. CI=confidence interval; N=sample size; NR=not reported; OR=odds ratio; p=p-value; Ref=reference.

Supplementary Table 5-B. Study Results Investigating the Effectiveness of Telehealth vs. In-person Care by Outcome Category and Clinical Area – Clinical Outcomes, Patient-Reported Outcomes.

Author, Year	Clinical Area	Arm Definition (n)	Outcome	Time of Analysis	Participants With Outcomes, n (%)	Comparison	Adjusted
Arias, 2022(5)	2b	Arm 1 - Pre-telehealth Implementation (780)<br>Arm 2 - Post-telehealth Implementation (799)	Postpartum depression screening	NR	Arm 1 - 368 (65.1)<br>Arm 2 - 571 (86.3)	Ref: Arm 1<br>Odds ratio: 4.61 (95% CI: 3.38 to 6.28), p=<0.001	Prenatal care provider only
Bryson, 2023(10)	2b	Arm 1 - Telehealth (156)<br>Arm 2 - In-person (270)	Follow-up for Long acting reversible contraceptive; any symptom reported (pelvic pain, uterine bleeding,	NR	Arm 1 - 87 (55.8)<br>Arm 2 - 123 (45.6)	Ref: Arm 1 (telehealth)<br>Odds ratio: 1.4 (95% CI: 0.94 to 2.1), p=0.04	Patient age at the time of insertion, presence of menstrual or medical

Author, Year	Clinical Area	Arm Definition (n)	Outcome	Time of Analysis	Participants With Outcomes, n (%)	Comparison	Adjusted
			dysmenorrhea, acne, weight change, mental health change)				diagnoses at insertion, reason for long-acting reversible contraception (LARC), type of LARC device, and timing of follow-up visit.
Minsky, 2021(47)	2c	Arm 1 - Not using telemedicine (228)<br>Arm 2 - Using telemedicine (51)	Deterioration in dietary habit score	NR	Arm 1 - 97 (42.54)<br>Arm 2 - 17 (33.33)	NR	No
Kablinger<br>,<br>2022(31)	3	Arm 1 – Pre-pandemic (196)<br>Arm 2 – Post-pandemic (196)	BASE-6	NR	Arm 1 -Continuous data Baseline: NR<br>Follow-up: Mean: 23.91 (NR)<br>Arm 2 - Arm 1 - Continuous data Baseline: NR<br>Follow-up: Mean: 21.01 (NR)	Ref: Arm 1<br>Difference: p=<0.00	No
		Arm 1 – Pre-pandemic (199)<br>Arm 2 – Post-pandemic (199)	GAD-7	NR	Arm 1 - Continuous data Baseline: NR<br>Follow-up: Mean: 10.55 (NR)<br>Arm 2 - Continuous data Baseline: NR<br>Follow-up:<br>Mean: 8.39 (NR)	Ref: Arm 1<br>Difference: p=<0.00	No
		Arm 1 – Pre-pandemic (176)<br>Arm 2 – Post-pandemic (176)	PHQ-9	NR	Arm 1- Continuous data Baseline: NR<br>Follow-up: Mean: 11.88 (NR)<br>Arm 2 - Continuous data Baseline: NR<br>Follow-up: Mean: 9.7 (NR)	Ref: Arm 1<br>Difference: p=0.00	No
Levinson, 2021(37)	3	Arm 1 – In-person (60)<br>Arm 2 – Telehealth, zoom (33)	Eating Disorder Examination Questionnaire v4 (EDE-Q-IV)	Mean stay: 11.32 weeks Discharge	Arm 1 - Continuous data Baseline: Mean: 4.1 (SD 1.07)<br>Follow-up: Mean: 2.73 (SD 1.24)<br>Arm 2 - Continuous data Baseline: Mean: 3.56 (SD 1.42)<br>Follow-up: Mean: 2.56 (SD 1.14)	Ref: Arm 1<br>p=NS	No
			Beck Depression Inventory II (BDI-II)	Mean stay: 11.32 weeks Discharge	Arm 1 - Continuous data Baseline: Mean: 31.2 (SD 11.64)<br>Follow-up: Mean: 23.37 (SD 14.42)<br>Arm 2 - Continuous data	Ref: Arm 1<br>p=NS	No

Author, Year	Clinical Area	Arm Definition (n)	Outcome	Time of Analysis	Participants With Outcomes, n (%)	Comparison	Adjusted
					Baseline: Mean: 26.16 (SD 12.62)<br>Follow-up: Mean: 20.13 (SD 11.8)		
Liou, 2022(40)	3	Arm 1 – In-person (Baseline: 242, Follow-up: 217)<br>Arm 2 – Telehealth (Baseline: 25, Follow-up: 15)	Patient Health Questionnaire-9 (PHQ-9)	NR	NR	Ref: Arm 1<br>Mean change from baseline: 0.26 (95% CI: -2.66 to 3.18), p=NR	Total number of sessions, PTSD, grief, and pain
			General Anxiety Disorder-7 (GAD-7)	NR	NR	Ref: Arm 1<br>Mean change from baseline: 0.16 (95% CI: -2.34 to 2.67), p=NR	Total number of sessions, PTSD, grief, and pain
Rene, 2022(57)	3	Arm 1 – In-person (157)<br>Arm 2 – Telehealth (181)	Depression severity (PHQ-9)	NR	Arm 1 - Continuous data<br>Baseline: Mean: 11.5 (SD 6.4)<br>Follow-up: Mean: 8.7 (SD 6.3)<br>Arm 2 - Continuous data<br>Baseline: Mean: 11.8 (SD 5.7)<br>Follow-up: Mean: 8.9 (SD 6.2)	Ref: Arm 1<br>p=<0.001	Treatment cohort, age, sex, race, initial score
			Anxiety severity (GAD-7)	NR	Arm 1 - Continuous data<br>Baseline: Mean: 11 (SD 4.9)<br>Follow-up: Mean: 8.7 (SD 5.5)<br>Arm 2 - Continuous data<br>Baseline: Mean: 12.9 (SD 4.9)<br>Follow-up: Mean: 9.8 (SD 5.6)	Ref: Arm 1<br>p=<0.001	Treatment cohort, age, sex, race, initial score
Zayde, 2021(74)	3	Arm 1 – Pre-pandemic (12)<br>Arm 2 – Post-pandemic (12)	Patient Health Questionnaire–9 score	20 Weeks	Arm 1 - Continuous data<br>Baseline: Mean: 7.8 (SD 5.96)<br>Arm 2 - Follow-up: Mean: 3.1 (SD 2.02)	Cohen's d: -0.75 (95% CI: -0.62 to 8.78), p=NR	No
			Generalized Anxiety Disorder Scale–7 score (before vs after)	20 Weeks	Arm 1 - Continuous data<br>Baseline: Mean: 8.2 (SD 5.47)<br>Arm 2 - Follow-up: Mean: 2.8 (SD 1.75)	Cohen's d: -0.94 (95% CI: 2.23 to 8.57), p=NR	No

1a=general medical care, adults; 1b=general medical care, children; 1c=general medical care, all ages; 2a=specialized care, COVID-19; 2b=specialized care, pregnancy/prenatal/gynecological; 2c=specialized care, other; 3=general behavioral/mental health. BASE-6= Brief adjustment scale-6; BDI-II= Beck Depression Inventory II; CI=confidence interval; EDE-Q-IV= Eating Disorder Examination Questionnaire version 4; GAD-7= Generalized anxiety disorder-7; N=sample size; NR=not reported**;** NS=non-significant; p=p-value; PHQ-9= The patient health questionnaire -9; Ref=reference; SD=standard deviation

Supplementary Table 5-C. Study Results Investigating the Effectiveness of Telehealth vs. In-person Care by Outcome Category and Clinical Area – Clinical Outcomes, Condition Specific Outcomes.

Author, Year	Clinical Area	Arm Definition (n)	Outcome	Time of Analysis	Participants With Outcomes, n (%)	Comparison	Adjusted
Griebeler, 2022(27)	1a	Arm 1 - In-person (35)<br>Arm 2 - Telehealth (35)	5% weight change	12 weeks	Arm 1 - (70.5)<br>Arm 2 - (64.7)	Ref: Arm 1<br>Odds ratio: 0.9 (95% CI: 0.6 to 1.3), p=NR	No
Aiken, 2021(4)	2b	Arm 1 - Individuals receiving medical abortion following in-person visit (22,158)<br>Arm 2 - Individuals receiving medical abortion following telemedicine/hybrid visit (29,984)	Successful Medical Abortion	59 days (Arm 1)<br>85 days (Arm 2)	Arm 1 – 21,769 (0.982)<br>Arm 2 – 29,618 (0.988)	Ref: Arm 1<br>Covariate adjusted test of difference or proportions: NR, p=1	Patient age, race/ethnicity, gestational age, parity and prior abortions using logistic regression and weighted risk differences.
Arias, 2022(5)	2b	Arm 1 - Pre-telehealth implementation (780) Arm 2 - Post-telehealth implementation (799)	Any breastfeeding at postpartum visit	NR	Arm 1 - 420 (75.3)<br>Arm 2 - 473 (72.3)	Ref: Arm 1,<br>Odds ratio: 0.09 (95% CI: 0.68 to 1.18), p=0.25	No
Clark, 2022(14)	2b	Arm 1 - In-person (208)<br>Arm 2 - Telehealth (295)	C-section	NR	Arm 1 - 110 (52.8)<br>Arm 2 - 157 (53.2)	Ref: Arm 1,<br>p=0.941	No
			NICU admission		Arm 1 - 38 (18.3)<br>Arm 2 - 68 (23)	Ref: Arm 1<br>p=0.296	No
Gao, 2022(25)	2b	Arm 1 - In-person (938)<br>Arm 2 - Telehealth (938)	Pre-term birth <37 weeks	37 weeks	Arm 1 - 127 (13.5)<br>Arm 2 – 153 (16.3)	Ref: Arm 1,<br>p=0.76	Matched on age, race, ethnicity, and prenatal conditions, including hypertensive disorder, gestational diabetes, and infections of genitourinary tract in pregnancy
Barequet, 2021(6)	2c	Arm 1 - In-person/Office (NR)<br>Arm 2 - Telehealth (204)	Diagnostic accuracy and reliability for detecting keratoconus patients' progression - Specificity between office and remote	NR	Arm 1 - Continuous data Baseline: NR<br>Arm 2 - Follow-up: Specificity:<br>95.8 (95% CI NR)	NR	No
			Diagnostic accuracy and reliability -	NR	Arm 1 - Continuous data Baseline: NR<br>Arm 2 - Follow-up:	NR	No

Author, Year	Clinical Area	Arm Definition (n)	Outcome	Time of Analysis	Participants With Outcomes, n (%)	Comparison	Adjusted
			Sensitivity between office and remote		Sensitivity:<br>69.2 (95% CI NR)		
			Diagnostic accuracy and reliability - Positive predictive value between office and remote	NR	Arm 1 - Continuous data Baseline: NR<br>Arm 2 - Follow-up: 52.9 (95% CI NR)	NR	No
			Diagnostic accuracy and reliability - Negative predictive value between office and remote	NR	Arm 1 - Continuous data Baseline: NR<br>Arm 2 - Follow-up: 97.9 (95% CI NR)	NR	No
Cvietusa, 2022(17)	2c	Arm 1 - No asthma care (Baseline: NR, Follow-up: 2,977)<br>Arm 2 - In-person only (Baseline: NR, Follow-up: 1,792)<br>Arm 3 - Mixed (in- person and virtual) (Baseline: NR, Follow-up: 1,084)<br>Arm 4 - Virtual care only (Baseline: NR, Follow-up: 1,952)	Asthma exacerbations	NR	Arm 1 - Continuous data Baseline: NR<br>Follow-up: Mean: 0.013 (SE 0.003)<br>Arm 2 – Continuous data Baseline: NR<br>Follow-up: Mean: 0.127 (SE 0.015)<br>Arm 3 - Continuous data Baseline: NR<br>Follow-up: Mean: 0.537 (SE 0.055)<br>Arm 4 - Continuous data Baseline: NR<br>Follow-up: Mean: 0.161 (SE 0.018)	Ref: Arm 1, p=<0.001 (for all comparisons)	No
Fredwall, 2021(21)	2c	Arm 1 - Patients seen in- person at Epilepsy Clinic (101)<br>Arm 2 - Patients seen by telemedicine at Epilepsy Clinic (16)	In remission or had improvements, 1 month	1 month	Arm 1 - NR (0.7)<br>Arm 2 - 14 (0.88)	NR	No
			In remission or had improvements, 3 months	3 months	Arm 1 - NR (0.75)<br>Arm 2 - 14 (0.88)	NR	No
Mair, 2021(42)	2c	Arm 1 - Pre-COVID (2019) (210)<br>Arm 2 - Tele-rheumatology (340)	Disease in remission	NR	Arm 1 - 162 (77.1)<br>Arm 2 - 291 (85.6)	Ref: Arm1<br>Difference in proportion : 0.08 (95% CI: 0.02 to 0.15), p=Significant	NR
Minsky, 2021(47)	2c	Arm 1 - Not using telemedicine (228)<br>Arm 2 - Using telemedicine (51)	Likely to lose weight	NR	NR	Ref: Arm1<br>Odds ratio: 2.79 (95% CI: 1.04 to 7.48), p=0.042	Gender, age, baseline weight, treatment, exercise level, mood, dietary score

Author, Year	Clinical Area	Arm Definition (n)	Outcome	Time of Analysis	Participants With Outcomes, n (%)	Comparison	Adjusted
Tchang, 2022(69)	2c	Arm 1 - In-person (69)<br>Arm 2 - Hybrid (85)<br>Arm 3 - Video (91)	≥5% weight loss	6 months	Arm 1 - 32 (46.4)<br>Arm 2 - 47 (55.3)<br>Arm 3 - 54 (59.3)	Ref: Arm 1, p=0.26	No
Walker, 2023(71)	2c	Arm 1 - In-person (20,666)<br>Arm 2 - Telehealth (10,988)	Likelihood of HBA1C in control	NR	Arm 1 - NR (NR)<br>Arm 2 - NR (NR)	Ref: Arm 1 Likelihood estimate of HbA1C in control: 0.013 (: 0.002 to 0.024), p=<0.023	Baseline characteristic, facility fixed effect
Ye, 2022(73)	2c	Arm 1 - In-person (20,745)<br>Arm 2 -1 telemedicine visit (6,878)<br>Arm 3 - 2 or more telemedicine visits (5,104)	Not Meeting the Controlling High Blood Pressure quality measure (all hypertension patients)	NR	NR	Ref: Arm 1 Arm 2 - Odds ratio: 2.06 (95% CI: 1.94 to 2.18), p=<0.001<br>Ref: Arm 1 Arm 3 - Odds ratio: 2.49 (95% CI: 2.31 to 2.68), p=<0.001	No
Levinson, 2021(37)	3	Arm 1 - In-person (60)<br>Arm 2 -Telehealth, zoom (33)	Body Mass Index	Mean stay: 11.32 weeks Discharge	Arm 1 - Continuous data Baseline: NR Follow-up: Mean: 24.78 (SD 7.63)<br>Arm 2 - Continuous data Baseline: NR Follow-up: Mean: 26.26 (SD 10.39)	Ref: Arm 1 p=NS	No
Prato, 2022(55)	3	Arm 1 - In-person (20)<br>Arm 2 -Telehealth, zoom (20)	Yale Global Tic Severity Rating Scale (YGTSS)	2 months	Arm 1 - Continuous data Baseline: 25.8 (SD 7.3) Follow-up: Mean: 13.7 (SD 5.4), % change from baseline -7.7 (95% CI: -8.9 to -6.4)<br>Arm 2 - Continuous data Baseline: 25.5 (SD 10.5) Follow-up: Mean: 14.1 (SD 6.3), % change from baseline -6.6 (95% CI: -7.7 to -5.5)	Ref: Arm 1 , % change from baseline: 1.1 (95% CI: -0.6 to 2.7), p=NR	No
			Children's Yale-Brown Obsessive-Compulsive Scale for Children (CY-BOCS)	2 months	Arm 1 - Continuous data, Baseline: Mean: 22.7 (SD 12.7), Follow-up: Mean: 15.1 (SD 7.2), % change from baseline: -3.4 (95% CI: -4.6 to -2.2)<br>Arm 2 - Continuous data Baseline: Mean: 22.3 (SD 12), Follow-up: mean: 14.3 (SD 6.6), % change from baseline: -4.3 (95% CI: -5.4	Ref: Arm 1, % change from baseline: -0.9 (95% CI: -2.5 to 0.7), p=NR	No

Author, Year	Clinical Area	Arm Definition (n)	Outcome	Time of Analysis	Participants With Outcomes, n (%)	Comparison	Adjusted
					to -3.2)		
			Multidimensional Anxiety Scale for Children (MASC)	2 months	Arm 1 - Continuous data Baseline: Mean: 36.2 (SD 15.3), Follow-up: Mean: 22.6 (SD 5.4), % change from baseline: -5.1 (95% CI: -6.3 to -3.9)<br>Arm 2 - Continuous data Baseline: Mean: 35.1 (SD 16.8), Follow-up: mean: 21.6 (SD 10.1), % change from baseline: -6 (95% CI: -7.2 to -4.9)	Ref: Arm 1, % change from baseline: -1 (95% CI: -2.6 to 0.7), p=NR	No
1a=general medical care, adults; 1b=general medical care, children; 1c=general medical care, all ages; 2a=specialized care, COVID-19; 2b=specialized care, pregnancy/prenatal/gynecological; 2c=specialized care, other; 3=general behavioral/mental health. CI=confidence interval; N=sample size; NR=not reported; OR=odds ratio; p=p-value; Ref=reference. BP=blood pressure; CI=confidence interval; IQR=interquartile range; ml=milliliter; N=sample size; NR=not reported; OR=odds ratio; p=p-value; Ref=reference; SD=standard deviation; SE=standard error							

Supplementary Table 5-D. Study Results Investigating the Effectiveness of Telehealth vs. In-person Care by Outcome Category and Clinical Area – Clinical Outcomes, Adverse Events.

Author, Year	Clinical Area	Arm Definition (n)	Outcome	Time of Analysis	Participants With Outcomes, n (%)	Comparison	Adjusted
McNamara, 2021(45)	1a	Arm 1 - Face to face (341)<br>Arm 2 – Telehealth (151)	Average medication related problems per encounter	NR	Arm 1 - Continuous data Baseline: NR Follow-up: Mean: 1.65 (SD 1.56)<br>Arm 2 - Continuous data Baseline: NR Follow-up: Mean: 1.06 (SD 1.21)	Ref: Arm 1, p=<0.01	No
			Medication related problem: adverse drug event (per encounter)	NR	Arm 1 - Continuous data Baseline: NR Follow-up: Mean: 0.05 (SD 0.45)<br>Arm 2 - Continuous data Baseline: NR Follow-up: Mean: 0.12 (SD 0.34)	Ref: Arm 1, p=0.496	No
Aiken, 2021(4)	2b	Arm 1 - Individuals receiving medical abortion following in-person visit (22,158)<br>Arm 2 - Individuals receiving medical abortion following telemedicine/hybrid visit (29,984)	Adverse Effect: Major Surgery	59 days (Arm 1)<br>85 days (Arm 2)	Arm 1 - 0 (0)<br>Arm 2 - 0 (0)	Ref: Arm 1 Covariate adjusted test of difference or proportions: NR, p=NR	Patient age, race/ethnicity, gestational age, parity and prior abortions using logistic regression and weighted risk differences.

Author, Year	Clinical Area	Arm Definition (n)	Outcome	Time of Analysis	Participants With Outcomes, n (%)	Comparison	Adjusted
Duryea, 2021(15)	2b	Arm 1 - Pregnant individuals who received in-person pre-natal Care (6,559)<br>Arm 2 - Pregnant individuals who received audio telehealth and in- person prenatal care (6,048)	Composite Outcome (Still Birth, Full-term NICU Admission, Placental Abruption, Arterial Blood Gas pH <7.0	183 Days	Arm 1 - 195 (0.03)<br>Arm 2 - 173 (0.029)	Ref: Arm 1 Relative risk: 0.96 (95% CI: 0.78 to 1.17), p=0.71	BMI at delivery, race
Kerestes, 2021(32)	2b	Arm 1 - In person (94)<br>Arm 2 – Telemedicine + med pick up (124)<br>Arm 2 – Telemedicine + Mailed Medicine (69)	Complication: Blood Transfusion	252 days	Arm 1 - 0 (0)<br>Arm 2 - 2 (1.6)<br>Arm 3 - 0 (0)	NR	No
Pinsker,2021(54)	2c	Arm 1 - Individuals with diabetes receiving in-person insulin pump training (14,284)<br>Arm 2 - Individuals with diabetes receiving virtual insulin pump training (8,984)	Adverse Event	NR	Arm 1 - Continuous Baseline: NR Follow-up: Mean: 0.04 (SD 0.24)<br>Arm 2 - Continuous Baseline: NR Follow-up: Mean: 0.03 (SD 0.2)	Ref: Arm 1, p=0.003	Training method, age, previous therapy, trainer type, baseline A1c
Severino, 2022(61)	2c	Arm 1 - Telehealth (92)<br>Arm 2 - In-person (92)	Major adverse cardiovascular events	12 months	Arm 1 - 18 (0.197)<br>Arm 2 - 20 (0.217)	Ref: Arm 1(telehealth) Hazard ratio: 1.15 (95% CI: 0.61 to 2.19), p=0.65	Age, gender, clinical characteristic s, echocardiogr aphic parameters
Fortier, 2022(20)	3	Arm 1 - In-person (29)<br>Arm 2 - Telephone (45)	Adverse events	24 weeks	Arm 1 – 0 (0)<br>Arm 2 - 0 (0)	NR	No

1a=general medical care, adults; 1b=general medical care, children; 1c=general medical care, all ages; 2a=specialized care, COVID-19; 2b=specialized care, pregnancy/prenatal/gynecological; 2c=specialized care, other; 3=general behavioral/mental health. CI=confidence interval; N=sample size; NR=not reported; OR=odds ratio; p=p-value; Ref=reference. BMI=body mass index; CI=confidence interval; COPD=Chronic obstructive pulmonary disease; N=sample size; NA=not available; NR=not reported; OR=odds ratio; p=p-value; Ref=reference**;** SD=standard deviation

Supplementary Table 6-A. Study Results Investigating the Effectiveness of Telehealth vs. In-person Care by Outcome Category and Clinical Area – Process Outcomes, Missed Visits.

Author, Year	Clinical Area	Arm Definition (n)	Outcome	Time of Analysis	Participants With Outcomes, n (%)	Comparison	Adjusted
Mathews, 2022(43)	1a	Arm 1 - In-person (1,493)<br>Arm 2 - Telehealth (684)	Missed appointment	NR	Arm 1 - Events 143 (NR)<br>Arm 2 - Events 25 (NR)	Ref: Arm 1 percentage difference: -0.8252, p=<NR	No
Chen, 2023(13)	1a	Arm 1 - In-person, telehealth transition period (March 2020-June 2020) (52,697 visits)<br>Arm 2 - Telephone, telehealth transition period (March 2020-June 2020) (132,713 visits)<br>Arm 3 - Video, telehealth transition period (March 2020-June 2020) (98 visits)	Non-attendance	NR	Arm 1 - Events: NR (0.365)<br>Arm 2 - Events: NR (0.352)<br>Arm 3 - Events: NR (0.673)	Ref: Arm 1 Odds ratio (Arm 2 comparing to Arm 1): 1.04 (95% CI: 1.02 to 1.07), p=<0.001<br>Ref: Arm 1 Odds ratio (Arm 3 comparing to Arm 1): 4.37 (95% CI: 2.74 to 6.97), p=<0.001	Demographic factors and patient and clinic variation
		Arm 1 - In-person, elective telehealth period (July 2020-August 2021) (492,254 visits)<br>Arm 2 - Telephone, elective telehealth period (July 2020-August 2021) (305,197 visits)<br>Arm 3 - Video, elective telehealth period (July 2020-August 2021) (26,232 visits)	Non-attendance	NR	Arm 1 - Events: NR (0.252)<br>Arm 2 - Events: NR (0.212)<br>Arm 3 - Events: NR (0.355)	Ref: Arm 1 Odds ratio (Arm 2 comparing to Arm 1): 0.82 (95% CI: 0.81 to 0.83), p=<0.001<br>Ref: Arm 1 Odds ratio (Arm 3 comparing to Arm 1): 2.02 (95% CI: 1.95 to 2.08), p=<0.001	Demographic factors and patient and clinic variation
Adepoju, 2022(2)	1c	Arm 1 - In-person (207,621)<br>Arm 2 -Telemedicine (70,550)	Missed appointment	NR	Arm 1 - Events 38,750 (18.7)<br>Arm 2 – Events 11,113 (15.8)	Ref: Arm 1 Odds ratio: 0.87 (95% CI: 0.84 to 0.89), p=<0.001	Patient sociodemographic characteristics, geographic classification, medical appointment information, and clinic characteristics
Adams, 2023(1)	2b	Arm 1 - In-person (NR)<br>Arm 2 - Telehealth (NR)	Visit show rate	Peak Covid, 6-12 week Visit show rate	Arm 1 - NR (0.65)<br>Arm 2 - NR (0.76)	Ref: Arm 1, Relative risk: 1.17 (95% CI:	NR

Author, Year	Clinical Area	Arm Definition (n)	Outcome	Time of Analysis	Participants With Outcomes, n (%)	Comparison	Adjusted
						0.87 to 1.57), p=0.31	
				Peak Covid, <6 week Visit show rate	Arm 1 - NR (0.57)<br>Arm 2 - NR (0.96)	Ref: Arm 1, Relative risk: 1.7 (95% CI: 1.31 to 2.22), p=<0.001	
				Ongoing Covid, 6-12 week Visit show rate	Arm 1 - NR (0.74)<br>Arm 2 - NR (0.85)	Ref: Arm 1, Relative risk: 1.16 (95% CI: 0.9 to 1.5), p=0.26	
				Ongoing Covid, <6 week Visit show rate	Arm 1 - NR (0.88)<br>Arm 2 - NR (0.96)	Ref: Arm 1, Relative risk: 1.09 (95% CI: 0.93 to 1.29), p=0.29	
Arias, 2022(5)	2b	Arm 1 - Pre-telehealth Implementation (780)<br>Arm 2 - Post-telehealth Implementation (799)	Postpartum visit attendance rate	NR	Arm 1 - 565 (72.4)<br>Arm 2 - 662 (82.9)	Ref: Arm 1 Odds ratio: 1.9 (95% CI: 1.47 to 2.46), p=<0.001	Race, prenatal care provider, parity, gestation al age at delivery and insurance status
			Cardiology follow-up visit attendance rate	NR	Arm 1 - 29 (51.8)<br>Arm 2 - 36 (61)	Ref: Arm 1 Odds ratio: 1.8 (95% CI: 0.79 to 4.11), p=0.32	Prenatal care provider only
Clark, 2022(14)	2b	Arm 1 – In-person (1,253)<br>Arm 2 – Telehealth (1,411)	Missed appointment	NR	Arm 1 - Events 77 (0.0615)<br>Arm 2 - 17 (0.0121)	Ref: Arm 1, p=<0.001	No
Boshara, 2022(9)	2c	Arm 1 - In-person (332 visits)<br>Arm 2 - Telehealth (246 visits)	Appointment adherence	NR	Arm 1 - NR (70.8)<br>Arm 2 - NR (79.2)	Ref: Arm 1, p=<0.001	Paired data
Klain, 2021(34)	2c	Arm 1 - 2019 visit (corresponding period to follow-up visit during COVID-19) (525)<br>Arm 2 – 2020 telemedicine follow-up visit during COVID-19 (445)	Outpatient F/U evaluations	NR	NR	Ref: Evaluations in corresponding 2019 period Difference in number of evaluations: -0.15 (15% missed outpatient visit in 2020 comparing 2019), p=NR	No
Sohail, 2023(65)	2c	Arm 1 - In-person<br>Arm 2 - Video visit<br>Arm 3 - Telephone visit	Appointment no-show	NR	Arm 1 - Event NR (0.204)<br>Arm 2 - Event NR (0.122)<br>Arm 3 - Event NR (0.126)	Ref: Arm 1, Odds ratio (Arm 2 comparing to Arm 1): 0.61 (95% CI: 0.52 to 0.71),	Raw patient numbers not available, calculated based on percentages in text

Author, Year	Clinical Area	Arm Definition (n)	Outcome	Time of Analysis	Participants With Outcomes, n (%)	Comparison	Adjusted
						p=<0.05<br>Ref: Arm 1, Odds ratio (Arm 3 comparing to Arm 1): 0.57 (95% CI: 0.52 to 0.63), p=<0.05	
Sun, 2022(66)	2c	Arm 1 - In-person<br>Arm 2 - Telehealth	Appointment no-show	NR	Arm 1 - NR<br>Arm 2 - NR	Ref: Arm 1<br>Odds ratio: 0.43 (95% CI: 0.32 to 0.57), p=0	Multivariable random effects logistic regression
Watson, 2021(72)	2c	Arm 1 - Pre- telephone clinic (face to face) (814)<br>Arm 2 - Post- introduction (telephone) (910)	Proportion of patients who cancelled colonoscopies	NR	Arm 1 - 13 (1.5)<br>Arm 2 - 22 (2.5)	Ref: Arm 1<br>p-value only: p=0.14	No
Zhu, 2021(76)	2c	Arm 1 - Arm 1 - In-person (2019) (1,443)<br>Arm 2 - Telephone (2020) (1,597)	Appointment non-attendance	NR	Arm 1 - 157 (10.9)<br>Arm 2 - 104 (6.5)	Ref: Arm1<br>Odds ratio: 0.57 (95% CI: 0.44 to 0.739), p≤0.001	No
Rene, 2022(57)	3	Arm 1 – In-person (157)<br>Arm 2 – Telehealth (181)	Cancellation	NR	Arm 1 - Continuous data<br>Baseline: Mean: NR<br>Follow-up: Mean: 0.36 (SD 0.76)<br>Arm 2 - Continuous data<br>Baseline: NR<br>Follow-up: Mean: 0.45 (SD 0.81)	Ref: Arm 1<br>p=0.003	No
			No show	NR	Arm 1 - Continuous data<br>Baseline: Mean: NR<br>Follow-up: Mean: 0.25 (SD 0.54)<br>Arm 2 - Continuous data<br>Baseline: NR<br>Follow-up: Mean: 0.38 (SD 0.67)	Ref: Arm 1<br>p=0.26	No
Zayde, 2021(74)	3	Arm 1 – Pre-pandemic (12)<br>Arm 2 – Post-pandemic (12)	No show rates	20 Weeks	Arm 1 - Continuous data<br>Baseline: Mean: 0.23 (SD 0.23)<br>Arm 2 - Follow-up: Mean: 0.32 (SD 0.25)	NR	No

1a=general medical care, adults; 1b=general medical care, children; 1c=general medical care, all ages; 2a=specialized care, COVID-19; 2b=specialized care, pregnancy/prenatal/gynecological; 2c=specialized care, other; 3=general behavioral/mental health. CI=confidence interval; N=sample size; NR=not reported; OR=odds ratio; p=p-value; Ref=reference. BMI=body mass index; CI=confidence interval; COPD=Chronic obstructive pulmonary disease; N=sample size; NA=not available; NR=not reported; OR=odds ratio; p=p-value; Ref=reference**;** SD=standard deviation

Supplementary Table 6-B. Study Results Investigating the Effectiveness of Telehealth vs. In-person Care by Outcome Category and Clinical Area – Process Outcomes, Case Resolution/Duplication.

Author, Year	Clinical Area	Arm Definition (n)	Outcome	Time of Analysis	Participants With Outcomes, n (%)	Comparison	Adjusted
Jazayeri, 2022(30)	1b	Arm 1 - In-person (1,131)<br>Arm 2 - Telehealth (638)	Follow-up visits	NR	Arm 1 - Continuous Baseline: Mean: 1.208 (SD 0.443), Follow-up: NR<br>Arm 2 - Continuous Baseline: Mean: 1.21 (SD 0.495), Follow-up: NR	Ref: Arm 1<br>t-test between means: NR<br>p=0.922	No
Tarn, 2021(68)	1c	Arm 1 - Patients receiving in-person outpatient care at primary care clinic (52)<br>Arm 2 - Patients receiving telephone outpatient care at primary care clinic (55)<br>Arm 3 - Patients receiving telehealth(video) outpatient care at primary care clinic (89)	Telemedicine visit	NR	Arm 1 - Continuous Baseline: Mean: 1.1 (SD 0.3), Follow-up: NR<br>Arm 2 - Continuous Baseline: Mean: 1.16 (SD 0.46), Follow-up: NR<br>Arm 3 - Continuous Baseline: Mean: 2.3 (SD 0.7), Follow-up: NR	NR	No
Hatef, 2022(28)	1c	Arm 1 - First encounter in- person (493,716)<br>Arm 2 - First encounter telemedicine (113,857)	Acute ambulatory care, follow-up encounter	14 days	NR (NR)	Ref: Arm 1 Odds ratio: 1.44 (95% CI: 1.42 to 1.46), p=NR	Type of acute and chronic ambulatory care sensitive conditions treated during the episode.
		Arm 1 - First encounter in- person (410,743)<br>Arm 2 - First encounter telemedicine (94,481)	Chronic ambulatory care, follow-up encounter	14 days	NR (NR)	Ref: Arm 1 Odds ratio: 0.94 (95% CI: 0.92 to 0.95), p=NR	Type of acute and chronic ambulatory care sensitive conditions treated during the episode.
Carlberg, 2020(11)	2a	Arm 1 - Patients receiving telehealth and in- person evaluation in the ED (132)<br>Arm 2 - Patients receiving telehealth only evaluation in the ED (153)	Return to healthcare within 72 hours	72 hours	Arm 1 - 7 (5.3)<br>Arm 2 - 6 (3.9)	NR	No
			Admitted upon return, Within 72 hours, Non-COVID-19 related	72 hours	Arm 1 - 5 (3.8)<br>Arm 2 - 4 (2.6)	NR	No
Kerestes, 2021(32)	2b	Arm 1 - In person abortion service (94)<br>Arm 2 – Telemedicine + med pick up (124)<br>Arm 2 – Telemedicine + Mailed Medicine (69)	Abortion complication without surgery	243, 244, 245 days	Arm 1 - 88 (0.936)<br>Arm 2 - 120 (0.968)<br>Arm 3 - 67 (0.971)	NR	No
Fredwall, 2021(21)	2c	Arm 1 - Patients seen in- person at Epilepsy Clinic (101)<br>Arm 2 - Patients seen by telemedicine at Epilepsy Clinic (16)	Linked with counseling, 1 month	1 month	Arm 1 - NR (75)<br>Arm 2 - 8 (35)	NR	No
			Linked with counseling, 3 months	3 months	Arm 1 - NR (76)<br>Arm 2 - 10 (63)	NR	No

Author, Year	Clinical Area	Arm Definition (n)	Outcome	Time of Analysis	Participants With Outcomes, n (%)	Comparison	Adjusted
Kolb, 2021(35)	2c	Arm 1 - Patients receiving in-person outpatient care at ENT clinic (Recurrent Acute Otitis Media Cohort) (50)<br>Arm 2 - Patients receiving telehealth outpatient care at ENT clinic, (Recurrent Acute Otitis Media Cohort) (50)	Routine follow-up recommended	42 days	Arm 1 - 16 (32)<br>Arm 2 - 10 (20)	Ref: Arm 1 Chi- Squared Test: NR, p=0.254	No
Mossack, 2022(48)	2c	Arm 1 - In-person (25)<br>Arm 2 - Telephone (23)	>= 1 follow up	NR	Arm 1 - 16 (64)<br>Arm 2 - 18 (78)	Ref: Arm1, p=0.278	No
			30 day follow-up	30 days	Arm 1 - 7 (28)<br>Arm 2 - 9 (39)	Ref: Arm1, p=0.414	No
			60 day follow-up	60 days	Arm 1 - 14 (56)<br>Arm 2 - 15 (65)	Ref: Arm1, p=0.514	No
			90 day follow-up	90 days	Arm 1 - 15 (60)<br>Arm 2 - 18 (78)	Ref: Arm1, p=0.173	No
Zhu, 2021(76)	2c	Arm 1 - In-person (2019) (1,286)<br>Arm 2 - Telephone (2020) (1,493)	Follow-up phone call	NR	Arm 1 - 29 (2.3)<br>Arm 2 - 48 (3.2)	Ref: Arm 1 Odds ratio: 1.44 (95% CI: 0.901 to 2.293), p=0.127	No
Li, 2021(38)	2c	Arm 1 - Patients triaged in- person for ophthalmologic issue (51)<br>Arm 2 - Patients receiving virtual triage for ophthalmologic issue (183)	Need in- person ophthalmologic review	NR	Arm 1 - 400 (88.7)<br>Arm 2 - 220 (54.6)	Ref: Arm 1 Chi- Squared: 128.2, p≤0.001	No
			Return to the Emergency Room within 1 month	30 days	Arm 1 - 8 (15.7)<br>Arm 2 - 65 (35.5)	Ref: Arm 1 Chi- Squared: 7.31, p=0.007	No
Rowe, 2021(59)	2c	Arm 1 - Telephone Cardiology Outpatient Visits (1,118)<br>Arm 2 - Video Cardiology Outpatient Visits (327)	Follow-up appointment within study period	148 days	Arm 1 - 196 (16.5)<br>Arm 2 - 79 (24.2)	Ref: Arm 1, p=0.015	Age, Gender, English as First Language, Rural Status, initial appointment status, cardiologist seen
Offiah, 2022(49)	2c	Arm 1 - Traditional clinic (1,220)<br>Arm 2 - Virtual clinic (496)	Return clinic	NR	Arm 1 - 980 (80.3)<br>Arm 2 - 353 (71.2)	Ref: Arm1, p=0.0003	No
Lindhagen, 2022(39)	2c	Arm 1 - Pre-COVID-19 Pandemic (814)<br>Arm 2 - COVID-19 Pandemic (910)	Number of unplanned telephone contacts with doctor per patient	NR	Arm 1 - Continuous data Baseline: NR, Follow-up: Mean: 0.88 (SD 1.89)<br>Arm 2 - Continuous data Baseline: NR, Follow-up: Mean: 0.9 (SD 1.9)	Ref: Arm 1, p=0.379	No
Metcalfe, 2023(46)	2c	Arm 1 - In-person (75)<br>Arm 2 - Telemedicine (75)	Number of patients followed-up	NR	Arm 1 - 22 (29)<br>Arm 2 - 53 (71)	Ref: Arm 1 Odds ratio: 5.8 (95% CI: NR), p<0.001	No

1a=general medical care, adults; 1b=general medical care, children; 1c=general medical care, all ages; 2a=specialized care, COVID-19; 2b=specialized care, pregnancy/prenatal/gynecological; 2c=specialized care, other; 3=general behavioral/mental health. CI=confidence interval; N=sample size; NR=not reported; OR=odds ratio; p=p-value;

Author, Year	Clinical Area	Arm Definition (n)	Outcome	Time of Analysis	Participants With Outcomes, n (%)	Comparison	Adjusted
Ref=reference. BMI=body mass index; CI=confidence interval; COPD=Chronic obstructive pulmonary disease; N=sample size; NA=not available; NR=not reported; OR=odds ratio; p=p-value; Ref=reference; SD=standard deviation							

Supplementary Table 6-C. Study Results Investigating the Effectiveness of Telehealth vs. In-person Care by Outcome Category and Clinical Area – Process Outcomes, Change in Therapy/Medication.

Author, Year	Clinical Area	Arm Definition (n)	Outcome	Time of Analysis	Participants With Outcomes, n (%)	Comparison	Adjusted
McNamara, 2021(45)	1a	Arm 1 - Face to face (341)<br>Arm 2 - Telehealth (151)	Medication related problems: needed additional drug therapy (per encounter)	NR	Arm 1 - Continuous data<br>Baseline: NR<br>Follow-up: Mean: 0.37 (SD 0.7)<br>Arm 2 - Continuous data<br>Baseline: NR<br>Follow-up: Mean: 0.12 (SD 0.4)	Ref: Arm 1, p=0.527	No
			Medication related problems: different drug needed (per encounter)	NR	Arm 1 - Continuous data<br>Baseline: NR<br>Follow-up: Mean: 0.09 (SD 0.31)<br>Arm 2 - Continuous data<br>Baseline: NR<br>Follow-up: Mean: 0.06 (SD 0.23)	Ref: Arm 1, p=0.423	No
Wabe, 2022(70)	1a	Arm 1 - Face to face (8,303,233)<br>Arm 2 - Telehealth (5,304,983)	Consultations with at least one medication prescribed	NR	Arm 1 - 3,264,748 (39.3)<br>Arm 2 - 1,751,878 (33)	Ref: Arm 1 Odds ratio: 1.38 (95% CI: 1.379 to 1.381), p=NR	Age, sex, socioeconomic status, patient status, remoteness, primary health network, and the state of the practice
			Consultations with first-time medication prescribed	NR	Arm 1 - 1,520,401 (18.3)<br>Arm 2 – 537,144 (10.1	Ref: Arm 1<br>Odds ratio: 2.03 (95% CI: 2.02 to 2.031), p=NR	Age, sex, socioeconomic status, patient status, remoteness, primary health network, and the state of the practice

Author, Year	Clinical Area	Arm Definition (n)	Outcome	Time of Analysis	Participants With Outcomes, n (%)	Comparison	Adjusted
Cobo-Calvo, 2022(15)	2c	Arm 1 - Face to face 2018 (276)<br>Arm 2 - Face to face 2019 (360)<br>Arm 3 - Telehealth 2020 (289)<br>Arm 4 - Telehealth extension period (Jan-May 2021) (NR)	Mean monthly treatment prescriptions	NR	Arm 1 - Continuous data Baseline: NR, Follow-up: Mean: 23 (SD 8)<br>Arm 2 - Continuous data Baseline: NR, Follow-up: Mean: 30 (SD 7)<br>Arm 3 - Continuous data Baseline: NR, Follow-up: Mean: 24.1 (SD 7)<br>Arm 4 - Continuous data Baseline: NR, Follow-up: Mean: 23.2 (SD 5.5)	Ref: Arm 1 and Arm 2, Arm 3 compared to Arm1:p=0.727, compared to Arm2: p=0.049<br><br>Ref: Arm 2 and Arm 3, Arm 4 compared to Arm1: p=0.072, compared to Arm2: p=0.805	No
Lindhagen, 2022(39)	2c	Arm 1 - Pre-COVID-19 Pandemic (868)<br>Arm 2 - COVID-19 Pandemic (891)	Increased medication	NR	Arm 1 - NR (21.3)<br>Arm 2 - NR (22.2)	Ref: Arm 1, p=0.641	No
			Decreased medication	NR	Arm 1 - NR (6.1)<br>Arm 2 - NR (5.9)	Ref: Arm 1,p=0.914	No
Mair, 2021(42)	2c	Arm 1 - Pre-COVID (2019) (210)<br>Arm 2 – Tele-rheumatology (340)	Any medication change	NR	Arm 1 - 79 (37.6)<br>Arm 2 – 84 (24.7)	Ref: Arm1 Difference in proportion : 0.11 (95% CI: 0.04 to 0.19), p=Significant	No
			No change in disease-modifying antirheumatic drugs	NR	Arm 1 - 152 (72.4)<br>Arm 2 – 285 (73.9)	Ref: Arm1 Difference in proportion : -0.13 (95% CI: -0.21 to -0.05), p=Significant	No
Mossack, 2022(48)	2c	Arm 1 - In-person (25)<br>Arm 2 - Telephone (23)	Treatment change	NR	Arm 1 - 9 (NR)<br>Arm 2 - 8 (NR)	Ref: Arm1, p=0.9	No
Offiah, 2022(49)	2c	Arm 1 - Traditional clinic (1,220)<br>Arm 2 - Virtual clinic (496)	Patients ≥ 1 management change	NR	Arm 1 - 470 (38.5)<br>Arm 2 - 99 (19.9)	Ref: Arm1, p=<0.0000	No
Piga, 2022(53)	2c	Arm 1 - In-person (25)<br>Arm 2 - Telephone (23)	Treatment adjusted	NR	Arm 1 - 17 (0.16)<br>Arm 2 - 19 (0.179)	NR	No
			Treatment tapering/cessation	NR	Arm 1 - 9 (0.085)<br>Arm 2 - 11 (0.104)	NR	No
Severino, 2022(61)	2c	Arm 1 - In-person (92)<br>Arm 2 - Telehealth (92)	Change in therapy	12 months	Arm 1 - 15 (NR)<br>Arm 2 - 19 (NR)	Ref: Arm 1, p=0.9	Age, gender, clinical characteristics, echocardiographic parameters
Sharma, 2020(64)	2c	Arm 1 - IBD Services before COVID (2019) (50)<br>Arm 2 - IBD Services after COVID (2020) (45)	Medication dose escalation	32 days	Arm 1 - 8 (16)<br>Arm 2 - 8 (18)	Ref: Arm 1, Fisher's exact test: NR, p=>0.99	No

Author, Year	Clinical Area	Arm Definition (n)	Outcome	Time of Analysis	Participants With Outcomes, n (%)	Comparison	Adjusted
Zhu, 2021(76)	2c	Arm 1 - Arm 1 - In-person (2019) (1,286)<br>Arm 2 - Telephone (2020) (1,493)	Changes in analgesia	NR	Arm 1 - 96 (7.5)<br>Arm 2 - 79 (5.3)	Ref: Arm 1<br>Odds ratio:<br>0.69 (95% CI:<br>0.509 to 0.942),<br>p=0.019	No
			Clinicians change immunosuppressive therapy	NR	Arm 1 - 352 (27.4)<br>Arm 2 - 338 (22.6)	Ref: Arm 1<br>Odds ratio:<br>0.78 (95% CI:<br>0.654 to 0.923),<br>p=0.004	No

1a=general medical care, adults; 1b=general medical care, children; 1c=general medical care, all ages; 2a=specialized care, COVID-19; 2b=specialized care, pregnancy/prenatal/gynecological; 2c=specialized care, other; 3=general behavioral/mental health. CI=confidence interval; N=sample size; NR=not reported; OR=odds ratio; p=p-value; Ref=reference. BMI=body mass index; CI=confidence interval; COPD=Chronic obstructive pulmonary disease; N=sample size; NA=not available; NR=not reported; OR=odds ratio; p=p-value; Ref=reference**;** SD=standard deviation

Supplementary Table 6-D. Study Results Investigating the Effectiveness of Telehealth vs. In-person Care by Outcome Category and Clinical Area – Process Outcomes, Treatment/ Medication Adherence.

Author, Year	Clinical Area	Arm Definition (n)	Outcome	Time of Analysis	Participants With Outcomes, n (%)	Comparison	Adjusted
Griebeler, 2022(27)	1a	Arm 1 – In-person (24)<br>Arm 2 – Telehealth (30)	Full medication compliance	NR	Arm 1 – 18 (0.75)<br>Arm 2 – 24 (0.8)	Ref: Arm 1,<br>p=0.58	No
McNamara, 2021(45)	1a	Arm 1 – Face to face (341)<br>Arm 2 – Telehealth (151)	Medication related problem: non-adherence<br>(per encounter)	NR	Arm 1 – Continuous data<br>Baseline: NR<br>Follow-up: Mean: 0.01<br>(SD 0.14)<br>Arm 2 – Continuous data<br>Baseline: NR<br>Follow-up: Mean: 0.01 (SD 0.08)	Ref: Arm 1,<br>p=1	No
Zimmerman, 2021(77)	1a	Arm 1 – In-person (207)<br>Arm 2 – Telehealth (207)	Completed treatment	NR	Arm 1 – NR (62.3)<br>Arm 2 – NR (72.9)	Ref: Arm 1,<br>p=<0.05	No
Baughman, 2021(7)	2c	Arm 1 – Office (54,872)<br>Arm 2 – Telemedicine (8,850)<br>Arm 3 – Telemedicine+Office (63,722)	Adherent to treatment	6 months	Arm 1 – 33,053 (60.2)<br>Arm 2 – 4,960 (56)<br>Arm 3 – 38,013 (59.7)	Ref: Arm 1<br>Difference %: 4.2,<br>p=<0.001	No
Cvietusa, 2022(17)	2c	Arm 1 – No asthma care (Baseline: NR, Follow-up: 2,977)<br>Arm 2 – In-person only (Baseline: NR, Follow-up: 1,792)<br>Arm 3 – Mixed (in- person and virtual) (Baseline: NR, Follow-up: 1,084)<br>Arm 4 – Virtual care only (Baseline: NR, Follow-up: 1,952)	Proportion of days covered	NR	Arm 1 – Continuous data<br>Baseline: NR Follow-up:<br>Mean: 0.489 (SE 0.007)<br>Arm 2 – Continuous data<br>Baseline: NR Follow-up:<br>Mean: 0.446 (SE 0.008)<br>Arm 3 – Continuous data<br>Baseline: NR Follow-up:<br>Mean: 0.497 (SE 0.01)	Ref: Arm 1,<br>p=<0.001 (for all comparisons)	Age, sex, race, baseline value of outcome,

Author, Year	Clinical Area	Arm Definition (n)	Outcome	Time of Analysis	Participants With Outcomes, n (%)	Comparison	Adjusted
					Arm 4 – Continuous data Baseline: NR Follow-up: Mean: 0.476 (SE 0.008)		
Garmendia, 2021(26)	2c	Arm 1 – Individuals with Sleep Apnea attending outpatient clinic, pre-covid/in-person (193)<br>Arm 2 – Individuals with Sleep Apnea attending outpatient clinic, post-covid, telehealth (77)	CPAP Compliance	NR	NR	Ref: Arm 1 Chi-squared: NR, p=0.099	No
McCoy, 2022(44)	2c	Arm 1 – In-person before telemedicine (113)<br>Arm 2 – Telemedicine (59)<br>Arm 3 – In-person during telemedicine (4)	Surgery performed	NR	Arm 1 – 41 (36.3)<br>Arm 2 – 24 (40.7)<br>Arm 3 – 2 (50)	NR	No
Mossack, 2022(48)	2c	Arm 1 – In-person (25)<br>Arm 2 – Telephone (23)	Medication adherence	NR	Arm 1 – 12 (48)<br>Arm 2 – 12 (52)	Ref: Arm1, p=1	No
Cunningham, 2022(16)	3	Arm 1 – Pre-pandemic (in-person) (72)<br>Arm 2 – Pandemic (telemedicine) (35)	90-days treatment retention	90 days	Arm 1 – 24 (33.3)<br>Arm 2 – 17 (48.6)	NR	No
Fortier, 2022(20)	3	Arm 1 – In-person (29)<br>Arm 2 – Telehealth-virtual care (45)	Dropout	24 weeks	Arm 1 – 7 (24)<br>Arm 2 – 8 (18)	Ref: Arm 1 Chi-squared: NR, p=0.506	No
			Attendance	24 weeks	Arm 1 – NR (75)<br>Arm 2 – NR (88)	Ref: Arm 1 Z-score NR, p=0.007	No
Frost, 2022(22)	3	Arm 1 – In-person (1,158)<br>Arm 2 – Telehealth (3,180)	Treatment retention	90 days	Arm 1 – 294 (0.254)<br>Arm 2 – 1,024 (0.322)	Ref: Arm 1 Odds ratio: 1.31 (95% CI: 1.12 to 1.53), p=NR	Sociodemographic and clinical characteristics
Gainer, 2023(24)	3	Arm 1 – In-person (235)<br>Arm 2 – Telephone (222)<br>Arm 3 – Telehealth with video (87)	Treatment engagement	NR	Arm 1 – NR (NR)<br>Arm 2 – NR (NR)<br>Arm 3 – NR (NR)	Ref: Arm 1 Odds ratio (Arm 2 comparing to Arm 1): 2.12 (95% CI: 1.05 to 4.28), p=0.036<br>Ref: Arm 1 Odds ratio (Arm 3 comparing to Arm 1): 5.4 (95% CI: 1.92 to 15.2), p=0.001	Demographics, socioeconomic status, substance use history and other clinical characteristics
Ripp, 2022(58)	3	Arm 1 – In-person (2019) (1,077)<br>Arm 2 – Telehealth (2020) (354)	Completed follow-up visits	NR	NR	Ref: Arm 1 Odds ratio: 1.57 (95% CI: 1.23 to 2), p=<0.001	Age, sex, race, ethnicity, insurance type, and week of visit

1a=general medical care, adults; 1b=general medical care, children; 1c=general medical care, all ages; 2a=specialized care, COVID-19; 2b=specialized care, pregnancy/prenatal/gynecological; 2c=specialized care, other; 3=general behavioral/mental health. CI=confidence interval; N=sample size; NR=not reported; OR=odds ratio; p=p-value; Ref=reference. BMI=body mass index; CI=confidence interval; COPD=Chronic obstructive pulmonary disease; N=sample size; NA=not available; NR=not reported; OR=odds ratio; p=p-value; Ref=reference**;** SD=standard deviation

Supplementary Table 6-E. Study Results Investigating the Effectiveness of Telehealth vs. In-person Care by Outcome Category and Clinical Area – Process Outcomes, Up to Date Labs and Paraclinical Assessment.

Author, Year	Clinical Area	Arm Definition (n)	Outcome	Time of Analysis	Participants With Outcomes, n (%)	Comparison	Adjusted
Jazayeri, 2022(30)	1b	Arm 1 - In-person (1,131)<br>Arm 2 - Telehealth (638)	Imaging orders	3 months	Arm 1 - Continuous data Baseline: NR, Follow-up: Mean: 0.521 per patient (SD 0.852)<br>Arm 2 - Continuous data Baseline: NR, Follow-up: Mean: 0.364 (SD 0.646)	Ref: Arm 1<br>t-test between means, p=<0.001	No
			Lab orders	3 months	Arm 1 - Continuous data Baseline: NR, Follow-up: Mean: 4.867 per patient (SD 5.317)<br>Arm 2 - Continuous data Baseline: NR, Follow-up: Mean: 4.049 (SD 4.901)	Ref: Arm 1<br>t-test between means, p=0.001	No
			Procedures	3 months	Arm 1 - Continuous data Baseline: NR, Follow-up: Mean: 0.128 per patient (SD 0.334)<br>Arm 2 - Continuous data Baseline: NR, Follow-up: Mean: 0.122 (SD 0.328)	Ref: Arm 1<br>t-test between means, p=0.718	No
Arias, 2022(5)	2b	Arm 1 - Pre-telehealth Implementation – Diabetic Patients (45)<br>Arm 2 - Post-telehealth Implementation – Diabetic Patients (59)	Completion of postpartum glucose tolerance test	NR	Arm 1 - 12 (26.7)<br>Arm 2 - 15 (25.4)	Ref: Arm 1<br>Odds ratio: 0.99 (95% CI: 0.37 to 2.68), p=0.89	Race, insurance status, and length of hospital stay
Cobo-Calvo, 2022(15)	2c	Arm 1 - Face to face 2018 (2,207)<br>Arm 2 - Face to face 2019 (2,356)<br>Arm 3 - Telehealth 2020 (2,202)	Mean monthly visits	NR	Arm 1 - Continuous data Baseline: NR, Follow-up: Mean: 183.9 (SD 29.1)<br>Arm 2 - Continuous data Baseline: NR, Follow-up: Mean: 196 (SD 17.5)<br>Arm 3 - Continuous data Baseline: NR, Follow-up: Mean: 183.5 (SD 68.9)	Ref: Arm 1 and Arm 2, Arm 3 compared to Arm1:p=0.984, compared to Arm2: p=0.538	No
Lindhagen, 2022(39)	2c	Arm 1 - Pre-telephone clinic, face to face (814)<br>Arm 2 – Post introduction, telephone (910)	Ulcerative colitis, Proportion of patients with surveillance colonoscopy	NR	Arm 1 - 76 (15)<br>Arm 2 - 49 (9.4)	Ref: Arm 1, p=0.007	No
			Crohn's disease, Proportion of patients with surveillance colonoscopy	NR	Arm 1 - 17 (5.1)<br>Arm 2 - 11 (3.3)	Ref: Arm 1, p=0.224	No
Liu, 2021(41)	2c	Arm 1 - Pre-COVID-19 clinic patients at various outpatient facilities (492)<br>Arm 2 - Post-COVID-19 clinic patients at various outpatient facilities (582)	Pathology test/consult completed	5 days	Arm 1 - 426 (86.6)<br>Arm 2 - 443 (76.1)	Ref: Arm 1<br>Assumed t-test: NR, p≤0.001	No

Author, Year	Clinical Area	Arm Definition (n)	Outcome	Time of Analysis	Participants With Outcomes, n (%)	Comparison	Adjusted
		Arm 1 - Pre-COVID-19 clinic patients at various outpatient facilities (295)<br>Arm 2 - Post-COVID-19 clinic patients at various outpatient facilities (345)	Radiology test/consult completed	5 days	Arm 1 - 247 (83.7)<br>Arm 2 – 229 (66.4)	Ref: Arm 1<br>Assumed t-test: NR, p≤0.001	No
Ostberg, 2022(50)	2c	Arm 1 - In-person (2019) (455)<br>Arm 2 – Telehealth/zoom (2020) (455)	All order class (imaging, labs, medication, nursing)	NR	Arm 1 - Continuous data Baseline: NR, Follow-up: Median: 9 (IQR 6, 12)<br>Arm 2 - Continuous data Baseline: NR, Follow-up: Median: 10 (IQR 7, 14)	Ref: Arm 1<br>Rate Ratio: 1.19 (95% CI: 1.11 to 1.28, p=<0.00 1	Age, gender, billing level, insurance status, and length of stay
Parise, 2021(51)	2c	Arm 1 - Not included in telemedicine study (43)<br>Arm 2 - Included in telemedicine study (166)	Continuous glucose monitoring	NR	Arm 1 - 7 (16)<br>Arm 2 - 155 (93.4)	Ref: Arm 1, p=<0.001	No
Reddy, 2021(56)	2c	Arm 1 - Before virtual care (in-person) (763)<br>Arm 2 - Transition to virtual care (168)<br>Arm 3 - After transition to virtual care (813)	Laboratory testing	4 weeks before Transition, 1 week during transition, 4 weeks after transition	Arm 1 - 265 (34.7)<br>Arm 2 - 58 (34.5)<br>Arm 3 - 105 (12.9)	Ref: Arm 1, p=<0.0001	No
			Diagnostic imaging	4 weeks before Transition, 1 week during transition, 4 weeks after transition	Arm 1 - 112 (14.7)<br>Arm 2 - 17 (10.1)<br>Arm 3 - 40 (4.9)	Ref: Arm 1, p=<0.0001	No
			Procedures (biopsy, paracentesis, acupuncture, endoscopy, catheter exchanges, etc.)	4 weeks before Transition, 1 week during transition, 4 weeks after transition	Arm 1 - 16 (2.1)<br>Arm 2 - 1 (0.6)<br>Arm 3 - 5 (0.6)	Ref: Arm 1, p=0.0223	No
Szigety, 2022(67)	2c	Arm 1 - In-person (2,642)<br>Arm 2 - Transition to virtual care (1,685)	Genetic testing was recommended	NR	Arm 1 - NR (70.9)<br>Arm 2 - NR (79.5)	Ref: Arm1, p=<0.001	No
			Test completion rates	NR	Arm 1 - NR (55.1)<br>Arm 2 - NR (51.2)	Ref: Arm1, p=0.09	No

1a=general medical care, adults; 1b=general medical care, children; 1c=general medical care, all ages; 2a=specialized care, COVID-19; 2b=specialized care, pregnancy/prenatal/gynecological; 2c=specialized care, other; 3=general behavioral/mental health. CI=confidence interval; N=sample size; NR=not reported; OR=odds ratio; p=p-value; Ref=reference. BMI=body mass index; CI=confidence interval; COPD=Chronic obstructive pulmonary disease; N=sample size; NA=not available; NR=not reported; OR=odds ratio; p=p-value; Ref=reference; SD=standard deviation

Supplementary Table 7-A. Risk of Bias Assessment for Non-Randomized Studies.

Author, year	Domain 1: Confounding	Domain 2: Patient Selection	Domain 3: Classifying Interventions	Domain 4: Deviations from intended interventions	Domain 5: Missing data	Domain 6: Measurement of outcomes	Domain 7: Selection of reported results	Overall Assessment
Adams, 2023(1)	Serious	Moderate	Low	No information	Low	Low	Moderate	Serious
Adepoju, 2022(2)	Moderate	Low	Low	No information	Moderate	Low	Low	Moderate
Afonso Nogueira, 2021(3)	Critical	Low	Low	Low	Low	Low	Low	Serious
Aiken, 2021(4)	Moderate	Moderate	Low	Moderate	Low	Low	Low	Moderate
Arias, 2022(5)	Low	Low	Low	Low	Low	Low	Low	Low
Barequet, 2021(6)	Serious	No information	Low	Low	Low	Low	Low	Serious
Baughman, 2021(7)	Low	Low	Low	Low	Moderate	Low	Low	Moderate
Borgen, 2021(8)	Moderate	Low	Low	Low	Low	Low	Low	Moderate
Boshara, 2022(9)	Low	No information	Low	Low	Low	No information	Low	Moderate
Bryson, 2023(10)	Moderate	Low	Moderate	No information	Moderate	Low	Moderate	Moderate
Carlberg, 2020(11)	Serious	Moderate	Low	Low	Low	Low	Low	Serious
Casariego-Vales, 2021(12)	Serious	Serious	Low	Serious	Moderate	Moderate	Low	Serious
Chen, 2023(13)	Moderate	Low	Low	No information	Low	Low	Low	Moderate
Clark, 2022(14)	Serious	Moderate	Low	No information	No information	Low	Low	Serious
Cobo-Calvo, 2022(15)	Critical	Moderate	Low	Low	Low	Low	Low	Serious
Cunningham, 2022(16)	Critical	Low	Low	Moderate	No information	Low	Low	Serious
Cvietusa, 2022(17)	Low	Low	Low	Low	Low	Low	Low	Low
D'Anna, 2021(18)	Critical	Moderate	Low	Low	Low	Low	Low	Serious
Duryea, 2021(19)	Moderate	Low	Low	Low	Low	Low	Low	Serious
Fortier, 2022(20)	Serious	Low	Low	Low	Low	Serious	Low	Serious
Fredwall, 2021(21)	Serious	Low	Low	Low	Moderate	Low	Low	Serious
Frost, 2022(22)	Moderate	Low	Moderate	No information	Low	Low	Low	Moderate
Gaetani, 2021(23)	Moderate	Moderate	Low	Moderate	Low	Moderate	Low	Moderate
Gainer, 2023(24)	Moderate	Low	Moderate	Low	Moderate	Low	Moderate	Moderate
Gao, 2022(25)	Serious	Low	Low	No information	Low	Low	Low	Serious
Garmendia, 2021(26)	Low	Serious	Low	No information	Low	Low	Low	Moderate

Author, year	Domain 1: Confounding	Domain 2: Patient Selection	Domain 3: Classifying Interventions	Domain 4: Deviations from intended interventions	Domain 5: Missing data	Domain 6: Measurement of outcomes	Domain 7: Selection of reported results	Overall Assessment
Hatef, 2022(28)	Low	Low	Low	Low	Low	Low	Low	Low
Hughes, 2022(29)	Serious	Low	Low	No information	No information	Serious	Low	Serious
Jazayeri, 2022(30)	Serious	Low	Moderate	No information	Serious	Serious	Moderate	Serious
Kablinger, 2022(31)	Low	No information	Low	Low	Low	Low	Low	Moderate
Kerestes, 2021(32)	Moderate	Low	Low	Low	Moderate	Moderate	Moderate	Moderate
Khosla, 2022(33)	Low	Low	Low	Low	Low	Low	Low	Low
Klain, 2021(34)	Serious	Low	Low	Low	Low	Low	Low	Serious
Kolb, 2021(35)	Serious	Low	Serious	Low	Low	Low	Low	Serious
Korycinski, 2022(36)	Moderate	Low	Low	Low	No information	Low	Low	Moderate
Levinson, 2021(37)	Serious	Serious	Low	Serious	Moderate	Low	Low	Serious
Li, 2021(38)	Serious	Serious	Moderate	Low	Low	Moderate	Low	Serious
Lindhagen, 2022(39)	Critical	Low	Low	Low	Low	Low	Low	Serious
Liou, 2022(40)	Low	Moderate	Low	No information	Moderate	Low	Low	Moderate
Liu, 2021(41)	Serious	Moderate	Low	Low	Critical	Low	Low	Serious
Mair, 2021(42)	Low	Serious	Low	No information	Low	Low	Low	Moderate
Mathews, 2022(43)	Serious	Moderate	Low	No information	Low	Low	Low	Serious
McNamara, 2021(45)	Moderate	Low	Low	Low	Low	Low	Low	Moderate
McCoy, 2022(44)	Critical	Low	Low	Low	Low	Low	Low	Serious
Metcalfe, 2023(46)	Serious	Low	Moderate	No information	Low	Moderate	Low	Serious
Minsky, 2021(47)	Serious	Low	Low	Low	Serious	Low	Low	Serious
Offiah, 2022(49)	Critical	No information	Low	Serious	Low	Low	Low	Serious
Ostberg, 2022(50)	Low	Serious	Low	Low	Low	Low	Low	Serious
Parise, 2021(51)	Serious	Low	Low	Low	Serious	Low	Low	Serious
Phillips, 2021(52)	Moderate	Low	Serious	Low	Low	Moderate	Low	Serious
Piga, 2022(53)	Low	Serious	Low	No information	Low	Serious	Low	Serious
Pinsker, 2021(54)	Serious	Low	Low	Low	Low	Low	Moderate	Serious

Author, year	Domain 1: Confounding	Domain 2: Patient Selection	Domain 3: Classifying Interventions	Domain 4: Deviations from intended interventions	Domain 5: Missing data	Domain 6: Measurement of outcomes	Domain 7: Selection of reported results	Overall Assessment
Reddy, 2021(56)	Critical	Low	Low	Low	No informatio n	Low	Low	Serious
Rene, 2022(57)	Low	Moderate	Low	No information	Low	Low	Low	Serious
Ripp, 2022(58)	Low	Low	Low	Low	Low	Low	Low	Low
Rowe, 2021(59)	Moderate	Low	Serious	Low	Low	Low	Low	Serious
Ryskina, 2021(60)	Moderate	Low	Moderate	Low	Low	Low	Low	Moderate
Severino, 2022(61)	Low	Moderate	Serious	No information	Moderate	Low	Low	Serious
Sevilis, 2022(62)	Critical	Low	Low	Low	Low	Low	Serious	Critical
Shah, 2022(63)	Moderate	Low	Low	No information	Moderate	Serious	Low	Moderate
Sharma, 2020(64)	Serious	Serious	Low	Low	Low	Moderate	Low	Serious
Sohail, 2023(65)	Low	Moderate	Low	No information	Moderate	Moderate	Low	Moderate
Sun, 2022(66)	Moderate	Low	Low	No information	Moderate	Low	Moderate	Moderate
Szigety, 2022(67)	Serious	Moderate	Low	No information	No informatio n	Low	Low	Serious
Tarn, 2021(68)	Serious	Low	Low	Low	Low	Low	Low	Serious
Tchang, 2022(69)	Critical	Low	Low	Low	Moderate	Low	Low	Serious
Wabe, 2022(70)	Low	Low	Low	Low	Low	Low	Low	Low
Walker, 2023(71)	Low	Low	Low	No information	Critical	Low	Serious	Serious
Watson, 2021(72)	Critical	Low	Low	Low	Low	Low	Low	Serious
Ye, 2022(73)	Low	Low	Low	Low	Low	Low	Low	Low
Zayde, 2021(74)	Serious	Low	Low	Low	Low	Low	Low	Serious
Zhao, 2021(75)	Low	Moderate	Low	Low	Low	Low	Low	Moderate
Zhu, 2021(76)	Serious	Moderate	Low	Low	Moderate	Moderate	Low	Serious
Zimmerman, 2021(77)	Serious	Low	Low	Low	Moderate	Serious	Low	Serious

Supplementary Table 7-B. Risk of Bias Assessment for Randomized Controlled Trials.

Author, year	Domain 1: Randomization process	Domain 2: Deviations intended interventions (effect of assignment to intervention)	Domain 2: Deviations intended interventions (effect of adhering to intervention)	Domain 3: Missing outcome data	Domain 4: Measurement of the outcome	Domain 5: Selection of the reported result	Overall Assessment
Griebeler, 2022(27)	High risk	Low risk	Low risk	Some concerns	Low risk	Low risk	High risk
Mossack, 2022(48)	Low risk	Low risk	High risk	Low risk	Some concerns	Low risk	High risk
Prato, 2022(55)	Low risk	Some concerns	High risk	Low risk	High risk	Low risk	High risk

Supplementary Table 8. Summary of Findings for Outcomes of Care Among Patients Receiving Telehealth Versus In-person Care During COVID-19.

Clinical area	Number of Studies and Sample Size (N)	The Direction of Findings (Sample Size)			Conclusion*	Strength of Evidence
		Favors In-person	No Difference/ Unclear	Favors Telehealth		
**Healthcare Utilization Outcomes**						
Emergency Department Visits (N=14 Studies)						
General medical care, Adults	1 study (16,987)<br>- Cohort<br>1 study(63)<br>(N=16,987)	Cohort<br>1 study(63)<br>(N=16,987)			For adult patients who receive care for general medical conditions, those who receive an initial telehealth visit have higher rates of ED visits compared with those who receive in-person care.	High<br>Direct<br>Imprecise<br>Unknown Consistency<br>Undetected<br>Overall: Low
General medical care, Child	1 study (1,769)<br>- Cohort<br>Cohort<br>1 study(30) (N=1,769)		Cohort<br>1 study(30)<br>(N=1,769)		For children who receive care for general medical conditions, those who receive an initial telehealth visit have the same rates of ED visits compared with those who receive in-person care.	High<br>Direct<br>Imprecise<br>Unknown Consistency<br>Undetected<br>Overall: Low
General medical care, All ages	2 studies (N=608,878)<br>- Cohort<br>2 studies (28, 52)<br>(N=608,878)	Cohort<br>2 studies (28, 52)<br>(N=608,878)		Cohort<br>1 study(28)<br>(N=607,573)	For patients of all ages who receive care for general medical conditions, those who receive an initial telehealth visit for an acute condition may have higher rates of ED visits compared with those who receive in-person care and those who receive an initial telehealth visit for a chronic condition may have lower rates of ED visits compared with those who receive in-person care.	Medium<br>Direct<br>Precise<br>Inconsistent<br>Undetected<br>Overall: Moderate
Care for specific conditions, COVID-19	3 studies (N=5,462)<br>- Cohort<br>3 studies(8, 12, 36)<br>(N=5,462)	Cohort<br>1 study(12)<br>(N=4,384)		Cohort<br>2 studies(8, 36) (N=1,078)	For patients who receive specialized COVID-19 care, those who receive an initial telehealth visit may have higher ED visit rates compared with those who receive in-person care.	High<br>Direct<br>Precise<br>Inconsistent<br>Undetected<br>Overall: Low
Care for specific conditions, Pregnancy/prenatal/gynecologic al care	1 study (N=287)<br>- Cohort<br>1 study(32) (N=287)	Cohort<br>1 study(32)<br>(N=287)			For women who receive specialized pregnancy/prenatal/gynecological care, those who receive an initial telehealth visit may have higher ED visit rates compared with those who receive in-person.	High<br>Direct<br>Imprecise<br>Unknown consistency<br>Undetected<br>Overall: Low
Care for specific conditions, Other conditions	6 studies (N=11,546)<br>- Cohort<br>5 studies (3, 17, 23, 56, 71, 72)<br>(N=41,496)<br>- Cross-sectional	Cross-sectional<br>1 study(72)<br>(N=1,724)		Cohort<br>5 studies (3, 17, 23, 56, 71)<br>(N=41,496)	For patients who receive care for specific conditions (excluding COVID-19 and pregnancy/prenatal/gynecological care), those who receive an initial telehealth visit may have lower ED	High<br>Direct<br>Precise<br>Consistent<br>Suspected<br>Overall: Low

Clinical area	Number of Studies and Sample Size (N)	The Direction of Findings (Sample Size)			Conclusion*	Strength of Evidence
		Favors In-person	No Difference/ Unclear	Favors Telehealth		
	1 study(72) (N=1,724)				visit rates compared with those who receive in-person.	
General behavioral/Mental health	No studies					
Hospitalization (N=20 Studies)						
General medical care, Adult	3 studies (N=17,517)<br>- Cohort<br>3 studies (60, 63, 77) (N=34,504)	Cohort<br>1 study(63) (N=16,987)		Cohort<br>2 studies(60, 77) (N=17,517)	For adult patients who receive care for general medical conditions, hospitalization rates may be similar for those receiving an initial telehealth visit compared with those who receive an initial in-person visit.	High<br>Direct<br>Precise<br>Inconsistent<br>Undetected<br>Overall: Moderate
General medical care, Child	No studies					
General medical care, All ages	2 studies (N=608,878)<br>- Cohort<br>2 studies (28, 52) (N=608,878)	Cohort<br>1 study(28) (N=607,573)		Cohort<br>2 studies (28, 52) (N=608,878)	For patients of all ages who receive care for general medical conditions, those who receive an initial telehealth visit for an acute condition may have higher hospitalization rates compared with those who receive in-person care and those who receive an initial telehealth visit for a chronic condition may have lower hospitalization rates compared with those who receive in-person care.	Medium<br>Direct<br>Precise<br>Inconsistent<br>Undetected<br>Overall: Moderate
Care for specific conditions, COVID-19	2 studies (N=4,677)<br>- Cohort<br>2 studies (12, 36) (N=4,677)	Cohort<br>1 study(12) (N=4,384)		Cohort<br>1 study(36) (N=293)	For patients who receive specialized COVID-19 care, those who receive an initial telehealth visit may have higher hospitalization rates compared with those who receive in-person care.	High<br>Direct<br>Precise<br>Inconsistent<br>Undetected<br>Overall: Low
Care for specific conditions, Pregnancy/prenatal/gynecological care	2 studies (N=14,186)<br>- Cohort<br>2 studies (5, 19) (N=14,186)	Cohort<br>2 studies (5, 19) (N=14,186)			For women who receive specialized pregnancy/prenatal/gynecological care, those who receive an initial telehealth visit may have slightly higher hospitalization rates compared with those who receive in-person care.	High<br>Direct<br>Precise<br>Consistent<br>Undetected<br>Overall: Low
Care for specific conditions, Other conditions	11 studies (N=73,358)<br>- Cohort<br>10 studies (3, 17, 18, 50, 61, 62, 64, 71, 75, 76) (N=71,634)<br>- Cross-sectional<br>1 study(72) (N=1,724)	Cohort<br>3 studies (50, 62, 75) (N=27,323)<br>Cross-sectional<br>1 study(72) (N=1,724)		Cohort<br>7 studies(3, 17, 18, 61, 64, 71, 76) (N=44,311)	For patients who receive care for specific conditions (excluding COVID-19 and pregnancy/prenatal/gynecological care), those who receive an initial telehealth visit may have lower hospitalization rates compared with those who receive in-person care.	High<br>Direct<br>Precise<br>Inconsistent<br>Suspected<br>Overall: Low

Clinical area	Number of Studies and Sample Size (N)	The Direction of Findings (Sample Size)			Conclusion*	Strength of Evidence
		Favors In-person	No Difference/ Unclear	Favors Telehealth		
General Behavioral/Mental Health	No studies					
Readmission (N=4 Studies)						
General medical care, Adult	No studies					
General medical care, Child	No studies					
General medical care, All ages	No studies					
Care for specific conditions, COVID-19	2 studies (N=992)<br>- Cohort<br>2 studies (8, 11) (N=992)			Cohort<br>2 studies(8, 11) (N=992)	For patients who receive specialized COVID-19 care, those who receive an initial telehealth visit may have similar readmission rates compared with those who receive in-person care.	High<br>Direct<br>Precise<br>Consistent<br>Undetected<br>Overall: Low
Care for specific conditions, Pregnancy/prenatal/gynecologic al care	1 study (N=473)<br>- Cohort<br>1 study(33) (N=473)			Cohort<br>1 study(33) (N=473)	For women who receive pregnancy/prenatal/gynecological care, readmission rates may be similar for those receiving telehealth compared with those who receive in-person care.	Medium<br>Direct<br>Precise<br>Unknown consistency<br>Undetected<br>Overall: Low
Care for specific conditions, Other conditions	1 study (N= 146)<br>- Cohort<br>1 study(29) (N= 146)			1 study (N= 146)<br>- Cohort<br>1 study(29) (N= 146)	For patients who receive care for specific conditions (excluding COVID-19 and pregnancy/prenatal/gynecological care), those who receive an initial telehealth visit may have lower readmission rates compared with those who receive in-person care.	High<br>Direct<br>Precise<br>Unknown consistency<br>Undetected<br>Overall: Low
General behavioral/Mental health	No studies					
**Clinical Outcomes**						
Mortality (N=6 Studies)						
General medical care, Adult	No studies					
General medical care, Child	No studies					
General medical care, All ages	No studies					
Care for specific conditions, COVID-19	No studies					
Care for specific conditions, Pregnancy/prenatal/gynecologic al care	2 studies (N=53,721)<br>- Cohort<br>1 study(5) (N=1,579)<br>- Cross-sectional<br>1 study(4) (N=52,142)	Cohort<br>1 study(5) (N=1,579)	Cross-sectional<br>1 study(4) (N=52,142)		For women who receive specialized pregnancy/prenatal/gynecological care, those who receive an initial telehealth visit may have similar mortality rates compared with those who receive in-person care.	High<br>Direct<br>Precise<br>Consistent<br>Undetected<br>Overall: Low
Care for specific conditions, Other conditions	4 studies (N=2,193)<br>- Cohort<br>3 studies(3, 61, 75) (N=469)			Cohort<br>3 studies(3, 61, 75) (N=469)	For patients who receive care for specific conditions (excluding COVID-19 and pregnancy/prenatal/gynecological	High<br>Direct<br>Precise<br>Consistent

Clinical area	Number of Studies and Sample Size (N)	The Direction of Findings (Sample Size)			Conclusion*	Strength of Evidence
		Favors In-person	No Difference/ Unclear	Favors Telehealth		
	- Cross-sectional<br>1 study(72) (N=1,724)			Cross-sectional<br>1 study(72)<br>(N=1,724)	care), those who receive an initial telehealth visit may have lower mortality rates compared with those who receive in-person care.	Undetected<br>Overall: Low
General behavioral/Mental health	No studies					
Patient-Reported Outcome (N=8 Studies)						
General medical care, Adult	No studies					
General medical care, Child	No studies					
General medical care, All ages	No studies					
Care for specific conditions, COVID-19	No studies					
Care for specific conditions, Pregnancy/prenatal/gynecological care	2 studies (N=2,005)<br>- Cohort<br>2 studies(5, 10)<br>(N=2,005)	Cohort<br>1 study(5)<br>(N=1,579)		Cohort<br>1 study<br>(10)<br>(N=426)	For women who receive specialized pregnancy/prenatal/gynecological care, those who receive an initial telehealth visit may have worse patient-reported outcomes compared with those who receive in-person care.	High<br>Direct<br>Precise<br>Inconsistent<br>Undetected<br>Overall: Low
Care for specific conditions, Other conditions	1 study (N=279)<br>- Cohort<br>1 study(47) (N=279)			Cohort<br>1 study(47)<br>(N=279)	For patients who receive care for specific conditions (excluding COVID-19 and pregnancy/prenatal/gynecological care), those who receive an initial telehealth visit may have better patient-reported outcomes compared with those who receive in-person care.	High<br>Direct<br>Precise<br>Unknown consistency<br>Undetected<br>Overall: Low
General behavioral/Mental health	5 studies (N=1,079)<br>- Cohort<br>5 studies(31, 37, 41, 57, 74)<br>(N=1,079)	Cohort<br>1 study(37)<br>(N=93)	Cohort<br>1 study(57)<br>(N=338)	Cohort<br>3 studies(31, 41, 74)<br>(N=648)	For patients receiving care for general behavioral and mental health conditions, those who receive an initial telehealth visit may have better patient-reported outcomes compared with those who receive in-person care.	High<br>Direct<br>Precise<br>Inconsistent<br>Undetected<br>Overall: Low
Condition-Specific Clinical Outcomes (N=15 Studies)						
General medical care, Adult	1 study (N=70)<br>- RCT<br>1 study(27) (N=70)	1 study<br>(N=70)<br>- RCT<br>1 study(27)<br>(N=70)			For adult patients who receive care for general medical conditions, those who receive an initial telehealth visit may have worse clinical outcomes compared with those who receive in-person care.	High<br>Direct<br>Precise<br>Unknown consistency<br>Undetected<br>Overall: Low
General medical care, Child	No studies					
General medical care, All ages	No studies					

Clinical area	Number of Studies and Sample Size (N)	The Direction of Findings (Sample Size)			Conclusion*	Strength of Evidence
		Favors In-person	No Difference/ Unclear	Favors Telehealth		
Care for specific conditions, COVID-19	No studies					
Care for specific conditions, Pregnancy/prenatal/gynecological care	4 studies (N=56,100)<br>- Cohort<br>2 studies(5, 14)<br>(N=2,082)<br>- Cross-sectional<br>2 studies(4, 25)<br>(N=54,018)	Cohort<br>1 study(5)<br>(N=1,579)<br>Cross-sectional<br>1 study(25)<br>(N=1,876)	- Cohort<br>1 study(14)<br>(N=503)	Cross-sectional<br>1 study(4)<br>(N=52,142)	For women who receive specialized pregnancy/prenatal/gynecological care, those who receive an initial telehealth visit may have worse condition-specific clinical outcomes compared with those who receive in-person care.	High<br>Direct<br>Imprecise<br>Inconsistent<br>Undetected<br>Overall: Low
Care for specific conditions, Other conditions	8 studies (N=73,581)<br>- Cohort<br>8 studies(6, 17, 21, 42, 47, 69, 71, 73)<br>(N=73,581)	Cohort<br>4 studies(6, 17, 71, 73)<br>(N=72,390)		Cohort<br>4 studies(21, 42, 47, 69)<br>(N=1,191)	For patients who receive care for specific conditions (excluding COVID-19 and pregnancy/prenatal/gynecological care), those who receive an initial telehealth visit may have worse condition-specific clinical outcomes compared with those who receive in-person care (SOE: Low)	High<br>Direct<br>Precise<br>Inconsistent<br>Undetected<br>Overall: Low
General behavioral/Mental health	2 studies (N=133)<br>- Cohort<br>1 study(37) (N=93)<br><br>- RCT<br>1 study(55) (N=40)		- RCT<br>1 study(55)<br>(N=40)	Cohort<br>1 study<br>Levinson, 2021(37)<br>(N=93)	For patients receiving care for general behavioral and mental health conditions, those who receive an initial telehealth visit may have better condition-specific clinical outcomes compared with those who receive in-person care.	High<br>Direct<br>Precise<br>Inconsistent<br>Undetected<br>Overall: Low
Adverse Events (N=7 Studies)						
General medical care, Adult	1 study (N=492)<br>- Cohort<br>1 study(45) (N=492)			Cohort<br>1 study(45)<br>(N=492)	For adult patients receiving general medical conditions, patients who receive an initial telehealth visit may have slightly lower adverse event rates compared with those who receive in-person care.	High<br>Direct<br>Precise<br>Unknown consistency<br>Undetected<br>Overall: Low
General medical care, Child	No studies					
General medical care, All ages	No studies					
Care for specific conditions, COVID-19	No studies					
Care for specific conditions, Pregnancy/prenatal/gynecological care	3 studies (N=65,036)<br>- Cohort<br>3 studies(4, 19, 32)<br>(N=65,036)	Cohort<br>1 study(32)<br>(N=287)		Cohort<br>2 (4, 19)<br>(N=64,749)	For women who receive specialized pregnancy/prenatal/gynecological care, those who receive an initial telehealth visit may have slightly lower adverse event rates compared with those who receive in-person care.	High<br>Direct<br>Precise<br>Consistent<br>Undetected<br>Overall: Low
Care for specific conditions, Other conditions	2 studies (N=23,452)<br>- Cohort			Cohort<br>2 studies(54, 61) (N=23,452)	For patients who receive care for specific conditions (excluding COVID-19 and	High<br>Direct<br>Precise

Clinical area	Number of Studies and Sample Size (N)	The Direction of Findings (Sample Size)			Conclusion*	Strength of Evidence
		Favors In-person	No Difference/ Unclear	Favors Telehealth		
	2 studies(54, 61) (N=23,452)				pregnancy/prenatal/gynecological care), those who receive an initial telehealth visit may have a slightly lower adverse events rate compared with those who receive in-person care.	Consistent<br>Undetected<br>Overall: Low
General behavioral/Mental health	1 study (N=74)<br>- Cohort<br>1 study(20) (N=74)		Cohort<br>1 study(20) (N=74)	NA	For patients receiving care for general behavioral and mental health conditions, those who receive an initial telehealth visit may have similar rates of adverse events compared with those who receive in-person care.	High<br>Direct<br>Imprecise<br>Unknown consistency<br>Undetected<br>Overall: Low
**Process Outcomes**						
Missed Visits (N=14 Studies)						
General medical care, Adult	2 studies (N=331,638)<br>- Cohort<br>1 study(43) (N=2,177)<br><br>- Cross-sectional<br>1 study (13) (N=329,461)	Cross-sectional<br>1 study(13) (N=329,461)		Cohort<br>1 study(43) (N=2,177)	For adult patients receiving general medical conditions, patients who receive an initial telehealth visit may have higher rates of missed visits compared with those who receive in-person care.	High<br>Direct<br>Precise<br>Inconsistent<br>Undetected<br>Overall: Low
General medical care, Child	No Studies					
General medical care, All ages	1 study (N=278,171)<br>- Cohort<br>1 study(2) (N=278,171)			Cohort<br>1 study(2) (N=278,171)	For patients of all ages receiving general medical conditions, patients who receive an initial telehealth visit may have lower rates of missed visits compared with those who receive in-person care.	High<br>Direct<br>Precise<br>Unknown consistency<br>Undetected<br>Overall: Low
Care for specific conditions, COVID-19	No Studies					
Care for specific conditions, Pregnancy/prenatal/gynecologic al care	3 studies (N=4,243)<br>- Cohort<br>3 studies(1, 5, 14) (N=4,243)			Cohort<br>3 studies(1, 5, 14) (N=4,243)	For women who receive specialized pregnancy/prenatal/gynecological care, those who receive an initial telehealth visit may have lower missed visit rates compared with those who receive in-person care.	High<br>Direct<br>Precise<br>Consistent<br>Undetected<br>Overall: Low
Care for specific conditions, Other conditions	6 studies (N=34,280)<br>- Cohort<br>5 studies(34, 65, 66, 72, 76) (N=33,702)<br>- Cross-sectional<br>1 study(9) (N=578)	Cohort<br>2 studies(34, 72) (N=2,694)		Cohort<br>3 studies(65, 66, 76) (N=31,008)<br>Cross-sectional<br>1 study(9) (N=578)	For patients who receive care for specific conditions (excluding COVID-19 and pregnancy/prenatal/gynecological care), those who receive an initial telehealth visit may have lower missed visit rates compared with those who receive in-person care.	High<br>Direct<br>Precise<br>Consistent<br>Undetected<br>Overall: Low

Clinical area	Number of Studies and Sample Size (N)	The Direction of Findings (Sample Size)			Conclusion*	Strength of Evidence
		Favors In-person	No Difference/ Unclear	Favors Telehealth		
General behavioral/Mental health	2 studies (N=350)<br>- Cohort<br>2 studies(57, 74) (N=350)	Cohort<br>2 studies(57, 74) (N=350)			For patients receiving care for general behavioral and mental health conditions, those who receive an initial telehealth visit may have higher rates of missed visits compared with those who receive in-person care.	High<br>Direct<br>Imprecise<br>Consistent<br>Undetected<br>Overall: Low
Case Resolution/ Duplication of Services (N=14 Studies)						
General medical care, Adult	No studies					
General medical care, Child	1 study (N=1,769)<br>- Cohort<br>1 study(30) (N=1,769)		Cohort<br>1 study(30) (N=1,769)		For children who receive care for general medical conditions, those who receive an initial telehealth visit have the same rates of case resolution compared with those who receive in-person care.	High<br>Direct<br>Imprecise<br>Unknown Consistency<br>Undetected<br>Overall: Low
General medical care, All ages	2 studies (N=607,769)<br>- Cohort<br>1 study(28) (N=607,573)<br>- Cross-sectional<br>1 study(68)(N=196)	Cohort<br>1 study(28) (N=607,573)<br>Cross-sectional<br>1 study(68) (N=196)		Cohort<br>1 study(28) (N=505,224)	For patients of all ages who receive care for general medical conditions, those who receive an initial telehealth visit for an acute condition may have higher rates of follow-up visits compared with those who receive in-person care and those who receive an initial telehealth visit for a chronic condition may have lower rates of follow-up visits compared with those who receive in-person care.	High<br>Direct<br>Precise<br>Inconsistent<br>Undetected<br>Overall: Moderate
Care for specific conditions, COVID-19	1 study (N=285)<br>- Cohort<br>1 study(11) (N=285)			Cohort<br>1 study(11) (N=285)		High<br>Direct<br>Precise<br>Unknown Consistency<br>Undetected<br>Overall: Insufficient
Care for specific conditions, Pregnancy/prenatal/gynecological care	1 study (N=218)<br>- Cohort<br>1 study(32) (N=218)			Cohort<br>1 study(32) (N=218)		High<br>Direct<br>Precise<br>Unknown Consistency<br>Undetected<br>Overall: Insufficient
Care for specific conditions, Other conditions	9 studies (N=8,313)<br>- Cohort<br>8 studies(21, 35, 38, 39, 46, 49, 59, 76) (N=8,265)<br>- RCT<br>1 study(48) (N=48)	Cohort<br>4 studies(39, 46, 59, 76) (N=6,098)<br>RCT<br>1 study(48) (N=48)		Cohort<br>4 studies(21, 35, 38, 49) (N=2,167)	For patients who receive care for specific conditions (excluding COVID-19 and pregnancy/prenatal/gynecological care), those who receive an initial telehealth visit may have higher rates of case resolution and a lower rate of duplicated services	High<br>Direct<br>Precise<br>Inconsistent<br>Undetected<br>Overall: Low

Clinical area	Number of Studies and Sample Size (N)	The Direction of Findings (Sample Size)			Conclusion*	Strength of Evidence
		Favors In-person	No Difference/ Unclear	Favors Telehealth		
					compared with those who receive in-person care.	
General behavioral/Mental health	No studies					
Change in Therapy/ Medication (N=11 Studies)						
General medical care, Adult	2 studies (N=13,608,708)<br>- Cohort<br>2 studies(45, 70)<br>(N=13,608,708)	Cohort<br>2 studies(45, 70)<br>(N=13,608,708)			For adult patients receiving general medical conditions, patients who receive an initial in-person visit may have higher rates of change in therapy/medication compared with those who receive telehealth.	High<br>Direct<br>Precise<br>Consistent<br>Undetected<br>Overall: Moderate
General medical care, Child	No studies					
General medical care, All ages	No studies					
Care for specific conditions, COVID-19	No studies					
Care for specific conditions, Pregnancy/prenatal/gynecological care	No studies					
Care for specific conditions, Other conditions	9 studies (N=8,104)<br>- Cohort<br>8 studies(15, 39, 42, 49, 53, 61, 64, 76)<br>(N=8,056)<br><br>- RCT<br>1 study(48) (N=48)	Cohort<br>3 studies(15, 42, 76)(N=4,254)<br><br>RCT<br>1 study(48)<br>(N=48)		Cohort<br>5 studies(39, 49, 53, 61, 64)<br>(N=3,802)	For patients who receive care for specific conditions (excluding COVID-19 and pregnancy/prenatal/gynecological care), those who receive an initial telehealth visit may have lower rates of change in therapy/medication compared with those who receive in-person care.	High<br>Direct<br>Precise<br>Consistent<br>Undetected<br>Overall: Low
General behavioral/Mental health	No studies					
Treatment/ Medication Adherence (N=13 Studies)						
General medical care, Adult	3 studies (N=960)<br>- Cohort<br>2 studies(45, 77)<br>(N=906)<br>- RCT<br>1 study(27)<br>(N=54)		Cohort<br>1 study(45)<br>(N=492)	Cohort<br>1 study(77)<br>(N=414)<br><br>RCT<br>1 study(27)<br>(N=54)	For patients of all ages who receive care for general medical conditions, those who receive an initial telehealth visit may have higher rates of therapy/medication adherence compared with those who receive in-person care.	High<br>Direct<br>Precise<br>Consistent<br>Undetected<br>Overall: Low
General medical care, Child	No studies					
General medical care, All ages	No studies					
Care for specific conditions, COVID-19	No studies					
Care for specific conditions, Pregnancy/prenatal/gynecological care	No studies					

Clinical area	Number of Studies and Sample Size (N)	The Direction of Findings (Sample Size)			Conclusion*	Strength of Evidence
		Favors In-person	No Difference/ Unclear	Favors Telehealth		
Care for specific conditions, Other conditions	5 studies (N=144,426)<br>- Cohort<br>3 studies(7, 17, 26) (N=144,202)<br>- Cross-sectional<br>1 study(44) (N=176)<br>- RCT<br>1 study(48) (N=48)	Cohort<br>1 study(7)<br>(N= 127,444)	Cohort<br>1 study(26)<br>(N=270)	Cohort<br>1 study(17)<br>(N=3,744<br>Cross-sectional<br>1 study(44)<br>(N=176)<br>RCT<br>1 study(48)<br>(N=48)	For patients who receive care for specific conditions (excluding COVID-19 and pregnancy/prenatal/gynecological care), those who receive an initial telehealth visit may have lower rates of therapy/medication adherence compared with those who receive in-person care.	High<br>Direct<br>Precise<br>Consistent<br>Undetected<br>Overall: Low
General behavioral/Mental health	5 studies (N=6,494)<br>- Cohort<br>4 studies(16, 20, 24, 58) (N=2,156)<br>- Cross-sectional<br>1 study(22) (N=4,338)			Cohort<br>4 studies(16, 20, 24, 58)<br>(N=2,156)<br>Cross-sectional<br>1 study(22)<br>(N=4,338)	For patients receiving care for general behavioral and mental health conditions, those who receive an initial telehealth visit may have higher rates of therapy/medication adherence compared with those who receive in-person care.	High<br>Direct<br>Precise<br>Consistent<br>Undetected<br>Overall: Low
Up-to-Date Laboratory and Paraclinical Assessment (N=9 Studies)						
General medical care, Adult	No studies					
General medical care, Child	1 study (N=1,769)<br>- Cohort<br>1 study(30) (N=1,769)	1 study<br>(N=1,769)<br>- Cohort<br>1 study(30)<br>(N=1,769)			For children who receive care for general medical conditions, those who receive an initial telehealth visit have lower rates of up-to-date labs and paraclinical assessment compared with those who receive in-person care.	High<br>Direct<br>Precise<br>Unknown Consistency<br>Undetected<br>Overall: Low
General medical care, All ages	No studies					
Care for specific conditions, COVID-19	No studies					
Care for specific conditions, Pregnancy/prenatal/gynecological care	1 study (N=104)<br>- Cohort<br>1 study(5) (N=104)	Cohort<br>1 study(5)<br>(N=104)			For women receiving care for specialized care for pregnancy/prenatal/ gynecological care, those who receive an initial telehealth visit may have similar rates of up-to-date labs and paraclinical assessment compared with those who receive in-person care.	Medium<br>Direct<br>Imprecise<br>Unknown Consistency<br>Undetected<br>Overall: Low
Care for specific conditions, Other conditions	7 studies (N=14,378)<br>- Cohort<br>6 studies (15, 39, 41, 51, 56, 67) (N=13,468)<br>- Cross-sectional<br>1 study(50) (N=910)	Cohort<br>5 studies (15, 39, 41, 56, 67)<br>(N=13,259)	Cross-sectional<br>1 study(50)<br>(N=910)	Cohort<br>1 study(51);<br>(N=209)	For patients receiving care for specific conditions (excluding COVID-19 and pregnancy/prenatal/ gynecological care), those who receive an initial telehealth visit may have lower rates of up-to-date labs and paraclinical assessment	High<br>Direct<br>Precise<br>Inconsistent<br>Undetected<br>Overall: Low

Clinical area	Number of Studies and Sample Size (N)	The Direction of Findings (Sample Size)			Conclusion*	Strength of Evidence
		Favors In-person	No Difference/ Unclear	Favors Telehealth		
					compared with those who receive in-person care.	
General behavioral/Mental health	No studies					

ED=emergency department; NA=not applicable/no studies; SOE =strength of evidence
* The strength of evidence domains included in the assessment (as listed in descending order) were study limitations (reported as low, medium, high), directness (whether the evidence links the interventions directly to health outcomes), precision (the degree of certainty surrounding an effect estimate concerning a given outcome), consistency (the degree to which reported effect sizes appear to have the same direction of effect), and reporting bias (indicates selective publication of results, which results in the miss-representation of the true effect). After the assessment of each domain, the strength of evidence received a single grade of high, moderate, low, or insufficient.

Supplementary Figure 1. Health Outcomes for Patients with an Initial Telehealth versus In-person Visit by Outcome Category and Clinical Area.*

A. Healthcare Utilization

Clinical area	Author, year	Outcome	Total N	OR (95% CI)
**ED/ER visits**				
COVID-19	Borgen, 2021	ED or Observation Unit	785	0.36 (0.23, 0.58)
COVID-19	Korycinski, 2022	ED visit	293	0.56 (0.27, 1.15)
General medical care - Adults	Shah, 2022	ED visit	16987	1.23 (1.09, 1.39)
General medical care -All Ages	Phillips, 2021	ED visit	1305	1.24 (0.73, 2.11)
General medical care -All Ages	Hatef, 2022	ED followup	607573	1.11 (1.06, 1.16)
General medical care -All Ages	Hatef, 2022	ED followup	505224	0.96 (0.92, 1.01)
Pregnancy/Prenatal/OBGYN	Kerestes, 2021	ER visit	163	2.83 (0.50, 15.92)
Specific condition - Other	Gaetani, 2021	Emergency Room Visit or Hospitalization	90	0.77 (0.28, 2.10)
Specific condition - Other	Reddy, 2021	Emergency center visit	1576	0.27 (0.11, 0.62)
Specific condition - Other	Watson, 2021	ED presentation	1724	2.10 (0.54, 8.13)
Specific condition - Other	Walker, 2023	ED visit	31654	0.71 (0.67, 0.74)
**Hospitalization**				
COVID-19	Casariego-Vales, 2021	Hospitalization after ED visit	4384	8.84 (6.60, 11.80)
COVID-19	Korycinski, 2022	Hospital admission	293	0.65 (0.23, 1.84)
General medical - Adults	Shah, 2022	Hospitalization	16987	1.31 (1.09, 1.58)
General medical care -All Ages	Rysinka, 2021	Any Hospitalization	NR	0.72 (0.57, 0.90)
General medical care -All Ages	Phillips, 2021	Hospital admission	1305	0.64 (0.31, 1.35)
General medical care -All Ages	Hatef, 2022	Acute ambulatory care, hospitalization followup	607573	1.03 (0.98, 1.08)
General medica care -All Agesl	Hatef, 2022	Choleric ambulatory care, hospitalization followup	505224	0.94 (0.90, 0.99)
Pregnancy/Prenatal/OBGYN	Duryea, 2021	Full Term NICU Admission	12607	1.00 (0.75, 1.33)
Pregnancy/Prenatal/OBGYN	Arias, 2022	Intensive care nursery	1579	1.12 (0.84, 1.49)
Specific condition - Other	Zhu, 2021	Planned Admission or Procedure	1493	0.38 (0.21, 0.71)
Specific condition - Other	Zhao, 2021	Hospitalization for Heart Failure	82	4.76 (0.22, 102.26)
Specific condition - Other	Ostberg, 2022	Admit to in-patient	910	1.08 (0.63, 1.86)
Specific condition - Other	D'Anna, 2022	Admission to hospital	316	0.88 (0.15, 5.34)
Specific condition - Other	Sevilis, 2022	Inpatient thrombolytics	26331	1.46 (1.04, 2.04)
Specific condition - Other	Watson, 2021	Hospitalization within 24hrs	1724	1.10 (0.58, 2.06)
Specific condition - Other	Severino, 2022	Cardiovascular hospitalization	184	0.69 (0.32, 1.48)
Specific condition - Other	Walker, 2023	In-patient admissions	31654	0.82 (0.78, 0.86)
**Readmission**				
COVID-19	Borgen, 2021	Hospital Readmission	707	0.79 (0.27, 2.32)
COVID-19	Carlberg, 2020	Admitted upon return, within72 hours, COVID-19 related	285	0.29 (0.01, 7.07)
Pregnancy/Prenatal/OBGYN	Khosla, 2022	Readmission	473	0.98 (0.61, 1.58)

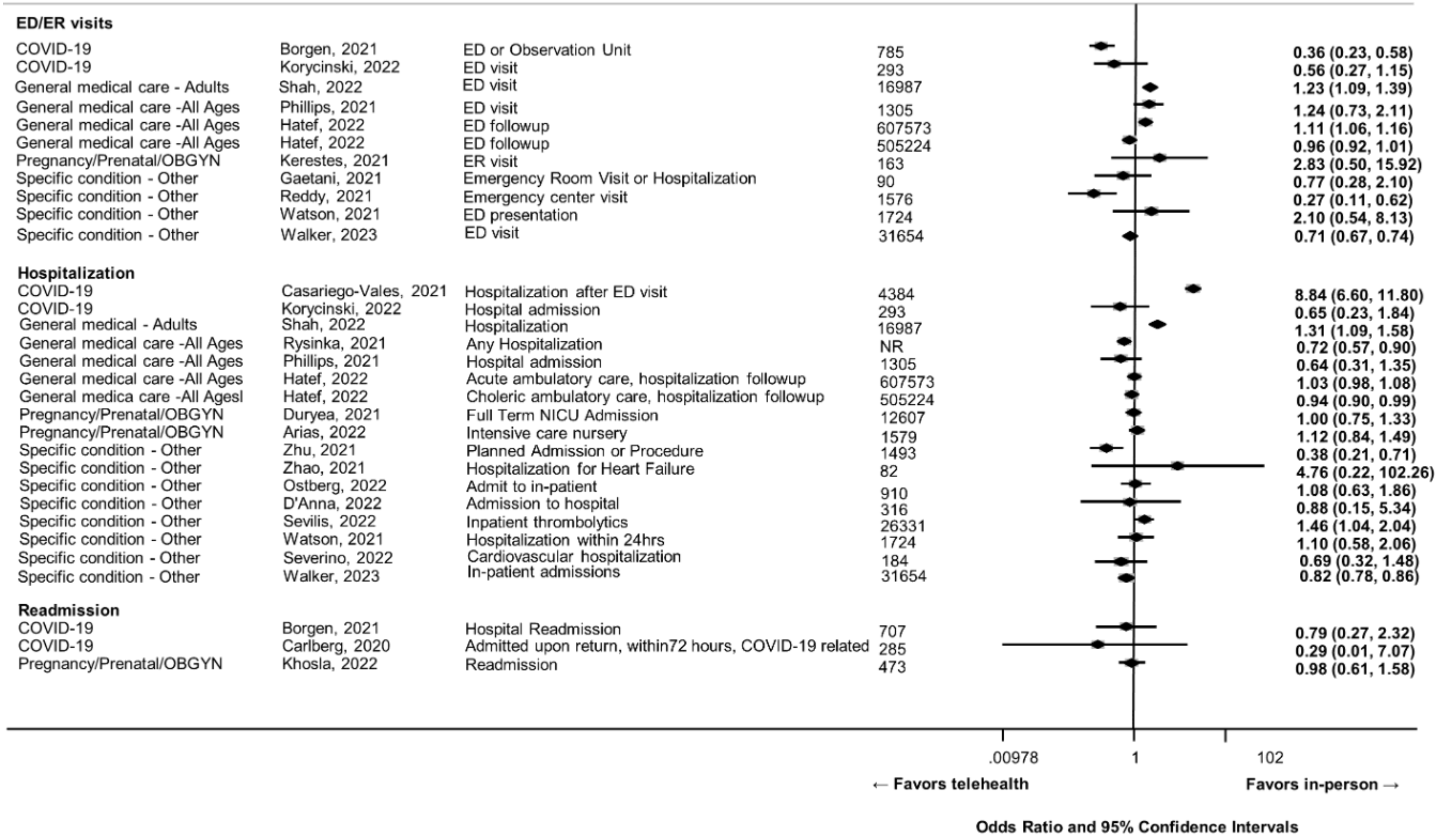

B. Clinical Outcomes

Clinical area	Author, year	Population	Outcome	Total N	OR (95% CI)
**Mortality**					
Pregnancy/Prenatal/OBGYN	Aiken, 2021	Pregnancy	Death	52142	**Zero events**
Pregnancy/Prenatal/OBGYN	Arias, 2022	Postpartum visits	Fetal death	1579	1.16 (0.51, 2.60)
Specific condition - Other	Afonso Noguueria, 2021	Cardiology	Mortality	203	0.17 (0.02, 1.28)
Specific condition - Other	Zhao, 2021	Heart Failure	Mortality/major cardiovascular event	82	0.44 (0.04, 5.06)
Specific condition - Other	Watson, 2021	Cancer patients	Mortality	1724	0.06 (0.00, 1.04)
Specific condition - Other	Severino, 2022	Heart Failure	Mortality	184	0.90 (0.36, 2.23)
**Patient reported outcomes**					
Specific condition - Other	Minsky, 2021	Overweight	Deterioration in dietary habit score	279	0.68 (0.36, 1.28)
Pregnancy/Prenatal/OBGYN	Arias, 2022	Postpartum visits	Postpartum depression screening	1579	4.61 (3.38, 6.28)
Pregnancy/Prenatal/OBGYN	Bryson, 2023	Long acting contraceptive	Any symptom reported	426	1.40 (0.94, 2.10)
**Condition specific**					
General medical - Adults	Griebeler, 2022	Overweight or obese	5% weight change at 12 weeks	70	**0.90 (0.60, 1.30)**
Pregnancy/Prenatal/OBGYN	Aiken, 2021	Pregnancy	Successful Medical Abortion	52142	1.45 (1.25, 1.67)
Pregnancy/Prenatal/OBGYN	Arias, 2022	Postpartum visits	Any breastfeeding	1579	0.90 (0.68, 1.18)
Pregnancy/Prenatal/OBGYN	Clark, 2022	Gestational diabetes	C-section	503	1.01 (0.71, 1.45)
Pregnancy/Prenatal/OBGYN	Clark, 2022	Gestational diabetes	NICU admission	503	1.34 (0.86, 2.09)
Pregnancy/Prenatal/OBGYN	Gao, 2022	Pregnant	Pre-term birth <37 weeks	1876	1.24 (0.96, 1.61)
Specific condition - Other	Fredwall, 2021	Epilepsy	In remission or had improvements	117	2.30 (0.49, 10.74)
Specific condition - Other	Mair, 2021	Rheumatology patients	Disease in remission	550	1.76 (1.13, 2.74)
Specific condition - Other	Minsky, 2021	Overweight	Likely to lose weight	279	2.79 (1.04, 7.48)
Specific condition - Other	Tchang, 2022	Overweight/Obese patients	≥5% weight loss	160	1.69 (0.90, 3.17)
Specific condition - Other	Ye, 2022	High blood pressure	Not meeting high BP quality measure	27623	2.06 (1.94, 2.18)
**Adverse events**					
Pregnancy/Prenatal/OBGYN	Duryea, 2021	Need for transfusion	Need for Transfusion	12607	1.20 (1.00, 1.44)
Pregnancy/Prenatal/OBGYN	Aiken, 2021	Pregnancy	Hemorrhage requiring transfusion	52142	0.65 (0.23, 1.78)
Pregnancy/Prenatal/OBGYN	Kerestes, 2021	Family Planning	Complication: Blood Transfusion	163	**Zero events**
Behavioral/Mental health	Fortier, 2022	U.S. Veterans	Adverse events	74	**Zero events**
Specific condition - Other	Severino, 2022	Heart Failure	Major adverse	184	0.88 (0.43, 1.79)

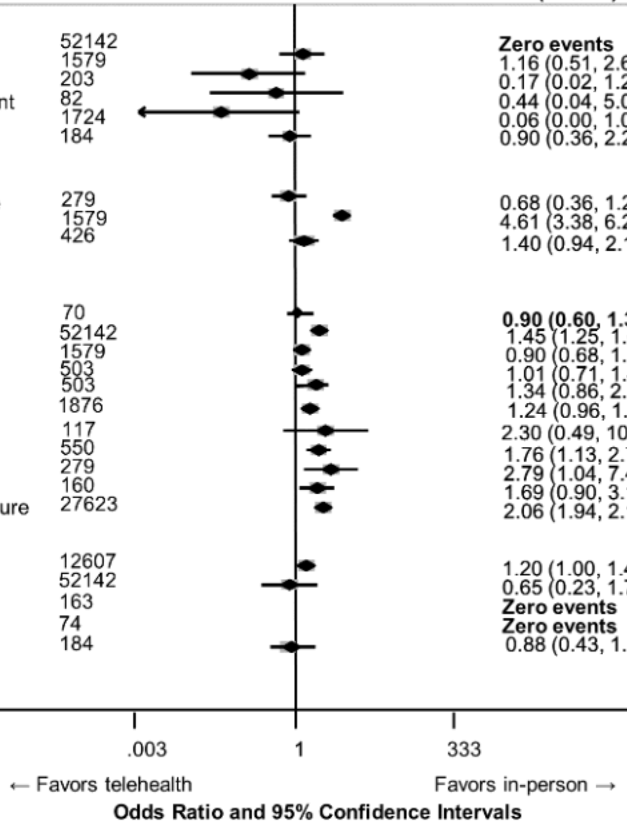

C. Process Outcomes

Clinical area	Author, year	Population	Outcome	Total N	OR (95% CI)
**Missed visits**					
General medical - Adults	Chen, 2023	Any encounter	Non-attendance	185410	1.04 (1.02, 1.07)
General medical - Adults	Chen, 2023	Any encounter	Non-attendance	52795	4.37 (2.74, 6.97)
General medical care – All ages	Adepoju, 2022	Primary care	Missed appointment	278171	0.87 (0.84, 0.89)
Pregnancy/Prenatal/OBGYN	Arias, 2022	Postpartum	Postpartum attendance rate	1579	0.54 (0.43, 0.69)
Specific condition - Other	Zhu, 2021	Rheumatology patients	Non-attendance	3040	0.57 (0.44, 0.74)
Specific condition - Other	Watson, 2021	Inflammatory bowel disease	Cancelled colonoscopies	1724	1.53 (0.76, 3.05)
Specific condition - Other	Sun, 2022	Chronic Psychotic Disorder	Appointment no show	NR	0.40 (0.29, 0.57)
Specific condition - Other	Sohail, 2023	Underwent spine surgery	Appointment no show	26458	0.61 (0.55, 0.68)
**Case Resolution**					
COVID-19	Carlberg, 2020	COVID-19	Return to healthcare	285	0.73 (0.24, 2.23)
General medical care -All Ages	Hatef, 2022	Acute ambulatory care	Any followup encounter	607573	1.44 (1.42, 1.46)
General medical care -All Ages	Hatef, 2022	Chronic ambulatory care	Any followup encounter	505224	0.94 (0.92, 0.95)
Pregnancy/Prenatal/OBGYN	Kerestes, 2021	Family Planning	Abortion completion	163	0.38 (0.07, 2.14)
Specific condition - Other	Kolb, 2021	Otolaryngology	Routine follow-up recommended	100	0.53 (0.21, 1.32)
Specific condition - Other	Fredwall, 2021	Epilepsy	Linked with counseling	117	0.33 (0.11, 0.97)
Specific condition - Other	Zhu, 2021	Rheumatology patients	Follow-up phone required	2779	1.44 (0.90, 2.29)
Specific condition - Other	Rowe, 2021	Cardiology	Follow-up appointment	1445	1.50 (1.11, 2.02)
Specific condition - Other	Li, 2021	Ophthalmology	Need in-person review	854	0.15 (0.11, 0.22)
Specific condition - Other	Offiah, 2022	Cardiology patients	Return clinic	1716	0.60 (0.48, 0.77)
Specific condition - Other	Metcalfe, 2023	Otology referral	Patients followed-up	150	5.80 (2.87, 11.72)
Specific condition - Other	Mossack, 2022	Overactive bladder	>1 follow up	48	2.03 (0.56, 7.31)
Specific condition - Other	Mossack, 2022	Overactive bladder	90 day follow-up	48	2.40 (0.67, 8.58)
**Change in therapy**					
General medical care -All Ages	Wabe, 2022	General practice	≥1 medication prescribed	13608216	0.76 (0.76, 0.76)
Specific condition - Other	Mair, 2021	Rheumatology patients	Start new antirheumatic drugs	550	0.53 (0.28, 1.00)
Specific condition - Other	Mair, 2021	Rheumatology patients	Increased dose	550	0.55 (0.24, 1.27)
Specific condition - Other	Zhu, 2021	Rheumatology patients	Immunosuppressive therapy change	2779	0.78 (0.65, 0.92)
Specific condition - Other	Offiah, 2022	Cardiology patients	Medication changes	1716	0.41 (0.31, 0.53)
Specific condition - Other	Lindhagen,	Inflammatory bowel disease	Increased medication	1759	1.05 (0.84, 1.32)
Specific condition - Other	Lindhagen,	Inflammatory bowel disease	Decreased medication	1759	0.97 (0.66, 1.44)
Specific condition - Other	Severino, 2023	Heart Failure	Change in therapy	184	1.34 (0.63, 2.83)
Specific condition - Other	Piga, 2022	Heart Failure	Treatment adjusted	212	1.14 (0.56, 2.34)
Specific condition - Other	Piga, 2022	Heart Failure	Treatment tapering/cessation	212	1.25 (0.49, 3.15)
Specific condition - Other	Mossack, 2022	Overactive bladder	Treatment change	48	0.46 (0.13, 1.63)
**Treatment adherence**					
Behavioral/Mental health	Ripp, 2022	Behavioral pediatrics	Completed followup visits	1431	1.57 (1.23, 2.00)
Behavioral/Mental health	Fortier, 2022	U.S. Veterans	Treatment completers	74	1.47 (0.47, 4.62)
Behavioral/Mental health	Cunningham, 2022	Opioid use disorder	Treatment retention	107	1.89 (0.83, 4.31)
Behavioral/Mental health	Frost, 2022	Eye movement desensitization	Treatment retention	4338	1.31 (1.12, 1.53)
Behavioral/Mental health	Gainer, 2022	Substance use disorder	Treatment engagement	457	2.12 (1.05, 4.28)
Behavioral/Mental health	Gainer, 2022	Substance use disorder	Treatment engagement	322	5.40 (1.92, 15.20)
General medical care - Adults	Griebeler, 2022	Overweight or obese	Full medication compliance	54	1.33 (0.37, 4.82)
General medical care -All Ages	Zimmerman, 2021	General	Completed treatment	414	1.63 (1.08, 2.47)
Specific condition - Other	McCoy, 2022	Pediatric otolaryngology	Surgery performed	172	1.20 (0.63, 2.30)
Specific condition - Other	Baughman, 2021	Diabetes	Adherent to treatment	63722	0.84 (0.80, 0.88)
Specific condition - Other	Mossack, 2022	Overactive bladder	Medication adherence	48	1.18 (0.38, 3.67)
**Up to date labs**					
Specific condition - Other	Parise, 2021	Type-1 diabetes	Continuous glucose monitoring	209	72.47 (26.27, 199.89)
Specific condition - Other	Liu, 2021	Gastroenterology/Rheumatology	Completed pathology test/consultation	1074	0.49 (0.36, 0.68)
Specific condition - Other	Liu, 2021	Gastroenterology/Rheumatology	Completed radiology test/consultation	640	0.38 (0.26, 0.56)
Specific condition - Other	Reddy, 2021	Cancer patients	Laboratory testing	1576	0.28 (0.22, 0.36)
Specific condition - Other	Reddy, 2021	Cancer patients	Diagnostic imaging	1576	0.30 (0.21, 0.44)
Specific condition - Other	Reddy, 2021	Cancer patients	Procedures	1576	0.29 (0.11, 0.79)
Specific condition - Other	Lindhagen, 2022	Inflammatory bowel disease	Surveillance colonoscopy	1724	0.55 (0.38, 0.80)
Specific condition - Other	Szigety, 2022	Genetic conditions	Genetic testing was recommended	4327	1.59 (1.38, 1.84)
Specific condition - Other	Szigety, 2022	Genetic conditions	Test completion	4327	0.86 (0.76, 1.00)
Pregnancy	Arias, 2022	Postpartum	Completed glucose tolerance test	104	0.99 (0.37, 2.68)

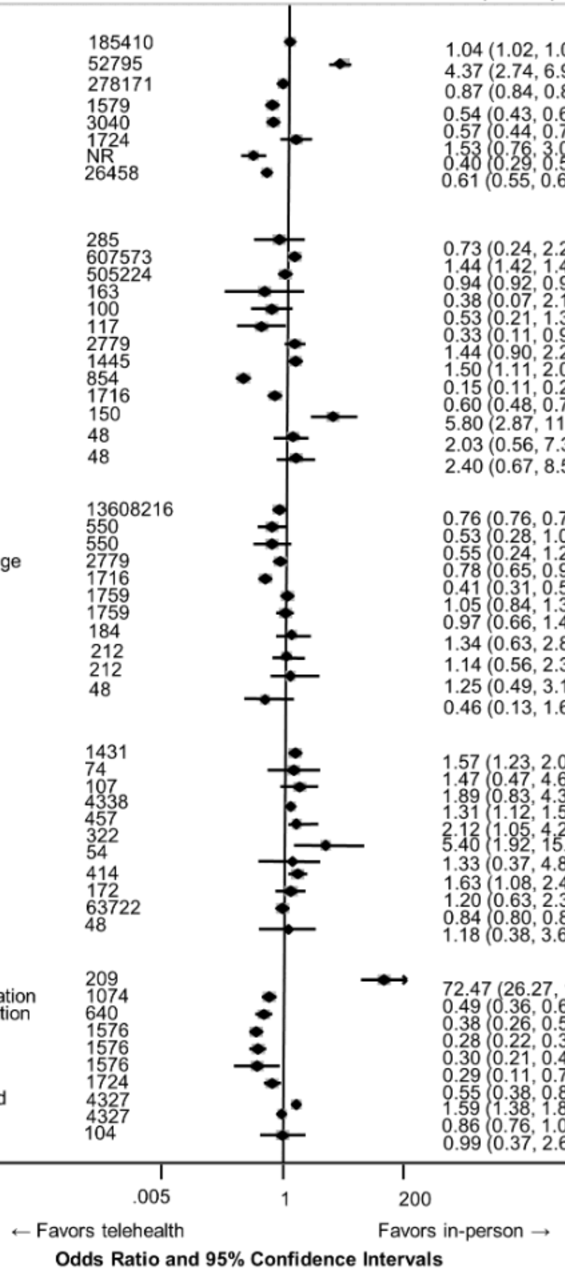

CI=confidence interval; ED/ER=emergency department/emergency room; HHT=hereditary hemorrhagic telangiectasia; N=sample size; NICU=neonatal intensive care unit; NR=not reported; OBGYN=obstetrics and gynecology; OR=odds ratio

* Included studies in each graph reported categorical data from which an odds ratio was calculated. Other studies did not report categorical data and odds ratios could not be calculated.

Supplementary References

1. Adams AM, Wu H, Zhang FR, Wajsberg JR, Bruney TL. Postpartum Care in the Time of COVID-19: The Use of Telemedicine for Postpartum Care. Telemed J E Health. 2023;29(2):235-41.
2. Adepoju OE, Chae M, Liaw W, Angelocci T, Millard P, Matuk-Villazon O. Transition to telemedicine and its impact on missed appointments in community-based clinics. Ann Med. 2022;54(1):98-107.
3. Afonso Nogueira M, Ferreira F, Raposo AF, Mónica L, Simões Dias S, Vasconcellos R, et al. Impact of telemedicine on the management of heart failure patients during coronavirus disease 2019 pandemic. ESC Heart Fail. 2021;8(2):1150-5.
4. Aiken A, Lohr PA, Lord J, Ghosh N, Starling J. Effectiveness, safety and acceptability of no-test medical abortion (termination of pregnancy) provided via telemedicine: a national cohort study. Bjog. 2021;128(9):1464-74.
5. Arias MP, Wang E, Leitner K, Sannah T, Keegan M, Delferro J, et al. The impact on postpartum care by telehealth: a retrospective cohort study. Am J Obstet Gynecol MFM. 2022;4(3):100611.
6. Barequet D, Gutfreund S, Goldstein M, Loewenstein A, Gamzu R, Varssano D. Evaluation of a Telemedicine Model for Following Keratoconus Patients in the Era of COVID-19 Pandemic. Telemed J E Health. 2021.
7. Baughman D, Zain A, Waheed A. Patient Adherence to Hemoglobin A1c Testing Recommendations in Telemedicine and In-Office Cohorts During COVID-19. JAMA Netw Open. 2021;4(9):e2127779.
8. Borgen I, Romney MC, Redwood N, Delgado B, Alea P, George BH, et al. From Hospital to Home: An Intensive Transitional Care Management Intervention for Patients with COVID-19. Popul Health Manag. 2021;24(1):27-34.
9. Boshara AI, Patton ME, Hunt BR, Glick N, Johnson AK. Supporting Retention in HIV Care: Comparing In-Person and Telehealth Visits in a Chicago-Based Infectious Disease Clinic. AIDS Behav. 2022:1-7.
10. Bryson AE, Milliren CE, Borzutzky C, Golub SA, Pitts SAB, DiVasta AD. Telemedicine for Adolescent and Young Adult Long-Acting Reversible Contraception Follow-Up Care amidst a Global Pandemic. J Pediatr Adolesc Gynecol. 2023;36(1):51-7.
11. Carlberg DJ, Bhat R, Patterson WO, Zaatari S, Chandra V, Kolkin A, et al. Preliminary Assessment of a Telehealth Approach to Evaluating, Treating, and Discharging Low-Acuity Patients With Suspected COVID-19. J Emerg Med. 2020;59(6):957-63.
12. Casariego-Vales E, Blanco-López R, Rosón-Calvo B, Suárez-Gil R, Santos-Guerra F, Dobao-Feijoo MJ, et al. Efficacy of Telemedicine and Telemonitoring in At-Home Monitoring of Patients with COVID-19. J Clin Med. 2021;10(13).
13. Chen K, Zhang C, Gurley A, Akkem S, Jackson H. Appointment Non-attendance for Telehealth Versus In-Person Primary Care Visits at a Large Public Healthcare System. J Gen Intern Med. 2023;38(4):922-8.
14. Clark A, Jung E, Prusky C, Shah BR, Halperin IJ. An Evaluation of Virtual Care for Gestational Diabetes Using the Quadruple Aim Framework: Assessment of Patient and Provider Experience, Cost and Clinical Outcomes. Can J Diabetes. 2022.
15. Cobo-Calvo A, Zabalza A, Río J, Arrambide G, Otero-Romero S, Tagliani P, et al. Impact of COVID-19 pandemic on frequency of clinical visits, performance of MRI studies, and therapeutic choices in a multiple sclerosis referral centre. J Neurol. 2022;269(4):1764-72.
16. Cunningham CO, Khalid L, Deng Y, Torres-Lockhart K, Masyukova M, Thomas S, et al. A comparison of office-based buprenorphine treatment outcomes in Bronx community clinics before versus during the COVID-19 pandemic. J Subst Abuse Treat. 2022;135:108641.
17. Cvietusa PJ, Goodrich GK, Steiner JF, Shoup JA, King DK, Ritzwoller DP, et al. Transition to virtual asthma care during the COVID pandemic:An observational study. J Allergy Clin Immunol Pract. 2022.
18. D'Anna L, Ellis N, Bentley P, Brown Z, Halse O, Jamil S, et al. Delivering telemedicine consultations for patients with transient ischaemic attack during the COVID-19 pandemic in a comprehensive tertiary stroke centre in the United Kingdom. Eur J Neurol. 2021;28(10):3456-60.
19. Duryea EL, Adhikari EH, Ambia A, Spong C, McIntire D, Nelson DB. Comparison Between In-Person and Audio-Only Virtual Prenatal Visits and Perinatal Outcomes. JAMA Netw Open. 2021;4(4):e215854.

20. Fortier CB, Currao A, Kenna A, Kim S, Beck BM, Katz D, et al. Online Telehealth Delivery of Group Mental Health Treatment Is Safe, Feasible, and Increases Enrollment and Attendance in Post-9/11 U.S. Veterans. Behav Ther. 2022;53(3):469-80.
21. Fredwall M, Terry D, Enciso L, Burch MM, Trott K, Albert DVF. Short-term outcomes in pediatric and adolescent patients with psychogenic nonepileptic events seen by telemedicine during the COVID-19 pandemic. Epilepsy Behav. 2021;117:107739.
22. Frost MC, Zhang L, Kim HM, Lin LA. Use of and Retention on Video, Telephone, and In-Person Buprenorphine Treatment for Opioid Use Disorder During the COVID-19 Pandemic. JAMA Netw Open. 2022;5(10):e2236298.
23. Gaetani E, Agostini F, Di Martino L, Occhipinti D, Passali GC, Santantonio M, et al. Beneficial Effects of Remote Medical Care for Patients with Hereditary Hemorrhagic Telangiectasia during the COVID-19 Pandemic. J Clin Med. 2021;10(11).
24. Gainer DM, Wong C, Embree JA, Sardesh N, Amin A, Lester N. Effects of Telehealth on Dropout and Retention in Care among Treatment-Seeking Individuals with Substance Use Disorder: A Retrospective Cohort Study. Subst Use Misuse. 2023;58(4):481-90.
25. Gao C, Osmundson S, Malin BA, Chen Y. Telehealth Use in the COVID-19 Pandemic: A Retrospective Study of Prenatal Care. Stud Health Technol Inform. 2022;290:503-7.
26. Garmendia O, Monasterio C, Guzmán J, Saura L, Ruiz C, Salord N, et al. Telemedicine Strategy for CPAP Titration and Early Follow-up for Sleep Apnea During COVID-19 and Post-Pandemic Future. Arch Bronconeumol. 2021;57 Suppl 2:56-8.
27. Griebeler M, Butsch WS, Rodriguez P, Lomeli L, Kampert M, Makin V, et al. The Use of Telemedicine for Obesity Pharmacotherapy in Patients With Overweight or Obesity. Obesity (Silver Spring, Md.). 2022;30:45.
28. Hatef E, Lans D, Bandeian S, Lasser EC, Goldsack J, Weiner JP. Outcomes of In-Person and Telehealth Ambulatory Encounters During COVID-19 Within a Large Commercially Insured Cohort. JAMA Netw Open. 2022;5(4):e228954.
29. Hughes Z, Simkowski J, Mendapara P, Fink N, Gupta S, Youmans Q, et al. Racial and Socioeconomic Differences in Heart Failure Hospitalizations and Telemedicine Follow-up During the COVID-19 Pandemic: Retrospective Cohort Study. JMIR Cardio. 2022;6(2):e39566.
30. Jazayeri A, Dinh JV, Eseonu D, Hollier JM, Shneider BL. Assessment of telemedicine versus in-person care in managing abdominal pain in children during the COVID-19 pandemic. Journal of telemedicine and telecare. 2022:1357633X221125836.
31. Kablinger AS, Gatto AJ, O'Brien VC, Ko H, Jones S, McNamara RS, et al. Effects of COVID-19 on Patients in Adult Ambulatory Psychiatry: Using Patient-Rated Outcome Measures and Telemedicine. Telemed J E Health. 2022.
32. Kerestes C, Murayama S, Tyson J, Natavio M, Seamon E, Raidoo S, et al. Provision of medication abortion in Hawai'i during COVID-19: Practical experience with multiple care delivery models. Contraception. 2021;104(1):49-53.
33. Khosla K, Suresh S, Mueller A, Perdigao JL, Stewart K, Duncan C, et al. Elimination of racial disparities in postpartum hypertension follow-up after incorporation of telehealth into a quality bundle. Am J Obstet Gynecol MFM. 2022;4(3):100580.
34. Klain M, Nappi C, Maurea S, De Risi M, Volpe F, Caiazzo E, et al. Management of differentiated thyroid cancer through nuclear medicine facilities during Covid-19 emergency: the telemedicine challenge. Eur J Nucl Med Mol Imaging. 2021;48(3):831-6.
35. Kolb CM, Born K, Banker K, Barth P, Aaronson NL. Comparing telehealth with office-based visits for common pediatric otolaryngology complaints. Int J Pediatr Otorhinolaryngol. 2021;145:110712.
36. Korycinski S, Metcalf D, Keteyian C. Effectiveness of a telephone-based nursing intervention to reduce hospital utilization by COVID-19 patients. Public Health Nurs. 2022.
37. Levinson CA, Spoor SP, Keshishian AC, Pruitt A. Pilot outcomes from a multidisciplinary telehealth versus in-person intensive outpatient program for eating disorders during versus before the Covid-19 pandemic. Int J Eat Disord. 2021;54(9):1672-9.

38. Li JO, Thomas AAP, Kilduff CLS, Logeswaran A, Ramessur R, Jaselsky A, et al. Safety of video-based telemedicine compared to in-person triage in emergency ophthalmology during COVID-19. EClinicalMedicine. 2021;34:100818.
39. Lindhagen S, Karling P. A more frequent disease monitorering but no increased disease activity in patients with inflammatory bowel disease during the first year of the SARS-CoV-2 pandemic. A retrospective study. Scand J Gastroenterol. 2022;57(2):169-74.
40. Liou H, Lane C, Huang C, Mookadam M, Joseph M, Hecker DuVal J. Eye Movement Desensitization and Reprocessing in a Primary Care Setting: Assessing Utility and Comparing Efficacy of Virtual Versus In-Person Methods. Telemed J E Health. 2022;28(9):1359-66.
41. Liu TL, Yeo AL, Ravi A, Patabendige G, Lim TW, Bell S, et al. The impact of COVID-19 telehealth on outpatient test completion. Intern Med J. 2021.
42. Mair J, Woolley M, Grainger R. Abrupt change to telephone follow-up clinics in a regional rheumatology service during COVID-19: analysis of treatment decisions. Intern Med J. 2021;51(6):960-4.
43. Mathews CP, Convoy S, Heyworth L, Knisely M. Evaluation of the Use of Telehealth Video Visits for Veterans With Chronic Pain. Pain Manag Nurs. 2022;23(4):418-23.
44. McCoy JL, Shaffer AD, Dohar JE. Pediatric otolaryngology telemedicine amid a pandemic - And beyond. Int J Pediatr Otorhinolaryngol. 2022;153:111014.
45. McNamara A, Zhao M, Lee SY. Evaluating the primary care clinical pharmacist visit transition to telehealth during the COVID-19 pandemic by comparing medication related problems from telehealth visits and in-person visits. J Am Coll Clin Pharm. 2021;4(8):914-23.
46. Metcalfe C, Gaskell P, McLelland T, Patel S, Muzaffar J, Dalton L, et al. A retrospective cohort study of telephone versus face-to-face clinics for the management of new otology referrals. Eur Arch Otorhinolaryngol. 2023;280(4):1677-82.
47. Minsky NC, Pachter D, Zacay G, Chishlevitz N, Ben-Hamo M, Weiner D, et al. Managing Obesity in Lockdown: Survey of Health Behaviors and Telemedicine. Nutrients. 2021;13(4).
48. Mossack S, Inoyatov I, Fonseca P, Du C, Lee E, Ruan H, et al. A Randomized Controlled Trial Comparing Telemedicine Versus In-Person Office Visits for the Follow-Up of Overactive Bladder. Urogynecology (Phila). 2022;28(12):819-24.
49. Offiah G, O'Connor C, Waters M, Hickey N, Loo B, Moore D, et al. The impact of a virtual cardiology outpatient clinic in the COVID-19 era. Ir J Med Sci. 2022;191(2):553-8.
50. Ostberg N, Ip W, Brown I, Li R. Impact of telemedicine on clinical practice patterns for patients with chest pain in the emergency department. Int J Med Inform. 2022;161:104726.
51. Parise M, Tartaglione L, Cutruzzolà A, Maiorino MI, Esposito K, Pitocco D, et al. Teleassistance for Patients With Type 1 Diabetes During the COVID-19 Pandemic: Results of a Pilot Study. J Med Internet Res. 2021;23(4):e24552.
52. Phillips JC, Lord RW, Davis SW, Burton AA, Kirk JK. Comparing telehealth to traditional office visits for patient management in the COVID-19 pandemic: A cross-sectional study in a respiratory assessment clinic. J Telemed Telecare. 2021:1357633x21990197.
53. Piga M, Floris A, Congia M, Chessa E, Cangemi I, Cauli A. Telemedicine in rheumatology: high specificity and sensitivity of follow-up virtual video consultations during COVID-19 pandemic. Rheumatology (Oxford). 2022;61(5):1795-801.
54. Pinsker JE, Singh H, McElwee Malloy M, Constantin A, Leas S, Kriegel K, et al. A Virtual Training Program for the Tandem t:slim X2 Insulin Pump: Implementation and Outcomes. Diabetes Technol Ther. 2021;23(6):467-70.
55. Prato A, Maugeri N, Chiarotti F, Morcaldi L, Vicario CM, Barone R, et al. A Randomized Controlled Trial Comparing Videoconference vs. Face-to-Face Delivery of Behavior Therapy for Youths With Tourette Syndrome in the Time of COVID-19. Front Psychiatry. 2022;13:862422.
56. Reddy A, Arthur J, Dalal S, Hui D, Subbiah I, Wu J, et al. Rapid Transition to Virtual Care during the COVID-19 Epidemic: Experience of a Supportive Care Clinic at a Tertiary Care Cancer Center. J Palliat Med. 2021;24(10):1467-73.
57. Rene R, Cherson M, Rannazzisi A, Felter J, Silverio A, Cunningham AT. Transitioning from In-Person to Telemedicine Within Primary Care Behavioral Health During COVID-19. Popul Health Manag. 2022;25(4):455-61.

58. Ripp A, Steinway C, Katzow MW, Jan S, Chen J, Chen V. Telehealth Utilization and Follow-Up Visits in Developmental-Behavioral Pediatrics During the COVID-19 Pandemic in 2020. J Dev Behav Pediatr. 2022.
59. Rowe BSJ, Paratz ED, Fahy L, Prior DL, MacIsaac AI. Telehealth in Australian cardiology: Insight into factors predicting the use of telephone versus video during the COVID-19 pandemic. Intern Med J. 2021.
60. Ryskina KL, Shultz K, Zhou Y, Lautenbach G, Brown RT. Older adults' access to primary care: Gender, racial, and ethnic disparities in telemedicine. J Am Geriatr Soc. 2021.
61. Severino P, D'Amato A, Prosperi S, Magnocavallo M, Maraone A, Notari C, et al. Clinical Support through Telemedicine in Heart Failure Outpatients during the COVID-19 Pandemic Period: Results of a 12-Months Follow Up. J Clin Med. 2022;11(10).
62. Sevilis T, McDonald M, Avila A, Heath G, Gao L, O'Brien G, et al. Telestroke: Maintaining Quality Acute Stroke Care During the COVID-19 Pandemic. Telemed J E Health. 2022;28(4):481-5.
63. Shah VV, Villaflores CW, Chuong LH, Leuchter RK, Kilaru AS, Vangala S, et al. Association Between In-Person vs Telehealth Follow-up and Rates of Repeated Hospital Visits Among Patients Seen in the Emergency Department. JAMA Netw Open. 2022;5(10):e2237783.
64. Sharma E, Meade S, D'Errico F, Pavlidis P, Luber R, Zeki S, et al. The effects of COVID-19 on IBD prescribing and service provision in a UK tertiary centre. GastroHep. 2020;2(6):318-26.
65. Sohail M, Long D, Kay E, Levitan EB, Batey DS, Reed-Pickens H, et al. Role of Visit Modality in the HIV-Related No-Shows During the COVID-19 Pandemic: A Multisite Retrospective Cohort Study. AIDS Behav. 2023:1-10.
66. Sun CA, Perrin N, Maruthur N, Renda S, Levin S, Han HR. Predictors of Follow-Up Appointment No-Shows Before and During COVID Among Adults with Type 2 Diabetes. Telemed J E Health. 2022.
67. Szigety KM, Crowley TB, Gaiser KB, Chen EY, Priestley JRC, Williams LS, et al. Clinical Effectiveness of Telemedicine-Based Pediatric Genetics Care. Pediatrics. 2022;150(1).
68. Tarn DM, Hintz C, Mendez-Hernandez E, Sawlani SP, Bholat MA. Using Virtual Visits to Care for Primary Care Patients With COVID-19 Symptoms. J Am Board Fam Med. 2021;34(Suppl):S147-s51.
69. Tchang BG, Morrison C, Kim JT, Ahmed F, Chan KM, Alonso LC, et al. Weight Loss Outcomes With Telemedicine During COVID-19. Front Endocrinol (Lausanne). 2022;13:793290.
70. Wabe N, Thomas J, Sezgin G, Sheikh MK, Gault E, Georgiou A. Medication prescribing in face-to-face versus telehealth consultations during the COVID-19 pandemic in Australian general practice: a retrospective observational study. BJGP Open. 2022;6(1).
71. Walker B, Stoecker C, Shao Y, Nauman E, Fort D, Shi L. Telehealth and Medicare Type 2 Diabetes Care Outcomes: Evidence From Louisiana. Med Care. 2023;61(Suppl 1):S77-s82.
72. Watson N, Cox A, Sanmugarajah J, Dzienis M, Hughes I. Safety and efficacy of telephone clinics during the COVID-19 pandemic in the provision of care for patients with cancer. Intern Med J. 2021;51(9):1414-9.
73. Ye S, Anstey DE, Grauer A, Metser G, Moise N, Schwartz J, et al. The Impact of Telemedicine Visits on the Controlling High Blood Pressure Quality Measure During the COVID-19 Pandemic: Retrospective Cohort Study. JMIR Form Res. 2022;6(3):e32403.
74. Zayde A, Kilbride A, Kucer A, Willis HA, Nikitiades A, Alpert J, et al. Connection During COVID-19: Pilot Study of a Telehealth Group Parenting Intervention. Am J Psychother. 2021:appipsychotherapy20210005.
75. Zhao M, Qin D, Cataldo G, Sharma K, Dandwate N, Orencole MP, et al. Virtual multidisciplinary care for heart failure patients with cardiac resynchronization therapy devices during the Coronavirus Disease 2019 pandemic. Int J Cardiol Heart Vasc. 2021;34:100811.
76. Zhu W, De Silva T, Eades L, Morton S, Ayoub S, Morand E, et al. The impact of telerheumatology and COVID-19 on outcomes in a tertiary rheumatology service: a retrospective audit. Rheumatology (Oxford). 2021;60(7):3478-80.
77. Zimmerman M, Terrill D, D'Avanzato C, Tirpak JW. Telehealth Treatment of Patients in an Intensive Acute Care Psychiatric Setting During the COVID-19 Pandemic: Comparative Safety and Effectiveness to In-Person Treatment. J Clin Psychiatry. 2021;82(2).
